# Progress in health among regions of Ethiopia, 1990–2019: a subnational country analysis for the Global Burden of Disease Study 2019

**DOI:** 10.1016/S0140-6736(21)02868-3

**Published:** 2022-04-02

**Authors:** GBD 2019 Ethiopia Subnational-Level Disease Burden Initiative Collaborators, Awoke Misganaw, Mohsen Naghavi, Ally Walker, Alemnesh H Mirkuzie, Ababi Zergaw Giref, Tezera Moshago Berheto, Ebba Abate Waktola, John H Kempen, Getachew Tollera Eticha, Tsigereda Kifle Wolde, Dereje Deguma, Kalkidan Hassen Abate, Kedir Hussein Abegaz, Muktar Beshir Ahmed, Yonas Akalu, Addis Aklilu, Biresaw Wassihun Alemu, Mulusew A Asemahagn, Atalel Fentahun Awedew, Senthilkumar Balakrishnan, Tariku Tesfaye Bekuma, Addisu Shunu Beyene, Misrak Getnet Beyene, Yihienew Mequanint Bezabih, Biruk Tesfaye Birhanu, Tesfaye Yitna Chichiabellu, Berihun Assefa Dachew, Amare Belachew Dagnew, Feleke Mekonnen Demeke, Getu Debalkie Demissie, Meseret Derbew Molla, Nebiyu Dereje, Kebede Deribe, Abebaw Alemayehu Desta, Munir Kassa Eshetu, Tomas Y Ferede, Eyob Alemayehu Gebreyohannes, Abraham Geremew, Hailay Abrha Gesesew, Lemma Getacher, Scott D Glenn, Aregash Samuel Hafebo, Abdiwahab Hashi, Hamid Yimam Hassen, Simon I Hay, Diriba Fufa Hordofa, Dawit Hoyiso Huluko, Ayele Semachew Kasa, Getinet Kassahun Azene, Ermiyas Mulu Kebede, Hafte Kahsay Kebede, Bayew Kelkay, Samuel Z Kidane, Samson Mideksa Legesse, Wondimu Ayele Manamo, Yohannes Adama A Melaku, Endalkachew Worku Mengesha, Sisay Derso Mengesha, Hayimro Edemealem Merie, Abera M Mersha, Amanual Getnet Mersha, Mizan Kiros Mirutse, Ammas Siraj Mohammed, Hussen Mohammed, Salahuddin Mohammed, Henok Biresaw Netsere, Dabere Nigatu, Mohammed Suleiman Obsa, Daniel Bogale Odo, Muktar Omer, Lemma Demissie Regassa, Biniyam Sahiledengle, Mohammed Feyisso Shaka, Wondimeneh Shibabaw Shiferaw, Negussie Boti Sidemo, Abiy H Sinke, Yitagesu Sintayehu, Muluken Bekele Sorrie, Birkneh Tilahun Tadesse, Eyayou Girma Tadesse, Zemenu Tamir, Animut Tagele Tamiru, Amare Abera Tareke, Yonas Getaye Tefera, Yohannes Tekalegn, Ayenew Kassie Tesema, Tefera Tadele Tesema, Fisaha Haile Tesfay, Zemenu Tadesse Tessema, Tadesse Tilahun, Gebiyaw Wudie Tsegaye, Biruk Shalmeno Tusa, Geremew Tassew Weledesemayat, Taklo Simeneh Yazie, Yordanos Gizachew Yeshitila, Birhanu Wubale Yirdaw, Desalegn Tegabu Zegeye, Christopher J L Murray, Lia Tadesse Gebremedhin

## Abstract

**Background:**

Previous Global Burden of Diseases, Injuries, and Risk Factors Study (GBD) studies have reported national health estimates for Ethiopia. Substantial regional variations in socioeconomic status, population, demography, and access to health care within Ethiopia require comparable estimates at the subnational level. The GBD 2019 Ethiopia subnational analysis aimed to measure the progress and disparities in health across nine regions and two chartered cities.

**Methods:**

We gathered 1057 distinct data sources for Ethiopia and all regions and cities that included census, demographic surveillance, household surveys, disease registry, health service use, disease notifications, and other data for this analysis. Using all available data sources, we estimated the Socio-demographic Index (SDI), total fertility rate (TFR), life expectancy, years of life lost, years lived with disability, disability-adjusted life-years, and risk-factor-attributable health loss with 95% uncertainty intervals (UIs) for Ethiopia's nine regions and two chartered cities from 1990 to 2019. Spatiotemporal Gaussian process regression, cause of death ensemble model, Bayesian meta-regression tool, DisMod-MR 2.1, and other models were used to generate fertility, mortality, cause of death, and disability rates. The risk factor attribution estimations followed the general framework established for comparative risk assessment.

**Findings:**

The SDI steadily improved in all regions and cities from 1990 to 2019, yet the disparity between the highest and lowest SDI increased by 54% during that period. The TFR declined from 6·91 (95% UI 6·59–7·20) in 1990 to 4·43 (4·01–4·92) in 2019, but the magnitude of decline also varied substantially among regions and cities. In 2019, TFR ranged from 6·41 (5·96–6·86) in Somali to 1·50 (1·26–1·80) in Addis Ababa. Life expectancy improved in Ethiopia by 21·93 years (21·79–22·07), from 46·91 years (45·71–48·11) in 1990 to 68·84 years (67·51–70·18) in 2019. Addis Ababa had the highest life expectancy at 70·86 years (68·91–72·65) in 2019; Afar and Benishangul-Gumuz had the lowest at 63·74 years (61·53–66·01) for Afar and 64.28 (61.99-66.63) for Benishangul-Gumuz. The overall increases in life expectancy were driven by declines in under-5 mortality and mortality from common infectious diseases, nutritional deficiency, and war and conflict. In 2019, the age-standardised all-cause death rate was the highest in Afar at 1353·38 per 100 000 population (1195·69–1526·19). The leading causes of premature mortality for all sexes in Ethiopia in 2019 were neonatal disorders, diarrhoeal diseases, lower respiratory infections, tuberculosis, stroke, HIV/AIDS, ischaemic heart disease, cirrhosis, congenital defects, and diabetes. With high SDIs and life expectancy for all sexes, Addis Ababa, Dire Dawa, and Harari had low rates of premature mortality from the five leading causes, whereas regions with low SDIs and life expectancy for all sexes (Afar and Somali) had high rates of premature mortality from the leading causes. In 2019, child and maternal malnutrition; unsafe water, sanitation, and handwashing; air pollution; high systolic blood pressure; alcohol use; and high fasting plasma glucose were the leading risk factors for health loss across regions and cities.

**Interpretation:**

There were substantial improvements in health over the past three decades across regions and chartered cities in Ethiopia. However, the progress, measured in SDI, life expectancy, TFR, premature mortality, disability, and risk factors, was not uniform. Federal and regional health policy makers should match strategies, resources, and interventions to disease burden and risk factors across regions and cities to achieve national and regional plans, Sustainable Development Goals, and universal health coverage targets.

**Funding:**

Bill & Melinda Gates Foundation.

## Introduction

Ethiopia has taken crucial steps in the health sector, along with improvements in the economic and education sectors, to improve the country's health status in the past three decades. Primarily, four 5-year health sector development plans starting from 1996 and one 5-year health sector transformation plan starting from 2016 were operationalised, focusing on disease prevention, the decentralisation of health service delivery, the expansion of the primary health-care system, and public–private partnerships.^1^, ^2^ Ethiopia's health-care system has three tiers of care: primary, secondary, and tertiary.^3^ The national health-care delivery infrastructure has grown from a total of 2600 health facilities in 1997 to 21 154 facilities (which included 314 hospitals, 3678 health centres, and 17 162 health posts and private health facilities) in 2019. The health workforce has increased from 46 000 in 2007 to 159 545 in 2019.^1^, ^4^ Ethiopia's Health Extension Programme, an innovative community-based health programme, has improved essential health service coverage in underserved communities and rural areas.^5^ However, there have been challenges that include the suboptimal quality of health services, fragmented and weak health-care financing, poor pharmaceutical supply, absence of well established insurance schemes and benefit packages, poor intersectoral collaboration and public–private partnership coordination, and low health-care service use.^6^

**Research in context**

**Evidence before this study**

Ethiopia has been progressing over the last three decades on key health indicators, but the current conflict and the COVID-19 pandemic could negatively affect progress. The census, Demographic Health Surveys, and national surveys have shown the progress on death rates, life expectancy, fertility, child and maternal mortality, HIV, tuberculosis, malaria, and other infectious diseases at national and subnational levels. This work is among the first to present a comprehensive analysis of sociodemographic indicators and all major disease and risk factors across all the regional states of Ethiopia over an extended time period; previously, only national level estimates were available from Global Burden of Disease studies.

**Added value of this study**

For the first time, we provide a collaborative analysis of the effects of 369 diseases and injuries and 87 risk factors on premature mortality, disability, and disability-adjusted life-years (DALYs) for Ethiopia's regions and chartered cities from 1990 to 2019. This analysis highlights substantial health gains since 1990, as well as the disparities between regions and chartered cities, and the opportunity to reduce this burden of premature mortality by addressing specific diseases, risk factors, and sociodemographic factors.

**Implications of all the available evidence**

The results identify and rank potential priorities for action that would reduce premature mortality and provide relevant support for specific regions and chartered cities. Social and economic determinants of health are an overriding concern. There is a need for the socioeconomic development of poorer parts of the country, and for quality health-care services in underserved areas. Although mortality, years of life lost (YLLs), years lived with disability, and DALYs have all shown improvements over the past three decades, YLLs from both communicable and non-communicable diseases are still high in parts of Ethiopia.

Some studies and national statistics have shown Ethiopia's substantial progress in improving child survival, shown with key health indicators, reducing the maternal mortality rate and total fertility rate (TFR), and increasing life expectancy at national and regional levels.^7^, ^8^, ^9^, ^10^, ^11^, ^12^, ^13^ Previous Global Burden of Diseases, Injuries, and Risk Factors Study (GBD) national estimates have also indicated that Ethiopia has been successful in reducing morbidity and mortality related to infectious diseases, and maternal, neonatal, and nutritional deficiency diseases and injuries, despite unacceptably high maternal and neonatal mortality rates. However, the country's progress regarding non-communicable diseases (NCDs), including cardiovascular disease, diabetes, cancer, and chronic respiratory disease has been minimal.^14^, ^15^ Studies on NCD risk factors showed a high prevalence of hypertension, high blood glucose, obesity, harmful use of alcohol, smoking, and low fruit and vegetable consumption in Ethiopia.^16^ However, regional states in Ethiopia have undergone heterogeneous changes that would be expected to result in wide variation in progress in different parts of the country. National surveys have tried to examine this variation, but a comprehensive analysis of sociodemographic indicators and all major disease and risk factors across all regional states of Ethiopia for an extended time is not available to show the progress and disparities.

Ethiopia is the second most populous country in Africa (behind Nigeria), with a population estimated at 107·6 million people,^17^ having more than 80 different ethnic groups, and a diverse range of cultures and lifestyles.^18^ 79% of the population lives in rural areas, and 12–14% of the total population are pastoralists or agro-pastoralists.^19^ Ethiopia is composed of ten regions (Afar, Amhara, Benishangul-Gumuz, Gambella, Harari, Oromia, Somali, Sidama, the Southern Nations, Nationalities, and Peoples' [SNNP] region, and Tigray) and two chartered cities (Addis Ababa and Dire Dawa).^20^, ^21^ Oromia, Amhara, and the SNNP region are the most populous regions. The GBD 2019 study used subnational data to generate estimates of national and regional health progress in Ethiopia from 1990 to 2019, and measure disparities between regions and cities. These estimates can help local authorities to create benchmarks, measure health sector transformation plans and Sustainable Development Goal progress, set policies, and inform plans for local resource allocation. This manuscript was produced as part of the GBD Collaborator Network.

## Methods

### Geographical units

This collaborative subnational country analysis is part of the GBD 2019 study. A collaborative team from the Ethiopian Public Health Institute (EPHI) and the Institute for Health Metrics and Evaluation started historical geographical boundary mapping of Ethiopia's nine regions and two chartered cities using previous constitutions (1931, 1955, 1987, and 1995).^22^, ^23^ The team used woredas (the term for districts of Ethiopia) for the geographical boundary mapping of regions and cities, because woredas were stable government structures (compared with structures at levels higher or lower than the woreda) during political or government changes, and through the three census years (1984, 1994, and 2007; appendix pp 5–10). We used three census rounds^18^, ^24^, ^25^ to produce continuous population and demographic estimates for the regions and chartered cities between 1950 and 2019. In this analysis, the Sidama region was contained within the SNNP region.

### Outcomes and analyses

GBD 2019 used all available data sources and applied a spatiotemporal Gaussian process regression, cause of death ensemble model, Bayesian meta-regression tool, DisMod-MR 2.1, and other models to generate fertility, mortality, cause of death, and disability rates. The risk factor attribution estimations followed the general framework established for comparative risk assessment (appendix pp 11–12). GBD 2019 estimated life expectancy, TFR, Socio-demographic Index (SDI), incidence, prevalence, mortality, years of life lost (YLLs), years lived with disability (YLDs), and disability-adjusted life-years (DALYs) due to 369 diseases and injuries and 87 risk factors (the full list is available online), and decomposed life expectancy by causes of premature mortality.^26^, ^27^ The SDI is the geometric mean of 0 to 1 indices of the total fertility rate younger than 25 years, the mean education for those aged 15 years and older, and lag distributed income per person.^28^

All metrics were estimated separately for Ethiopia's nine regions and two cities and are presented with 95% uncertainty intervals (UIs). All rates presented are age-standardised rates per 100 000 population. We have summarised the GBD methods and techniques, including references for the full GBD methods (appendix pp 11–12). The GBD 2019 subnational results were reviewed with EPHI experts and GBD Ethiopia collaborators, a network of more than 700 Ethiopian researchers and health workers. GBD 2019 analyses were completed with Python version 3.6.2, Stata version 13, and R version 3.5.0 (appendix p 14). The statistical code used for GBD estimation is publicly available online.

### Role of the funding source

The funder of this study had no role in study design, data collection, data analysis, data interpretation, or the writing of the report.

## Results

### SDI, fertility, and life expectancy

**Figure 1:**
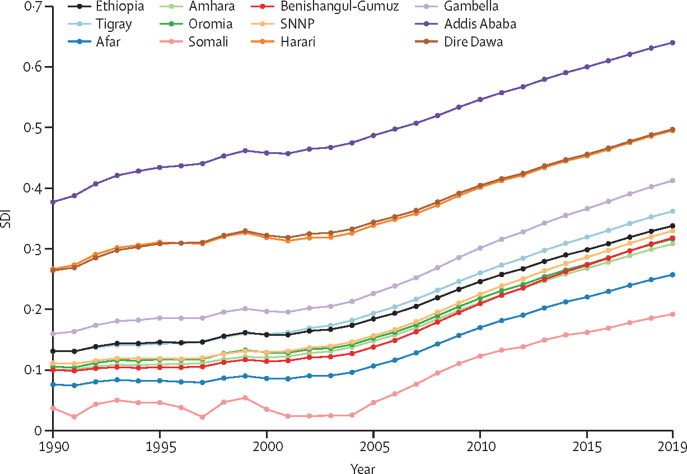
SDI time trend for nine regions and two cities in Ethiopia, 1990–2019 SDI=sociodemographic index. SNNP=Southern Nations, Nationalities, and Peoples' region.

**Figure 2:**
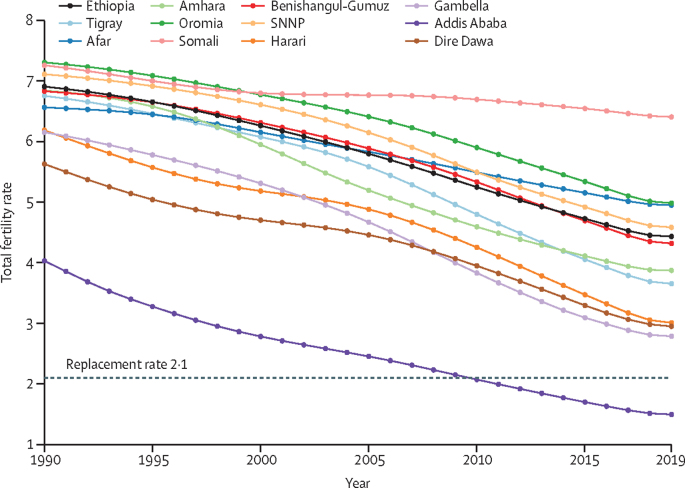
Time trend of total fertility rate in Ethiopia, 1990–2019 SNNP=Southern Nations, Nationalities, and Peoples' region.

In 2019, the SDI for Ethiopia was 0·34. Addis Ababa had the highest SDI (0·64), followed by Dire Dawa and Harari. Somali (0·19) and Afar (0·26) had the lowest SDIs. The SDI increased in all regions and cities between 1990 and 2019, yet inequality, as measured by the variation in SDI between regions and chartered cities, increased by 54% during that same period. The SDI of Ethiopia rose steadily from 0·13 in 1990 to 0·34 in 2019 (figure 1; appendix p 27). Nationally, the TFR declined from 6·91 (95% UI 6·59–7·20) in 1990 to 4·43 (4·01–4·92) in 2019. There was variation in TFR between regions and cities, ranging from a decline in Somali from 7·26 (6·88–7·61) in 1990 to 6·41 (5·96–6·86) in 2019, to a decline from 4·03 (3·58–4·51) to 1·50 (1·26–1·80) in Addis Ababa. In 2019, the TFR was highest in Somali and lowest in Addis Ababa. Dire Dawa, Gambella, Harari, Tigray, Amhara, and Benishangul-Gumuz had lower fertility rates than national estimates (figure 2; appendix pp 17–18).

**Figure 3:**
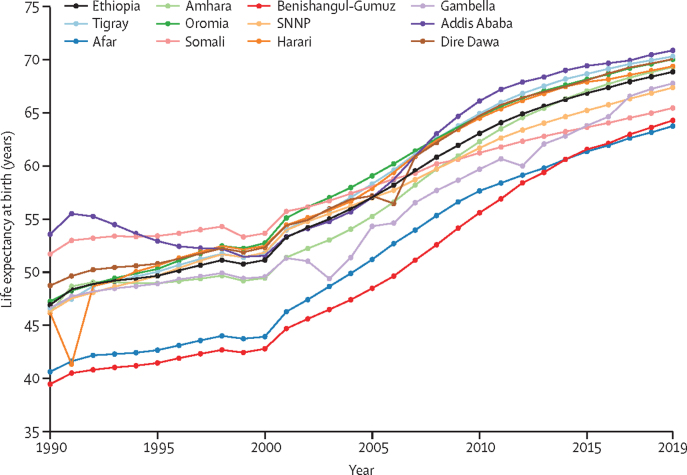
Time trend of life expectancy in Ethiopia for all sexes, 1990–2019 SNNP=Southern Nations, Nationalities, and Peoples' region.

Between 1990 and 2019, life expectancy at birth for all sexes combined improved in Ethiopia by 21·93 years (95% UI 21·80–22·07), increasing from 46·91 (95% UI 45·71–48·11) years in 1990 to 68·84 (67·51–70·18) years in 2019 (figure 3). Overall, in 2019, female life expectancy was higher than male life expectancy in all regions and cities except Afar and Benishangul-Gumuz, and this difference was statistically significant in the Amhara and Gambella regions (appendix p 19). Overall in Ethiopia, the increase in life expectancy at birth was 22·42 years for men, from 44·71 years (43·25–46·15) in 1990 to 67·13 years (65·12–69·20) in 2019, and 21·30 years for women, from 49·50 years (48·04–51·05) in 1990 to 70·80 years (69·24–72·27) in 2019. In 2019, the life expectancy for women was lowest in the Afar and Benishangul-Gumuz regions. Life expectancy inequality between regions and cities decreased in men by 81% (from 19·29 to 3·75), and in women by 44% (from 27·52 to 15·48) from 1990 to 2019 (appendix p 28).

The increase in absolute number of years of life expectancy at birth for all sexes varied between regions and cities, from 14 years in Somali to 25 years in Benishangul-Gumuz (appendix p 28). In 1990, the highest life expectancy was in Addis Ababa at 53·55 years (95% UI 51·67–55·41), and the lowest was in Benishangul-Gumuz at 39·44 years (37·29–41·37); the absolute difference between the highest and the lowest was 14·11 years. In 2019, the highest life expectancy was in Addis Ababa at 70·86 years (68·91–72·65), and the lowest was in Afar at 63·74 years (61·53–66·01). Life expectancy variation between regions and cities declined from 17·16 years in 1990 to 10·73 years in 2019 for women, and from 13·40 years in 1990 to 5·45 years in 2019 for men. In 2019, Addis Ababa, Dire Dawa, and Gambella had a high life expectancy in women with low TFR, whereas Somali, Benishangul-Gumuz, the SNNP region, and Afar had a low life expectancy in women with a high TFR (appendix p 29).

The increase in life expectancy across regions and cities was driven by declines in mortality due to HIV/AIDS, tuberculosis, diarrhoeal diseases, lower respiratory infections (LRIs), and war and conflict (table 1; appendix pp 22–26). Between 1990 and 2019, declines in age-standardised YLLs from HIV/AIDS, tuberculosis, diarrhoeal diseases, LRIs, and war and conflict each contributed 1–9 years of life expectancy gains among the different regions and cities (table 1, appendix pp 23–26).

**Table 1:** Change in life expectancy at birth in Ethiopia as a whole, and the nine regions and two cities in Ethiopia, by leading causes and conditions for all sexes, 1990–2019*

	**Ethiopia**	**Addis Ababa**	**Afar**	**Amhara**	**Benishangul-Gumuz**	**Dire Dawa**	**Gambella**	**Harari**	**Oromia**	**Somali**	**SNNP**	**Tigray**
HIV and AIDS	3·23	9·01	2·11	5·04	2·01	5·05	3·12	3·09	2·66	1·44	1·24	3·28
Tuberculosis	3·14	2·84	4·21	2·89	4·27	3·14	3·35	3·99	3·85	1·83	2·60	3·09
Diarrhoeal diseases	2·91	1·72	3·72	3·00	2·77	3·02	3·86	3·58	3·29	2·26	2·93	2·07
Lower respiratory infections	2·54	1·82	2·24	2·25	3·21	3·37	3·61	3·51	2·97	1·01	2·87	2·78
Conflict and war	2·06	3·62	1·08	3·78	1·38	1·35	1·30	1·28	1·64	1·35	1·32	1·40
Measles	1·70	0·40	1·23	1·96	2·05	1·25	1·85	1·08	1·50	1·75	2·13	1·28
Protein-energy malnutrition	1·17	0·50	1·82	0·88	1·73	1·19	1·16	0·99	1·48	1·06	1·23	1·39
Neonatal disorders	0·94	0·99	0·75	0·94	0·89	1·29	1·48	1·21	0·99	0·41	1·23	1·25
Meningitis	0·71	0·61	0·63	0·67	0·94	1·02	0·96	1·03	0·74	0·30	0·86	0·84
Stroke	0·69	0·77	0·75	0·79	0·83	0·85	0·68	0·99	0·69	0·29	0·57	0·93
Maternal disorders	0·63	0·34	1·14	0·65	1·37	0·43	0·36	0·49	0·65	0·80	0·50	0·85
Leishmaniasis	0·44	NA	NA	0·93	0·04	0·04	0·08	0·01	0·02	−0·01	0·23	2·68
Ischaemic heart disease	0·37	0·26	0·40	0·46	0·46	0·35	0·32	0·45	0·39	0·00	0·34	0·46
Cirrhosis and other chronic liver diseases	0·33	0·41	0·32	0·33	0·37	0·38	0·28	0·43	0·26	0·06	0·25	0·43
Congenital birth defects	0·31	0·19	0·21	0·34	0·36	0·60	0·59	0·55	0·26	0·09	0·52	0·36
Road injuries	0·30	0·24	0·33	0·29	0·37	0·38	0·39	0·58	0·34	0·07	0·32	0·37
Pertussis	0·28	0·25	0·17	0·27	0·46	0·67	0·72	0·46	0·23	0·13	0·49	0·18
Diabetes	0·27	0·19	0·24	0·26	0·28	0·28	0·22	0·31	0·33	0·07	0·28	0·38
Hypertensive heart disease	0·26	0·27	0·28	0·30	0·33	0·34	0·29	0·41	0·26	0·23	0·24	0·34
Tetanus	0·21	0·20	0·49	0·11	0·47	0·19	0·43	0·17	0·29	0·33	0·25	0·19

SNNP=Southern Nations, Nationalities, and Peoples' region. NA=not available.

* For HIV and AIDS, the data was from 2000 to 2019.

### Leading causes of death

In 2019, the all-cause age-standardised death rate was highest in Afar, at 1353·38 per 100 000 (95% UI 1195·69–1526·19). In 2019, the leading causes of age-standardised deaths in Ethiopia for all sexes combined were stroke, LRIs, ischaemic heart disease, diarrhoeal diseases, tuberculosis, cirrhosis and other chronic liver diseases, neonatal disorders (including preterm birth, sepsis, and encephalopathy), diabetes, HIV/AIDS, hypertensive heart disease, chronic obstructive pulmonary disease, and Alzheimer's disease and other dementias (table 2; appendix pp 30–38). Comparatively, Addis Ababa had the lowest death rates compared with any other region for neonatal disorders (12·89 per 100 000 [8·85–18·16]), diarrhoeal diseases (24·42 [9·55–44·34]), and LRIs (59·41 [49·81–71·44]). Although the national tuberculosis death rate was 60·90 (50·44–71·50), it was considerably higher in Afar (140·35 [113·05–171·19]), Somali (105·80 [78·40–148·83]), and Benishangul-Gumuz (100·81 [81·21–123·74]). The HIV/AIDS death rates in Afar (60·67 [40·56–86·47]), Dire Dawa (67·09 [40·51–103·01]), Gambella (91·96 [56·85–139·75]), and Addis Ababa (95·61 [71·23–125·38]) were well above the national rate of 33·56 (28·68–39·58). Death rates from protein-energy malnutrition were higher in Afar (35·56 per 100 000 [27·54–45·51]), Somali (33·34 [23·07–46·02]), and Benishangul-Gumuz (27·52 [21·07–34·18]) than the country rate (12·90 [9·77–16·70]). In Ethiopia, the age-standardised death rates were 90·18 per 100 000 (70·62–110·04) for stroke, 84·24 (62·56–105·75) for ischaemic heart disease, 36·00 (31·01–41·43) for diabetes, and 31·97 (17·85–53·09) for hypertensive heart disease (table 2; appendix pp 30–35).

**Table 2:** Age-standardised death, YLL, YLD, and DALY rates per 100 000 population for all causes and conditions combined and the leading ten causes in Ethiopia's regions and cities for all sexes, 2019

	**Ethiopia**	**Addis Ababa**	**Afar**	**Amhara**	**Benishangul-Gumuz**	**Dire Dawa**	**Gambella**	**Harari**	**Oromia**	**Somali**	**SNNP**	**Tigray**
**Death**												
All causes	993·52 (914·97–1070·55)	943·24 (836·87–1055·38)	1353·38 (1195·69–1526·19)	955·41 (855·37–1065·85)	1215·37 (1046·64-1394·05)	930·76 (824·93–1054·72)	1097·57 (985·20–1238·38)	981·41 (865·09–1101·04)	931·40 (842·89–1028·21)	1175·51 (1007·84–1366·86)	1091·62 (982·79–1211·40)	982·61 (869·47–1098·19)
Stroke	90·18 (70·62–110·04)	116·16 (96·78–137·59)	129·95 (101·08–160·22)	89·38 (63·85–113·96)	114·07 (87·35–144·52)	90·11 (66·51–113·88)	113·51 (82·92–142·58)	102·69 (78·39–126·79)	77·46 (57·53–98·33)	106·30 (76·92–137·63)	90·64 (70·53–112·85)	117·89 (91·08–147·91)
Lower respiratory infections	86·44 (75·36–97·65)	59·41 (49·81–71·44)	103·71 (86·62–122·62)	74·37 (59·40–91·02)	101·84 (84·02–121·72)	69·94 (56·55–84·00)	82·41 (68·39–97·19)	77·06 (63·09–92·52)	89·33 (75·91–103·18)	97·58 (79·26–118·67)	99·00 (83·81–116·65)	95·97 (67·34–123·71)
Ischaemic heart disease	84·24 (62·56–105·75)	116·47 (93·99–139·84)	118·22 (88·68–154·84)	82·64 (57·25–108·94)	103·98 (76·61–136·98)	84·28 (57·62–109·99)	106·02 (75·34–139·83)	91·96 (67·71–116·29)	74·99 (52·08–97·73)	93·89 (67·00–126·02)	86·85 (65·02–111·24)	84·63 (69·26–100·15)
Diarrhoeal diseases	76·45 (45·12–112·15)	24·42 (9·55–44·34)	101·11 (55·82–151·57)	83·64 (42·90–130·29)	106·56 (58·68–168·74)	49·08 (26·15–79·26)	66·20 (31·72–106·05)	52·24 (26·81–86·27)	71·44 (43·92–110·98)	99·69 (55·99–155·16)	83·72 (51·20–124·32)	61·69 (32·58–98·04)
Tuberculosis	60·90 (50·44–71·50)	45·20 (37·71–55·20)	140·35 (113·05–171·19)	59·77 (42·36–79·78)	100·81 (81·21–123·74)	47·07 (36·58–58·63)	76·77 (61·29–94·68)	61·04 (46·06–75·71)	52·46 (42·94–64·03)	105·80 (78·40–148·83)	67·41 (55·73–81·7)	50·18 (39·61–62·96)
Cirrhosis and other chronic liver diseases	52·18 (44·17–62·07)	47·21 (38·14–58·42)	63·37 (48·41–82·03)	50·30 (37·16–73·63)	55·80 (42·99–73·42)	47·32 (35·80–63·72)	55·58 (44·53–68·52)	50·43 (39·35–64·70)	49·92 (40·85–60·24)	55·48 (41·23–75·10)	62·08 (50·89–74·44)	33·76 (19·21–50·31)
Neonatal disorders	44·31 (34·92–56·99)	12·89 (8·85–18·16)	37·53 (28·99–49·26)	48·01 (37·44–62·34)	53·97 (41·45–71·03)	40·50 (30·27–53·58)	31·55 (23·57–41·73)	40·20 (30·05–53·01)	44·61 (35·47–56·81)	48·92 (38·58–62·57)	44·61 (34·60–58·11)	36·64 (19·77–61·88)
Diabetes	36·00 (31·01–41·43)	39·85 (33·00–47·63)	44·72 (37·44–53·71)	31·52 (25·13–39·14)	39·14 (32·12–47·98)	34·10 (26·70–42·03)	38·48 (31·52–46·44)	37·03 (29·18–45·21)	35·35 (29·16–41·80)	35·99 (28·49–44·91)	42·62 (35·42–50·85)	45·32 (36·11–56·03)
HIV and AIDS	33·56 (28·68–39·58)	95·61 (71·23–125·38)	60·67 (40·56–86·47)	40·12 (29·07–53·18)	26·19 (14·90–43·64)	67·09 (40·51–103·01)	91·96 (56·85–139·75)	38·84 (24·50–65·11)	23·86 (16·38–33·96)	42·96 (31·40–59·89)	20·22 (13·61–29·17)	30·77 (23·75–40·55)
Hypertensive heart disease	31·97 (17·85–53·09)	34·42 (16·91–54·07)	37·37 (20·89–59·95)	31·51 (16·76–54·86)	36·42 (19·54–59·42)	31·79 (16·06–56·11)	34·41 (18·72–62·25)	36·17 (18·43–61·89)	29·85 (16·12–51·79)	31·74 (16·68–56·83)	34·04 (20·02–53·15)	28·76 (6·81–76·63)
**YLLs**												
All causes	30 188·15 (27 335·78–33 522·76)	24 587·96 (21 376·63–28 479·55)	40 646·83 (35 269·66–46 519·61)	29 627·03 (26 153·31–33 252·03)	40 246·93 (34 763·93–46 458·09)	27 923·42 (24 318·73–31 972·61)	31 423·03 (27 436·21–36 073·92)	29 023·21 (25 052·82–33 498·29)	27 993·55 (24 822·94–31 877·11)	37 678·08 (32 787·59–43 171·12)	32 890·51 (29 184·45–37 155·55)	26 435·52 (22 998·71–30 181·61)
Neonatal disorders	3936 (3102·02–5062·36)	1144·89 (786·19–1613·71)	3334·17 (2575·70–4376·74)	4263·88 (3325·45–5535·56)	4794·17 (3682·23–6308·66)	3598·47 (2689·60–4760·63)	2802·72 (2094·70–3707·80)	3571·17 (2669·98–4709·61)	3962·85 (3151·45–5047·45)	4346·19 (3427·41–5558·73)	3963·41 (3074·49–5162·24)	2734·15 (2110·52–3602·85)
Diarrhoeal diseases	2679·41 (1823·93–3760·22)	706·94 (346·09–1160·00)	3028·74 (1858·68–4359·95)	2872·53 (1619·57–4669·36)	3468·05 (2075·83–5236·29)	1527·28 (863·89–2479·98)	1765·71 (941·39–2671·89)	1696·78 (949·97–2706·74)	2636·66 (1799·45–3752·16)	3189·06 (2085·43–4620·42)	2883·22 (1933·89–4093·83)	1885·83 (1160·09–2787·08)
Lower respiratory infections	2404·57 (2059·41–2833·31)	1285·69 (1065·07–1561·83)	2824·70 (2323·08–3414·07)	2016·49 (1551·49–2541·83)	3571·17 (2772·41–4510·79)	1832·81 (1407·35–2363·18)	1937·46 (1567·70–2344·65)	2060·45 (1595·62–2623·15)	2433·84 (2042·25–2879·55)	3236·48 (2537·45–4006·03)	2698·20 (2243·52–3250·28)	1977·15 (1593·94–2395·05)
Tuberculosis	1729·19 (1421·57–2049·86)	1257·74 (987·65–1637·78)	4224·43 (3303·11–5286·23)	1694·00 (1206·70–2252·30)	3122·54 (2515·90–3818·30)	1265·73 (961·97–1615·11)	2108·48 (1620·78–2668·38)	1708·90 (1268·87–2211·91)	1424·64 (1145·31–1764·27)	3114·68 (2316·65–4344·20)	1932·74 (1568·38–2350·92)	1353·68 (1052·73–1707·25)
Stroke	1639·45 (1277·27–1998·83)	2076·73 (1734·67–2511·62)	2643·28 (2063·50–3255·20)	1585·87 (1113·95–2048·27)	2265·59 (1735·78–2873·04)	1572·81 (1132·77–2021·77)	2089·66 (1505·93–2660·73)	1844·51 (1379·32–2326·34)	1382·54 (1033·51–1753·97)	2029·76 (1472·25–2610·22)	1694·69 (1329·04–2112·14)	2065·02 (1571·61–2618·60)
HIV and AIDS	1581·17 (1311·51–1935·43)	4381·86 (3213·40–5800·05)	2828·57 (1852·03–4134·58)	1922·00 (1387·50–2530·78)	1323·30 (734·37–2257·41)	3369·60 (2037·19–5146·92)	4584·14 (2776·25–7087·13)	2102·84 (1264·16–3429·05)	1154·69 (791·12–1689·22)	2024·87 (1418·98–2847·11)	954·79 (610·25–1395·80)	1592·44 (895·80–2417·13)
Ischaemic heart disease	1524·69 (1133·11–1925·66)	2119·12 (1686·63–2595·35)	2391·79 (1823·27–3112·12)	1469·00 (1013·35–1978·65)	2036·72 (1495·64–2644·13)	1460·62 (979·94–1944·49)	1968·05 (1389·57–2644·08)	1636·32 (1184·83–2099·02)	1317·64 (915·29–1719·34)	1786·75 (1267·81–2396·47)	1615·90 (1213·31–2075·90)	1691·52 (1175·78–2229·61)
Cirrhosis and other chronic liver diseases	1331·56 (1095·91–1625·94)	1227·62 (953·12–1582·71)	1693·13 (1249·58–2210·45)	1269·74 (912·09–1954·72)	1492·25 (1132·75–1986·18)	1171·79 (852·79–1648·64)	1400·83 (1101·59–1750·50)	1282·17 (959·15–1731·43)	1250·89 (1002·00–1549·27)	1451·64 (1072·82–2016·43)	1612·03 (1281·65–1974·90)	1119·70 (857·99–1431·60)
Congenital birth defects	735·83 (447·45–1200·08)	294·29 (148·31–529·45)	583·05 (363·12–963·96)	866·04 (441·58–1475·29)	1023·60 (524·59–1778·98)	614·30 (370·17–995·81)	444·15 (286·90–696·67)	607·49 (374·86–972·76)	689·72 (427·97–1142·24)	862·47 (474·74–1444·55)	745·48 (429·23–1259·86)	552·78 (362·72–840·05)
Diabetes	706·24 (606·76–813·68)	781·49 (643·35–962·41)	948·96 (782·94–1140·39)	609·84 (478·05–767·44)	822·09 (655·85–1010·38)	641·84 (496·93–804·18)	758·63 (610·59–934·72)	722·81 (567·26–908·61)	676·10 (557·15–807·10)	730·31 (576·84–909·92)	858·25 (705·57–1039·85)	727·59 (589·30–884·16)
**YLDs**												
All causes	10 802·18 (8088·52–13 958·50)	10 425·76 (7851·91–13 353·63)	11 290·94 (8406·90–14 586·17)	10 674·64 (7935·83–13 944·33)	10 773·61 (8074·54–13 898·43)	11 053·29 (8287·48–14 208·54)	11 340·23 (8536·87–14 745·69)	10 943·17 (8202·74–14 065·21)	10 788·48 (8029·18–13 971·66)	11 507·02 (8592·43–14 929·86)	10 810·59 (8100·11–14 049·02)	10 640·49 (8034·05–13 798·20)
Depressive disorders	808·70 (562·26–1115·11)	815·88 (565·46–1131·74)	784·23 (545·69–1097·05)	809·00 (558·62–1110·67)	796·47 (554·36–1096·28)	803·15 (559·64–1104·89)	802·73 (557·59–1097·05)	803·37 (557·81–1115·31)	815·62 (566·05–1125·68)	775·50 (538·06–1064·77)	807·20 (560·90–1108·03)	805·12 (560·02–1111·36)
Low back pain	700·06 (491·31–935·19)	636·61 (446·46–858·78)	676·16 (475·66–911·80)	693·74 (489·41–929·96)	699·34 (489·67–938·81)	650·50 (456·87–872·68)	697·75 (492·01–936·59)	649·95 (457·22–872·70)	714·15 (502·15–955·39)	680·59 (478·97–918·01)	708·70 (496·05–948·98)	696·20 (491·44–939·63)
Age-related and other hearing loss	552·70 (377·77–779·89)	497·84 (339·19–705·42)	570·96 (390·61–803·10)	556·77 (381·14–783·62)	555·14 (380·23–783·62)	522·77 (355·95–735·71)	534·19 (363·88–753·38)	521·43 (356·40–732·51)	555·32 (382·38–785·18)	581·23 (399·65–815·78)	548·43 (375·68–766·77)	546·33 (378·45–764·55)
Gynaecological diseases	470·78 (322·13–659·38)	481·29 (328·21–675·50)	455·22 (312·70–644·82)	469·82 (319·18–658·88)	449·66 (306·39–628·28)	470·41 (321·92–661·86)	495·18 (337·64–693·84)	460·07 (315·67–644·58)	466·96 (319·69–653·75)	465·32 (319·97–653·11)	474·78 (326·61–668·56)	481·39 (328·00–672·86)
Blindness and vision loss	420·96 (303·10–568·56)	377·40 (269·11–513·85)	345·37 (246·90–475·25)	398·11 (287·31–538·57)	340·08 (243·35–466·43)	460·07 (326·94–618·74)	433·44 (309·91–586·76)	480·91 (345·92–648·06)	398·76 (285·04–540·94)	361·65 (257·49–494·55)	561·13 (399·88–752·72)	373·54 (265·27–508·91)
Dietary iron deficiency	396·03 (261·75–579·23)	270·78 (170·88–410·00)	677·33 (445·84–1002·21)	279·26 (178·41–425·24)	295·35 (188·69–443·84)	655·76 (428·15–947·89)	384·96 (237·99–575·77)	498·52 (309·93–740·57)	419·97 (271·37–623·79)	880·51 (584·83–1267·92)	317·82 (203·95–467·85)	363·84 (229·32–542·94)
Headache disorders	353·16 (95·74–736·24)	356·48 (95·10–742·66)	361·15 (100·71–741·56)	354·09 (94·52–733·63)	366·38 (103·59–758·09)	369·69 (106·00–758·05)	371·41 (104·49–770·89)	368·03 (105·07–769·93)	353·14 (94·41–734·78)	359·97 (101·68–735·93)	341·02 (92·98–711·45)	371·54 (104·53–763·61)
Anxiety disorders	331·14 (230·89–451·16)	337·91 (236·50–467·10)	323·11 (222·71–450·05)	331·25 (227·31–458·40)	327·69 (228·42–452·45)	332·80 (232·93–452·71)	333·21 (231·46–459·70)	331·18 (228·85–454·05)	331·21 (231·77–452·36)	321·53 (223·56–443·42)	331·60 (231·11–453·17)	333·77 (234·71–463·46)
Neonatal disorders	307·52 (235·31–386·04)	415·97 (324·17–521·25)	343·94 (261·06–430·83)	296·22 (222·38–381·17)	244·58 (178·20–320·99)	320·54 (242·03–415·85)	332·68 (253·74–416·02)	311·07 (232·47–396·07)	316·12 (236·97–400·83)	294·52 (225·09–375·65)	278·85 (207·04–362·21)	340·95 (264·91–428·56)
Diabetes	273·67 (189·12–376·26)	304·60 (208·84–414·09)	329·58 (226·31–454·22)	252·09 (172·30–347·55)	305·70 (209·15–416·91)	272·21 (184·97–375·16)	286·69 (197·43–390·81)	294·59 (199·71–403·99)	267·03 (183·63–366·12)	292·86 (200·32–401·45)	301·49 (206·14–415·61)	275·06 (188·07–377·79)
**DALYs**												
All causes	40 990·33 (36 897·07–45 655·97)	35 013·73 (30 677·01–39 789·77)	51 937·77 (45 903·75–58 657·52)	40 301·67 (35 840·43–45 093·49)	51 020·53 (44 712·40–58 120·70)	38 976·71 (34 231·93–44 244·43)	42 763·26 (37 387·28–48 345·43)	39 966·38 (34 978·88–45 495·80)	38 782·03 (34 594·62–43 761·05)	49 185·09 (43 088·93–55 654·39)	43 701·09 (38 844·18–49 315·40)	37 076·01 (32 498·49–42 007·13)
Neonatal disorders	4243·53 (3407·87–5383·87)	1560·86 (1184·97–2048·87)	3678·11 (2912·53–4714·01)	4560·10 (3632·64–5842·72)	5038·75 (3911·71–6573·12)	3919·01 (3014·94–5092·33)	3135·40 (2420·92–4068·21)	3882·25 (2963·08–5013·78)	4278·97 (3442·66–5377·99)	4640·71 (3725·92–5857·79)	4242·27 (3354·08–5420·30)	3075·10 (2470·85–3937·54)
Diarrhoeal diseases	2898·12 (2035·97–3973·63)	883·62 (519·22–1368·20)	3238·45 (2060·54–4572·55)	3096·17 (1847·57–4883·50)	3719·35 (2338·40–5472·50)	1709·42 (1023·41–2655·86)	1985·51 (1155·12–2895·64)	1896·61 (1151·37–2918·70)	2852·77 (1992·59–3944·49)	3419·24 (2318·61–4840·49)	3110·23 (2148·20–4327·32)	2090·39 (1346·77–3000·98)
Lower respiratory infections	2415·69 (2067·80–2845·72)	1294·60 (1075·94–1572·77)	2837·33 (2338·11–3426·68)	2026·77 (1558·85–2552·84)	3583·45 (2784·79–4520·95)	1842·26 (1415·77–2373·02)	1947·45 (1579·22–2359·05)	2069·93 (1605·95–2631·56)	2445·48 (2052·39–2890·30)	3248·92 (2550·29–4023·32)	2709·11 (2251·06–3261·28)	1988·83 (1603·35–2405·10)
Tuberculosis	1853·05 (1539·82–2163·82)	1365·90 (1091·70–1739·66)	4394·22 (3500·95–5424·73)	1827·64 (1334·64–2397·19)	3282·33 (2672·85–3988·04)	1366·14 (1062·32–1718·69)	2230·80 (1742·13–2787·01)	1827·61 (1389·91–2335·15)	1535·95 (1263·04–1869·92)	3281·42 (2478·07–4529·64)	2059·99 (1690·93–2495·88)	1459·48 (1160·08–1813·77)
Stroke	1777·86 (1411·33–2139·63)	2288·71 (1939·56–2701·26)	2803·48 (2234·98–3407·61)	1718·41 (1236·20–2180·15)	2424·41 (1880·92–3038·22)	1738·91 (1294·21–2185·32)	2250·63 (1655·91–2814·25)	2023·00 (1549·06–2524·64)	1516·09 (1161·15–1880·54)	2176·19 (1619·33–2760·15)	1821·65 (1445·18–2239·56)	2214·29 (1709·96–2762·43)
HIV and AIDS	1699·06 (1423·54–2050·66)	4741·01 (3524·37–6230·74)	3009·81 (1996·96–4377·88)	2064·31 (1522·00–2726·20)	1427·33 (807·87–2364·19)	3639·48 (2268·80–5497·00)	4982·49 (3126·23–7529·25)	2325·68 (1429·12–3748·30)	1242·27 (852·62–1783·85)	2118·08 (1495·38–2961·89)	1023·02 (668·79–1488·18)	1723·00 (990·03–2584·84)
Ischaemic heart disease	1579·98 (1184·72–1974·00)	2180·60 (1745·92–2649·50)	2456·51 (1886·94–3173·22)	1522·95 (1064·49–2027·62)	2097·00 (1554·05–2731·21)	1517·08 (1036·11–1987·75)	2025·26 (1449·91–2693·88)	1692·41 (1239·79–2153·44)	1373·85 (973·66–1775·11)	1851·79 (1326·42–2450·78)	1666·45 (1265·22–2134·93)	1746·38 (1234·34–2282·81)
Cirrhosis and other chronic liver diseases	1341·44 (1106·26–1637·42)	1238·39 (963·18–1594·02)	1703·31 (1258·23–2219·46)	1279·48 (922·03–1965·74)	1502·28 (1142·48–1994·06)	1181·00 (860·54–1659·72)	1410·27 (1109·31–1758·63)	1291·36 (969·83–1740·35)	1260·50 (1015·22–1559·15)	1461·72 (1084·96–2028·88)	1622·55 (1292·62–1985·18)	1129·05 (866·49–1440·11)
Diabetes	979·91 (849·79–1115·68)	1086·09 (916·96–1276·16)	1278·55 (1076·67–1489·03)	861·93 (702·22–1040·72)	1127·80 (932·74–1340·89)	914·06 (750·84–1091·97)	1045·32 (870·92–1233·25)	1017·41 (828·70–1218·99)	943·12 (804·51–1093·68)	1023·16 (845·73–1228·82)	1159·74 (974·45–1365·04)	1002·65 (825·12–1181·70)

Data in parentheses are 95% uncertainty intervals. DALY=disability-adjusted life-year. SNNP=Southern Nations, Nationalities, and Peoples' region. YLD= years lived with disability. YLL= years of life lost.

### Leading causes of premature mortality

In 2019, the leading causes of premature mortality measured in YLLs for all sexes (neonatal disorders, diarrhoeal diseases, LRIs, tuberculosis, stroke, and HIV/AIDS) showed major declines in Ethiopia compared with 1990, and contributed substantial life expectancy gains over the previous 29 years (table 1; appendix pp 23–26). Ischaemic heart disease, cirrhosis, congenital defects, and diabetes were among the leading causes of premature mortality in Ethiopia (table 2; appendix pp 30–34). The all-cause age-standardised YLL rate in 2019 was the highest in Afar (40 646·83 YLLs [95% UI 35 269·66–46 519·61] per 100 000) and the lowest in Addis Ababa (24 587·96 YLLs [21 376·63–28 479·55] per 100 000), with the highest YLL rates of tuberculosis, stroke, ischaemic heart disease, and meningitis also observed in Afar (table 2). With high SDIs and life expectancy for all sexes, Addis Ababa, Dire Dawa, and Harari each showed low amounts of premature mortality from the five leading causes. Afar and Somali, regions with low SDIs and life expectancy for all sexes, had high premature mortality from the leading causes (appendix p 39).

The trend of YLL reduction varies at the subnational level for common causes such as neonatal disorders, diarrhoeal diseases, LRIs, tuberculosis, protein-energy malnutrition, and maternal disorders (table 2; appendix pp 30–39). In 2019, the age-standardised YLLs for all causes varied by more than 1·5 times between the regions and cities ranked highest and lowest by SDI: 24 587·96 (95% UI 21 376·63–28 479·55) in Addis Ababa versus 37 678·08 (32 787·59–43 171·12) in Somali (table 2; figure 1). Age-standardised YLL rates for Afar and Somali, the regions with the lowest SDI, were consistently higher than the national estimates for most leading conditions except stroke, ischaemic heart disease, hypertensive heart disease, diabetes, and HIV/AIDS. In 2019, HIV YLLs were more than 1·5 times higher in Addis Ababa compared with Afar or Somali. Age-standardised YLD rates varied much less than YLLs, with no significant disparity. DALY rates followed the pattern of YLLs (appendix pp 30–38).

The age-standardised premature mortality due to HIV/AIDS declined by 84·35% between 2000 and 2019, which contributed 3·23 years of life expectancy gained in Ethiopia during that same period. Declines in the premature mortality rate due to HIV/AIDS and gains in life expectancy varied by regions and cities, with a gain of 5–9 years of life expectancy observed in Amhara, Dire Dawa, and Addis Ababa. Life expectancy increased only 1·44 years in Somali, with a 65·50% decline in HIV premature mortality, and 1·24 years in SNNP, with a 77·16% decline between 2000 and 2019. Age-standardised premature mortality due to tuberculosis declined by 83·13%, which resulted in an increase in life expectancy of more than 3 years between 1990 and 2019 in Ethiopia as a whole and in most of the country's regions and cities. In Somali, the decline in tuberculosis mortality was 61·68%, the smallest in all regions, and contributed to lower gains in life expectancy (1·83 years) between 1990 and 2019. The LRI premature mortality rate declined by 76·40%, contributed 2·54 years' gain in life expectancy at the national level, and led to an increased life expectancy of more than 3 years in Benishangul-Gumuz, Gambella, Dire Dawa, and Harari between 1990 and 2019. The decline in premature mortality due to diarrhoeal diseases has contributed 2·91 years of life expectancy gain to the country; six regions gained 3 or more years of life expectancy. Premature mortality due to war and conflict declined by 99·00% in Ethiopia, which contributed 2·06 years of life expectancy for the country; the gain was higher for the Amhara region (3·78 years) and Addis Ababa city (3·62 years; Table 2, Table 3).

**Table 3:** Age-standardised years of life lost rates per 100 000 population for leading causes in Ethiopia in nine regions and two cities in 1990 and 2019* and percentage changes for all sexes

	**Ethiopia**	**Addis Ababa**	**Afar**	**Amhara**	**Benishangul-Gumuz**	**Dire Dawa**	**Gambella**	**Harari**	**Oromia**	**Somali**	**SNNP**	**Tigray**
**HIV and AIDS**												
2000	10 100·24	29 137·36	9995·00	15 469·43	7804·34	16 286·36	13 340·45	10 088·62	7799·95	5868·71	4180·19	10 210·47
2019	1581·17	4381·86	2828·57	1922·00	1323·30	3369·60	4548·14	2102·84	1154·69	2024·87	954·79	1592·44
Percentage change	−84·35%	−84·96%	−71·70%	−87·58%	−83·04%	−79·31%	−65·91%	−79·16%	−85·20%	−65·50%	−77·16%	−84·40%
**Tuberculosis**												
1990	10 252·20	8334·51	20 605·39	9265·17	18 240·52	9104·17	11 437·55	12 673·87	11 555·31	8127·46	9212·14	9898·26
2019	1729·19	1257·74	4224·43	1694·00	3122·54	1265·73	2108·48	1708·90	1424·64	3114·68	1932·74	1353·68
Percentage change	−83·13%	−84·91%	−79·50%	−81·72%	−82·88%	−86·10%	−81·57%	−86·52%	−87·67%	−61·68%	−79·02%	−86·32%
**Diarrhoeal diseases**												
1990	11 305·78	5305·32	18 143·19	11 662·52	16 799·22	9748·49	13 256·01	12 208·01	12 048·62	9718·97	11 723·50	8038·00
2019	2679·41	706·94	3028·74	2872·53	3468·05	1527·28	1765·71	1696·78	2636·66	3189·06	2883·22	1885·83
Percentage change	−76·30%	−86·67%	−83·31%	−75·37%	−79·36%	−84·33%	−86·68%	−86·10%	−78·12%	−67·19%	−75·41%	−76·54%
**Lower respiratory infections**												
1990	10 189·20	6362·21	10 993·55	8690·41	14 965·16	11 749·77	13 175·88	12 826·57	11 217·67	6286·94	11 633·02	10 346·49
2019	2404·57	1285·69	2824·70	2016·49	3571·17	1832·81	1937·46	2060·45	2433·84	3236·48	2698·20	1977·15
Percentage change	−76·40%	−79·79%	−74·31%	−76·80%	−76·14%	−84·40%	−85·30%	−83·94%	−78·30%	−48·52%	−76·81%	−80·89%
**Conflict and war**												
1990	5796·07	9598·16	3814·15	10 647·39	4558·37	3519·72	3527·98	3517·74	3616·65	3810·69	3675·22	3939·99
2019	8·80	9·15	5·03	16·69	5·10	5·08	5·21	5·05	8·00	4·71	4·97	6·83
Percentage change	−99·90%	−99·90%	−99·87%	−99·84%	−99·89%	−99·86%	−99·85%	−99·86%	−99·78%	−99·88%	−99·86%	−99·83%
**Measles**												
1990	5490·39	1226·36	4540·30	6262·85	7353·98	3736·66	5687·79	3361·29	4708·19	5732·76	6791·10	4028·48
2019	123·63	12·37	136·95	144·67	130·18	31·12	38·60	34·67	113·76	261·28	101·91	65·54
Percentage change	−97·75%	−98·99%	−96·98%	−97·69%	−98·23%	−99·17%	−99·32%	−98·97%	−97·58%	−95·44%	−98·50%	−98·37%
**Protein-energy malnutrition**												
1990	3944·34	1601·41	7861·05	2871·89	7033·10	3540·53	3578·94	3151·93	4814·79	4179·16	4029·77	4367·61
2019	446·03	228·99	923·33	233·46	1044·34	339·45	289·91	323·28	477·67	1133·40	436·11	258·09
Percentage change	−88·69%	−85·70%	−88·25%	−91·87%	−85·15%	−90·41%	−91·90%	−89·74%	−90·08%	−72·88%	−89·18%	−94·09%
**Neonatal disorders**												
1990	6964·76	4243·96	5790·65	7142·11	7891·87	7535·91	7340·27	7309·37	6903·15	5525·67	7748·01	6604·69
2019	3936·00	1144·89	3334·17	4263·88	4794·17	3598·47	2802·72	3571·17	3962·85	4346·19	3963·41	2734·15
Percentage change	−43·49%	−73·02%	−42·42%	−40·30%	−39·25%	−52·25%	−61·82%	−51·14%	−42·59%	−21·35%	−48·85%	−58·60%
**Meningitis**												
1990	2693·91	2019·06	3280·61	2447·91	4178·69	3270·90	3194·14	3551·72	2651·99	1869·40	3198·26	2854·69
2019	610·64	356·77	965·31	536·11	975·27	423·99	481·70	527·38	563·94	959·33	700·02	421·36
% change	−77·33%	−82·33%	−70·58%	−78·10%	−76·66%	−87·04%	−84·92%	−85·15%	−78·74%	−48·68%	−78·11%	−85·24%
**Maternal disorders**												
1990	2125·33	1053·41	5232·99	2005·24	5566·97	1265·00	951·66	1586·92	2120·09	3086·07	1735·35	2728·16
2019	439·96	151·91	1233·67	284·02	1094·40	246·24	160·01	279·98	435·84	876·60	480·66	430·55
Percentage change	−79·30%	−85·58%	−76·43%	−85·84%	−80·34%	−80·53%	−83·19%	−82·36%	−79·44%	−71·59%	−72·30%	−84·22%

SNNP=Southern Nations, Nationalities, and Peoples' region.

* For HIV and AIDS, the data was from 2000 to 2019.

### Leading causes of disability-adjusted life-years

In 2019, the leading causes of age-standardised DALYs in Ethiopia for all sexes combined were neonatal disorders, diarrhoeal diseases, LRIs, tuberculosis, and stroke. Nationally, the DALY rate for neonatal disorders was 4243·53 (95% UI 3407·87–5383·87) per 100 000 population, 2898·12 (2035·97–3973·63) for diarrhoeal diseases, and 2415·69 (2067·80–2845·72) for LRIs. Addis Ababa had the lowest DALYs comparatively for these three causes compared with other regions, with 1560·86 DALYs (1184·97–2048·87) per 100 000 for neonatal disorders, 883·62 (519·22–1368·20) for diarrhoeal diseases, and 1294·60 (1075·94–1572·77) for LRIs (table 2).

In 2019, the national DALYs were 1853·05 (95% UI 1539·82–2163·82) per 100 000 for tuberculosis, 1777·86 (1411·33–2139·63) for stroke, 1699·06 (1423·54–2050·66) for HIV/AIDS, 1579·98 (1184·72–1974·00) for ischaemic heart disease, 1341·44 (1106·26–1637·42) for cirrhosis, and 979·91 (849·79–1115·68) for diabetes in Ethiopia. Regionally, the tuberculosis DALY rates were highest in Afar (4394·22 [3500·95–5424·73] per 100 000), Benishangul-Gumuz (3282·33 [2672·85–3988·04]), and Somali (3281·42 [2478·07–4529·64]). The stroke DALYs were highest in Afar (2803·48 [2234·98–3407·61]). HIV/AIDS DALY rates were highest in Addis Ababa (4741·01 [3524·37–6230·74]), Dire Dawa (3639·48 [2268·80–5497·00]), and Gambella (4982·49 [3126·23–7529·25]; table 2). Afar (1002·98 [799·35–1243·99]), Benishangul-Gumuz (1004·40 [777·04–1269·52]), and Somali (999·09 [779·70–1267·92]) had substantially higher meningitis DALY rates than the country level of 640·20 (534·77–760·73; appendix p 38).

## Discussion

This study shows the progress in health across the nine regions and two chartered cities of Ethiopia. The SDI has steadily increased in all areas since 1990, but not equally in all localities. Except for the Somali region, we observed substantial declines in the TFR in all regions and cities. Life expectancy improved across all regions and cities. The inequality observed, with the highest life expectancy in Addis Ababa and the lowest life expectancy in Afar and Benishangul-Gumuz, narrowed in absolute terms from 14·2 years in 1990, to 7·1 years in 2019. Inequality in life expectancy is decreasing in Ethiopia, despite increasing SDI disparities. Afar, a predominantly pastoralist region, had the lowest life expectancy in 2019, and progressed less than any other region over the past three decades in reducing premature mortality due to tuberculosis, neonatal disorders, diarrhoeal diseases, HIV/AIDS, and LRIs. Somali showed less progress with lower respiratory disease, resulting in less life expectancy gain compared with all other regions and chartered cities and the country overall. In Ethiopia, substantial declines in premature mortality resulting from war and conflict have contributed to an additional 2 years of life expectancy for the country and greater gains for the Addis Ababa and Amhara regions in the past three decades.

The findings on SDIs here probably reflect Ethiopia's increasing efforts to improve all levels of education, increase contraceptive use, and promote economic growth.^13^, ^19^, ^29^ National TFR findings and the decline over this period were in line with UN world fertility estimates.^30^ The TFR pattern showed negative convergence (ie, increasing variation over the years), with 55% increasing variation between regions and cities from 1990 to 2019. In Addis Ababa, the TFR is less than the replacement level, which could be because of the postponement of marriage, a decreased incidence of marriage, and declines in marital fertility.^31^ High TFRs in Somali, Oromia, and Afar might suggest the need for improvements to reproductive health services, women's empowerment, and accessibility of family planning and contraceptive services in these regions.^13^, ^32^, ^33^

In 2019, Addis Ababa, Dire Dawa, and Gambella had higher life expectancies with lower TFRs in women, whereas Somali, Benishangul-Gumuz, the SNNP region, and Afar had lower life expectancies with higher TFRs in women, which could be due to the higher variation of fertility and maternal mortality between regions and cities.^13^ The peak variation in life expectancy between regions for all sexes around the year 2000 could be explained by high premature mortality due to HIV/AIDS.^34^ The decreasing inequality in life expectancy despite increasing SDI disparities reflects that the increasing life expectancy trend was mainly achieved through declines in under-5 years mortality and mortality reductions from common infectious diseases, neonatal conditions, and nutritional deficiency through effective interventions.^15^, ^35^ Regions and cities could further increase life expectancy by reducing cardiovascular diseases, cancer, diabetes, and chronic respiratory diseases, among other diseases.^14^, ^15^

Addis Ababa, Dire Dawa, Gambella, and Harari have shown notable progress in reducing premature mortality from HIV/AIDS: Gambella and Dire Dawa gained 3 years of life expectancy from 2000 to 2019 and Addis Abada gained 9 years. In 2019, HIV/AIDS still ranked as the first or second leading cause of premature mortality in these regions or cities. This finding highlights the need to intensify targeted HIV prevention and control interventions. In contrast, Somali and the SNNP region could adopt best practices from successful regions and cities to further reduce premature mortality from HIV/AIDS and gain an increased life expectancy.^36^, ^37^

In the case of Afar, the region could have gained more years of life expectancy over the study period by reducing premature mortality from tuberculosis, neonatal disorders, diarrhoeal diseases, HIV/AIDS, and LRIs. These diseases are the leading causes of premature mortality and require improving access to quality health care for pastoralist communities, addressing related risk factors, and improving socioeconomic development and domestic financing.^38^

In the Somali region, improved pastoral control efforts against tuberculosis could result in further gains in life expectancy. The region could benefit from prioritising LRIs, which are still the second leading cause of premature mortality, and learn from the success with LRIs of the Benishangul-Gumuz, Gambella, and Harari regions and Dire Dawa, which gained more than 3 years in life expectancy between 1990 and 2019. Somali needs to strengthen interventions on the leading risk factors of LRIs, such as improving childhood wasting and household air pollution, and the early diagnosis and treatment of *Streptococcus pneumoniae*, a leading cause of morbidity and mortality from LRIs.^39^ The recurrent hazards (climate, economic, and sociopolitical),^40^, ^41^ little coordination between federal and regional governments, insufficient adaptation of health interventions in pastoralist communities such as maternal and child health services, HIV and tuberculosis health-care services and health service use,^42^, ^43^ and persistent health service quality gaps could be explanations for the slow progress in health in Afar and Somali. Multisectoral transformation strategies, integrated with actions of the economy, education, and health sectors, and strong public–private partnerships are essential to tackle the social and structural drivers of health loss in these regions.^44^ In addition, the Afar and Somali regions would benefit from fully regional government-owned and implemented strategies that consider local contexts (ie, pastoral community and poor health-care access and quality, etc).

The results of this analysis highlight the substantial declines in communicable, maternal, neonatal, and nutritional diseases that have generally occurred with increases in socioeconomic development and subsequent increases in life expectancy and the correspondingly increased absolute burden of NCDs.^16^ This finding probably reflects the overall improvements in the primary health-care system, the effect of government-led collaborative efforts with development partners and community-based health programmes, and the effectiveness of health-care interventions.^45^, ^46^ Nevertheless, premature mortality from the leading causes are substantially higher in the pastoral and agropastoral regions Somali and Afar than any other region.

A high NCD burden in Afar could be related to heatstroke and high systolic blood pressure that could occur with salt farming and the high salt content in the Afar region, and also with the pastoralist diet, which includes dairy with a high fat content, but further data collection and investigation are needed. Most findings from Gambella were not expected, and drivers of inequalities between regions and cities were not clear. Moreover, particulate matter pollution, mainly household air pollution from solid fuels and ambient air pollution in urban areas, was the leading risk factor for high premature mortality and disability rates across regions and chartered cities. This finding provides insight to the relevance of access to electricity and improving housing conditions at the regional level in Ethiopia.

Ethiopia's progress in health could be negatively affected by the current conflict and war in Tigray, Amhara, Afar, Oromia, and Benishangul-Gumuz. The conflict is causing deaths and injuries, but also health consequences from the displacement of populations, violence, and the breakdown of health and social services. The COVID-19 pandemic could also negatively affect life expectancy and the overall health status because mortality from COVID-19 and non-COVID-19 diseases and health conditions is expected to increase, essential health services have been affected, and there have been economic effects across all regions. These consequences need urgent solutions by federal and regional governments because they threaten health gains nationally.

This study had several limitations. One was the change in the administrative structure of regions and cities during political government changes (appendix pp 5–10), and collaborative efforts were made in mapping the administrative boundary changes. This collaborative initiative has also helped the EPHI build a local data capacity and create in-country data repository systems. We used all available data identified through an extensive collaboration effort and involving more than 700 leading researchers and policy makers from Ethiopia. The generation of estimates and their interpretation have benefited from intensive subnational review workshops and consultative meetings with domain experts.

Health progress has been uneven between regions and cities, which requires further efforts to explore the underlying factors and solutions. All regions and cities, especially the low SDI regions of Afar and Somali, could benefit from improving equity and quality of education and health-care services, improving financing and efficiency of health-care services, and reducing poverty and unemployment to improve overall health status.^47^ NCDs were among the leading causes of death for regions and cities; this finding shows the need for strengthening the health-care system and integrating services, strengthening multisectoral collaboration, and allocating adequate resources. Essential health services could consider tailoring services particularly to pastoralist, agrarian, and urban populations (Afar, Somali, and Addis Ababa in particular) to minimise disparities between regions and cities and improve health outcomes for all Ethiopians. The findings of this study also help to revise the health extension packages (packages provided by health extension workers; there are five main components comprising 16 packages: disease prevention and control, family health, hygiene and environmental sanitation, health education and communication, and first aid) to include key risk factors and diseases in regional contexts. This analysis underscores that quality data availability and accessibility are essential to provide reliable estimates for Ethiopia. Further, lessons learned from this effort can be leveraged for subnational analyses and improvements to data availability and accessibility throughout sub-Saharan Africa.

For the **full list of diseases, injuries, and risk factors** see http://ghdx.healthdata.org/record/ihme-data/gbd-2019-cause-rei-and-location-hierarchies

For the **GBD statistical code** see http://ghdx.healthdata.org/gbd2019/code

## Data sharing statement

This paper summarises key findings from our analysis of GBD 2019 estimates. All subnational estimates are publicly available in our online tools (http://ghdx.healthdata.org/gbd-2019). Citations for the data used in this study can be accessed from the Global Health Data Exchange data input sources tool (http://ghdx.healthdata.org/gbd-2019/data-input-sources). Files containing all GBD 2019 subnational estimates are available on the Global Health Data Exchange website (http://ghdx.healthdata.org/gbd-2019) and can also be downloaded from the Global Health Data Exchange results tool (http://healthdata.org/gbd-results-tool). Additional results can be explored through online interactive visualisations (https://vizhub.healthdata.org/gbd-compare/).

## Declaration of interests

JHK reports grants or contracts from Sight for Soul as payment to their institution to support their work in Ethiopia. All other authors declare no competing interests.

## References

[1] Wama RG (2009). Reviewing Ethiopia's health system development. Japan Med Assoc J.

[2] Habtemariam MK, Semegn ST (2018). Setting health sector priorities: a brief overview of Ethiopia's experience. Cost Eff Resour Alloc.

[3] Wuneh AD, Medhanyie AA, Bezabih AM, Persson LÅ, Schellenberg J, Okwaraji YB (2019). Wealth-based equity in maternal, neonatal, and child health services utilization: a cross-sectional study from Ethiopia. Int J Equity Health.

[4] Ministry of Health - Ethiopia (2019). Health and health related indicators 2018–2019. https://e-library.moh.gov.et/library/wp-content/uploads/2021/07/Health-and-Health-Related-Indicators-2011.pdf.

[5] Koblinsky M, Tain F, Gaym A, Karim A, Carnell M, Tesfaye S (2010). Responding to the maternal health care challenge: The Ethiopian Health Extension Program. Ethiop J Health Dev.

[6] Ministry of Health - Ethiopia (February, 2021). Health Sector Transformation Plan II, 2020- 2025. https://e-library.moh.gov.et/library/wp-content/uploads/2021/07/HSTP-II.pdf.

[7] Kuruvilla S, Schweitzer J, Bishai D (2014). Success factors for reducing maternal and child mortality. Bull World Health Organ.

[8] Hailemariam D (2009). Sustaining gains in child health and HIV-related MDGs in Ethiopia: lessons from field research. Ethiop J Health Dev.

[9] Ruducha J, Mann C, Singh NS (2017). How Ethiopia achieved Millennium Development Goal 4 through multisectoral interventions: a Countdown to 2015 case study. Lancet Glob Health.

[10] UN Department of Economic and Social Affairs (2019). World Mortality 2019: highlights. https://www.un.org/en/development/desa/population/publications/pdf/mortality/WMR2019/WMR2019_Highlights.pdf.

[11] UNICEF, WHO, World Bank Group, UN (2020). Levels & trends in child mortality: report 2020, estimates developed by the UN Inter-agency Group for Child Mortality Estimation. https://www.unicef.org/media/79371/file/UN-IGME-child-mortality-report-2020.pdf.pdf.

[12] WHO (2020). World health statistics 2020: monitoring health for the SDGs, Sustainable Development Goals. https://apps.who.int/iris/handle/10665/332070.

[13] Central Statistical Agency, Inter City Fund (2016). Ethiopia demographic and health survey 2016. https://dhsprogram.com/pubs/pdf/FR328/FR328.pdf.

[14] Misganaw A, Melaku YA, Tessema GA (2017). National disability-adjusted life years (DALYs) for 257 diseases and injuries in Ethiopia, 1990-2015: findings from the global burden of disease study 2015. Popul Health Metr.

[15] Misganaw A, Haregu TN, Deribe K (2017). National mortality burden due to communicable, non-communicable, and other diseases in Ethiopia, 1990–2015: findings from the Global Burden of Disease Study 2015. Popul Health Metr.

[16] Bekele A, Michael MG, Shiferaw S, Abate E, Kifle T, Teferra S (2017). NCD risk factors on the rise in Ethiopia. Ethiop J Health Dev.

[17] Wang H, Abbas KM, Abbasifard M (2020). Global age-sex-specific fertility, mortality, healthy life expectancy (HALE), and population estimates in 204 countries and territories, 1950-2019: a comprehensive demographic analysis for the Global Burden of Disease Study 2019. Lancet.

[18] Central Statistical Agency (Ethiopia) (2007). Population and Housing Census 2007. https://www.statsethiopia.gov.et/census-2007-2/.

[19] Central Statistical Agency (Ethiopia) Population Projections for Ethiopia, 2007–2037. http://www.csa.gov.et/census-report/population-projections/category/368-population-projection-2007-2037.

[20] Keller EJ (2002). Ethnic federalism, fiscal reform, development and democracy in Ethiopia. Afr J Polit Sci.

[21] Yoseph A, Tamiso A, Ejeso A (2021). Knowledge, attitudes, and practices related to COVID-19 pandemic among adult population in Sidama Regional State, southern Ethiopia: a community based cross-sectional study. PLoS One.

[22] Beru T (2013). Brief history of the Ethiopian legal systems - past and present. Int J Leg Inf.

[23] Selassie BH (1966). Constitutional development in Ethiopia. J Afr Law.

[24] Central Statistical Agency (Ethiopia) (1984). Population and housing census 1984. https://www.statsethiopia.gov.et/census-1984/.

[25] Central Statistical Agency (Ethiopia) (1994). Population and housing census 1994. https://www.statsethiopia.gov.et/census-1994/.

[26] Vos T, Lim SS, Abbafati C (2020). Global burden of 369 diseases and injuries in 204 countries and territories, 1990–2019: a systematic analysis for the Global Burden of Disease Study 2019. Lancet.

[27] Murray CJL, Aravkin AY, Zheng P (2020). Global burden of 87 risk factors in 204 countries and territories, 1990–2019: a systematic analysis for the Global Burden of Disease Study 2019. Lancet.

[28] Dicker D, Nguyen G, Abate D (2018). Global, regional, and national age-sex-specific mortality and life expectancy, 1950–2017: a systematic analysis for the Global Burden of Disease Study 2017. Lancet.

[29] Cheru F, Cramer C, Oqubay A (2019).

[30] United Nations Department of Economic and Social Affairs (2020). World fertility and family planning 2020: highlights. https://www.un.org/en/development/desa/population/publications/pdf/family/World_Fertility_and_Family_Planning_2020_Highlights.pdf.

[31] Kinfu Y (2000). Below-repiacement fertility in Tropical Africa? Some evidence from Addis Ababa. J Aust Popul Assoc.

[32] Ethiopian Public Health Institute, Federal Ministry of Health (May, 2021). Ethiopia mini demographic and health survey 2019. https://dhsprogram.com/pubs/pdf/FR363/FR363.pdf.

[33] Admassie A, Abebaw D, von Braun J, Gatzweiler FW (2014). Marginality: addressing the nexus of poverty, exclusion and ecology.

[34] Hladik W, Shabbir I, Jelaludin A, Woldu A, Tsehaynesh M, Tadesse W (2006). HIV/AIDS in Ethiopia: where is the epidemic heading?. Sex Transm Infect.

[35] Freeman T, Gesesew HA, Bambra C (2020). Why do some countries do better or worse in life expectancy relative to income? An analysis of Brazil, Ethiopia, and the United States of America. Int J Equity Health.

[36] Jamieson D, Kellerman SE (2016). The 90 90 90 strategy to end the HIV Pandemic by 2030: can the supply chain handle it?. J Int AIDS Soc.

[37] Assefa Y, Gilks CF, Dean J (2019). Towards achieving the fast-track targets and ending the epidemic of HIV/AIDS in Ethiopia: successes and challenges. Int J Infect Dis.

[38] Assefa Y, Hill PS, Gilks CF, Admassu M, Tesfaye D, Van Damme W (2020). Primary health care contributions to universal health coverage, Ethiopia. Bull World Health Organ.

[39] Troeger C, Blacker B, Khalil IA (2018). Estimates of the global, regional, and national morbidity, mortality, and aetiologies of lower respiratory infections in 195 countries, 1990–2016: a systematic analysis for the Global Burden of Disease Study 2016. Lancet Infect Dis.

[40] Teka AM, Temesgen Woldu G, Fre Z (2019). Status and determinants of poverty and income inequality in pastoral and agro-pastoral communities: household-based evidence from Afar Regional State, Ethiopia. World Dev Perspect.

[41] Schmidt M, Pearson O (2016). Pastoral livelihoods under pressure: ecological, political and socioeconomic transitions in Afar (Ethiopia). J Arid Environ.

[42] Getnet F, Demissie M, Worku A (2021). Challenges in delivery of tuberculosis services in Ethiopian pastoralist settings: clues for reforming service models and organizational structures. BMC Health Serv Res.

[43] Dubale T, Mariam DH (2007). Determinants of conventional health service utilization among pastoralists in northeast Ethiopia. Ethiop J Health Dev.

[44] Bukhman G, Mocumbi AO, Atun R (2020). The *Lancet* NCDI Poverty Commission: bridging a gap in universal health coverage for the poorest billion. Lancet.

[45] Assefa Y, Tesfaye D, Damme WV, Hill PS (2018). Effectiveness and sustainability of a diagonal investment approach to strengthen the primary health-care system in Ethiopia. Lancet.

[46] Karim AM, Admassu K, Schellenberg J (2013). Effect of Ethiopia's health extension program on maternal and newborn health care practices in 101 rural districts: a dose-response study. PLoS One.

[47] Tesema MT, Braeken J (2018). Regional inequalities and gender differences in academic achievement as a function of educational opportunities: evidence from Ethiopia. Int J Educ Dev.

