# Supplementary material for: Progress in health among regions of Ethiopia, 1990–2019: a subnational country analysis for the Global Burden of Disease Study 2019
Source: Lancet. 2022 Apr 2;399(10332):1322–35. doi: 10.1016/S0140-6736(21)02868-3 (PMC8987934; doi:10.1016/S0140-6736(21)02868-3)
Supplement: Supplementary appendix [file mmc1.pdf]

THE LANCET

Supplementary appendix

This appendix formed part of the original submission and has been peer reviewed. We post it as supplied by the authors.

Supplement to: GBD 2019 Ethiopia Subnational-Level Disease Burden Initiative Collaborators. Progress in health among regions of Ethiopia, 1990–2019: a subnational country analysis for the Global Burden of Disease Study 2019. *Lancet* 2022; published online Mar, 13. https://doi.org/10.1016/S0140-6736(21)02868-3.

Supplementary appendix to "Progress in health among regions of Ethiopia, 1990–2019: a subnational country analysis for the Global Burden of Disease Study 2019"

This appendix provides supplemental figures and more detailed results for "Progress in health among regions of Ethiopia, 1990-2019: a subnational country analysis for the Global Burden of Disease Study 2019."

Table of Contents

List of figures and tables 3
Figures 3
Tables 3
Section 1: Ethiopia subnational population and demography mapping over time, 1942-2019 5
1.1 Afar region 6
1.2 Amhara region 6
1.3 Oromia region 7
1.4 Southern Nations and Nationalities and People region 8
1.5 Gambella region 8
1.6 Somali region 8
1.7 Benishangul-Gumuz region 8
1.8 Harari region 9
1.9 Addis Ababa chartered city 9
1.10 Dire Dawa chartered city 9
1.11 Tigray region 9
Section 2: Global Burden of Disease (GBD 2019) Method Summary 11
Section 3: Input Data Sources 12
Section 4: GBD Strengths and Limitations 12
Section 5: Statement of GATHER Compliance 14
Section 6: Author Contributions 15
Section 7: Figures and Tables 16
Figures 16
Figure S1. TFR for regions and chartered cities in Ethiopia, 2019 16
Figure S2. TFR percent change for regions and chartered cities in Ethiopia, 1990−2019 17
Figure S3. Life expectancy between regions and cities, females and males in Ethiopia, in 2019 18
Figure S4. Variation of life expectancy between regions and cities from 1980 to 2019 by sex, in Ethiopia 19
Figure S5. LE percent change by regions and chartered cities in Ethiopia, 1990-2019 20
Figure S6. Ranks of causes of death with age-standardised death rate per 100,000 for both sexes, in Ethiopia, 2019 21
Figure S7. Trends in YLL rates per 100 000 population for the seven causes with the highest national burden and LE improvement in Ethiopia and regions and chartered cities, 1990–2019 22
Tables 26
Table S1. SDI variance between 9 regions and 2 chartered cities in Ethiopia, 1990-2019 26

Table S2. LE for regions and chartered cities, both sexes, females and males in Ethiopia, 1990 to 2019..........27

Table S3. LE and TFR variance and percent change between regions and chartered cities in Ethiopia, 1990-2019 28

Table S4. Female LE and TFR in regions and chartered cities in Ethiopia, 2019 28

Table S5. Age-standardised death, YLL, YLD and DALY rates per 100 000 populations for all causes combined and leading ten causes in Ethiopia's regions and cities, both sexes, 2019 29

Table S6. Age-standardised death, YLL, YLD, and DALY rates for 100 000 population, both sexes for the leading 11th-20th causes in Ethiopia's regions and chartered cities, 2019 34

Table S7. SDI and LE for both sexes by age-standardised YLL rates for 5 leading causes in Ethiopia's regions and chartered cities, both sexes, 2019..........38

Table S8. Age-standardised death rates per 100 000 population for all causes combined and leading 20 causes in Ethiopia's regions and chartered cities, women and men, 2019 39

Table S9. Age-standardised YLL rates per 100 000 population for all causes combined and leading fifteen causes in Ethiopia's regions and chartered cities, women and men, 2019..........42

Table S10. Age-standardised YLDs rates per 100 000 population for all causes combined and leading fifteen causes in Ethiopia's regions and chartered cities, women, men, 2019..........45

Table S11. Age-standardised DALY rates per 100 000 populations for leading causes in Ethiopia's 9 regions and 2 cities in 1990, 2019 and percentage changes for both sexes 48

Table S12. Age-standardised DALY rates per 100 000 populations for all causes combined and leading fifteen causes in Ethiopia's regions and chartered cities, women, men, 2019..........49

Table S13. Patterns of age-standardised leading causes of premature mortality in Ethiopia and regions and chartered cities, 2019 50

Table S14. Burden of leading risk factors attributable to age-standardised YLL, YLD and DALYs rates in Ethiopia, both sexes, 2019 51

Table S15. Risk factors attributable age-standardised DALY rates per 100 000 population for all risks combined and leading fifteen risk factors, in Ethiopia 9 regions and 2 cities, females, males, and both sexes, 2019 52

Table S16. Leading risk factors attribution to all-cause age-standardised YLLs rate per 100 000 populations for Ethiopia, 9 regions and 2 cities, both sexes, all age, in 2019..........55

Table S17. Top 10 leading causes of national age-standardised death, YLL, YLD and DALY rates per 100,000 for both sexes in Ethiopia in 2019, and the percent change of variation from 1990..........57

Table S18. GATHER Checklist for "Progress in health among regions of Ethiopia, 1990-2019: a sub-national country analysis for the Global Burden of Disease Study 2019"..........59

Section 8: References 61

List of figures and tables

Figures and tables are available in Section 7.

Figures

Figure S1. TFR for regions and chartered cities in Ethiopia, 2019

Figure S2. TFR percent change for regions and chartered cities in Ethiopia, 1990‒2019

Figure S3. Life expectancy between regions and cities, females and males in Ethiopia, in 2019

Figure S4. Variation of life expectancy between regions and cities from 1980 to 2019 by sex, in Ethiopia

Figure S5. LE percent change by regions and chartered cities in Ethiopia, 1990-2019

Figure S6. Ranks of causes of death with age-standardised death rate per 100,000 for both sexes, in Ethiopia, 2019

Figure S7. Trends in YLL rates per 100 000 population for the seven causes with the highest national burden and LE improvement in Ethiopia and regions and chartered cities, 1990–2019

Tables

Table S1. SDI variance between 9 regions and 2 chartered cities in Ethiopia, 1990-2019

Table S2. LE for regions and chartered cities, both sexes, females and males in Ethiopia, 1990 to 2019

Table S3. LE and TFR variance and percent change between regions and chartered cities in Ethiopia, 1990-2019

Table S4. Female LE and TFR in regions and chartered cities in Ethiopia, 2019

Table S5. Age-standardised death, YLL, YLD and DALY rates per 100 000 populations for all causes combined and leading ten causes in Ethiopia's regions and cities, both sexes, 2019

Table S6. Age-standardised death, YLL, YLD, and DALY rates for 100 000 population, both sexes for the leading 11th-20th causes in Ethiopia's regions and chartered cities, 2019

Table S7. SDI and LE for both sexes by age-standardised YLL rates for 5 leading causes in Ethiopia's regions and chartered cities, both sexes, 2019

Table S8. Age-standardised death rates per 100 000 population for all causes combined and leading 20 causes in Ethiopia's regions and chartered cities, women and men, 2019

Table S9. Age-standardised YLL rates per 100 000 population for all causes combined and leading fifteen causes in Ethiopia's regions and chartered cities, women and men, 2019

Table S10. Age-standardised YLDs rates per 100 000 population for all causes combined and leading fifteen causes in Ethiopia's regions and chartered cities, women, men, 2019

Table S11. Age-standardised DALY rates per 100 000 populations for leading causes in Ethiopia's 9 regions and 2 cities in 1990, 2019 and percentage changes for both sexes

Table S12. Age-standardised DALY rates per 100 000 populations for all causes combined and leading fifteen causes in Ethiopia's regions and chartered cities, women, men, 2019

Table S13. Patterns of age-standardised leading causes of premature mortality in Ethiopia and regions and chartered cities, 2019

Table S14. Burden of leading risk factors attributable to age-standardised YLL, YLD and DALYs rates in Ethiopia, both sexes, 2019

Table S15. Risk factors attributable age-standardised DALY rates per 100 000 populations for all risks combined and leading fifteen risk factors, in Ethiopia 9 regions and 2 cities, women, men, and both sexes, 2019

Table S16. Leading risk factors attribution to all-cause age-standardised YLLs rate per 100 000 populations for Ethiopia, 9 regions and 2 cities, both sexes, all age, in 2019

Table S17. Top 10 leading causes of national age-standardised death, YLL, YLD and DALY rates per 100,000 for both sexes in Ethiopia in 2019, and the percent change of variation from 1990

Table S18. GATHER Checklist for “Progress in health among regional states of Ethiopia, 1990-2019: a sub-national country analysis for the Global Burden of Disease Study 2019”

Section 1: Ethiopia subnational population and demography mapping over time, 1942-2019

In 1942, Ethiopia was administratively divided into 12 subnational states and there were boundary changes following 1955, 1987 and 1995 constitutional changes.

Table 1: Administrative boundary changes in Ethiopia following constitutional changes from 1942-1995

Build up time-1	location Name	Build up time-2	Location Name	Build up time-3	Location Name	Build up time-4	Location Name, 2019
	Ethiopia		Ethiopia		Ethiopia		Ethiopia
1942	Arsi	1942	Arsi	1942	Arsi	1995	Oromia
1942	Gamo Gofa	1942	Gamo Gofa	1987	North Omo	1995	SNNP
				1987	South Omo	1995	SNNP
1942	Gojam	1942	Gojam	1987	East Gojam	1995	Amhara
				1987	West Gojam	1995	Amhara
				1987	Metekel	1995	B-Gumuz
1942	Begemeder	1942	Begemeder	1987	North Gondar	1995	Amhara
						1995	Tigray
				1987	South Gondar	1995	Amhara
1942	Harerge	1960	Bale	1987	Bale	1995	Oromia
			Harerge	1987	Dire Dawa	1995	Dire Dawa
					Esat Harerge	1995	Oromia
						1995	Harari
					West Harerge	1995	Oromia
					Ogaden (part)	1995	Somali
1942	Ilubabor	1942	Ilubabor	1987	Ilubabor	1995	Oromia
				1987	Gambella	1995	Gambela
1942	Kefa	1942	Kefa	1942	Kefa	1995	SNNP
1942	Shewa	1942	Shewa	1987	North Shewa	1995	Amhara
				1987	South Shewa	1995	SNNP
				1987	East Shewa	1995	Oromia
				1987	West Shewa	1995	Oromia
		1981	Addis Ababa	1981	Addis Ababa	1995	Addis Ababa
1942	Sidamo	1942	Sidamo	1987	Sidamo	1995	SNNP
				1987	Borena	1995	Oromia
1942	Tigray	1942	Tigray	1942	Tigray	1995	Tigray
1942	Wellega	1942	Wellega	1987	Wellega	1995	Oromia
				1987	Assosa	1995	Benishangul-Gumuz
1942	Wello	1942	Wello	1987	North Wello	1995	Amhara
							Tigray
							Afar
				1987	South Wello	1995	Amhara

In 1991, Ethiopia had 13 administrative subnational states. The current 9 Regions and 2 city councils of Ethiopia were established with the 1995 Ethiopia constitution. The boundaries of current regions differ from the former administrative regions. To map the population of regions for Ethiopia in the 2007, 1994, 1984 census (1–3); we used mainly district (called “woreda”) level mapping. The woreda structure was relatively stable over census years over government changes compared to other structures during the political changes between 1980 and 2017. The population is reasonably higher at woreda level. The three census rounds have produced a report at the woreda level**.** The current administrative system consists of the federal government, regions/chartered cities, zones, woredas (“districts”), and kebeles (lowest administrative units). The current government has also given more attention for districts for the decentralized health care system, including woreda based planning, to have decision-making occur near to the community and for resource allocation. Woreda level population mapping is also helpful to investigate the possibility of providing woreda level subnational estimates for Ethiopia in the future. Generally, mapping the

1994 and 2007 population has been easy since the location of data sources did not have geographic boundary changes for subnational states.

Table 2: Administrative subnational regional states, provinces, districts in 1985

Administrative Regions	Awrajas (Province)	Districts
1. Arsi	3	22
2. Bale	5	25
3. Gamo Gofa	4	22
4. Gojam	7	35
5. Gondar	7	29
6. Harerge	15	66
7. Ilubabor	5	34
8. Kefa	6	38
9. Shewa	11	93
10. Sidamo	6	34
11. Tigray	8	55
12. Wellega	9	49
13. Wello	12	37
Total	98	539

Source: Ministry of interior 1977 E.C (1985)

Reference: National Atlas of Ethiopia (CSA Library)

1.1 Afar region

Afar region did not exist in the 1984 census. Districts from the then Wello and Hararge regions jointly form the current Afar region. The districts' included were; DUBTI woreda (Wello), ELIDAR woreda (Wello), AWSSA woreda (Wello), AFAMBO woreda (Wello), MILE woreda (Wello), ARTUMA woreda (Wello), MISRAK AWASH woreda (Hararge), GEWANE woreda (Hararge). The population for these districts have been subtracted from the current Amhara and Oromia regions of their 1984 population as the then Wello Region has been included in Amhara and Hararge region included in Oromia. The 2007 and 1994 population by age and sex is available on the report for Afar region.

1.2 Amhara region

Amhara region was formed combining the former Gondar, Gojjam, Shewa and Wello regions. Some districts from Gondar moved to the current Tigray regional state, some districts from Gojjam moved to the current Benishangul-Gumuz region, some from the former Shewa moved to the current Oromia and SNNP region and from Wello moved to the current Tigray and Afar regions. The 2007 and 1994 population by age and sex is available on the report for Amhara region.

We used the following formula to estimate total population of the current Amhara region for the 1984 Amhara region:

Amhara Region$_{agesex\ 1984}$= (Gondar$_{agesex1984}$ – Woredas to Tigray$_{agesex1984}$) + (Gojjam$_{agesex1984}$ – Woredas to BG $_{agesex1984}$) + (Shewa$_{agesex1984}$ – Woredas to SNNP$_{agesex1984}$ – Woreda to Oromia$_{agesex1984}$) + (Wello$_{agesex1984}$ – Woreda to Tigray$_{agesex1984}$ – Woreda to Afar$_{agesex1984}$)

where, Amhara Region$_{agesex\ 1984}$ is the current Amhara *region population for both sexes in 1984,* Gondar$_{agesex1984}$ is the then *Gondar's region population for both sexes in 1984,* Woredas to Tigray$_{agesex1984}$ *is woreda population for both sex classified to Tigray region of the 1984,* Gojjam$_{agesex1984}$ is the then Gojjam's *region population for both sexes in 1984,* Woredas to BG agesex1984 *is woreda population for both sex in 1984 classified to the current Benishangul Gumuz ,* Shewa$_{agesex1984}$ *is* the then Shewa *region population for both sexes in 1984,* Woredas to SNNP$_{agesex1984}$ *is woreda population for both sexes in 1984 classified to the current SNNP region,* Woreda to Oromia$_{agesex1984}$ *is woreda population for both sexes in 1984 classified to the current Oromia region,* Woreda to Somali$_{agesex1984}$ *is woreda population for both sexes in 1984 classified to the current Somali region,* Wello$_{agesex1984}$ *is* the then Wello *region population for both sexes in 1984,,* Woreda to Tigray$_{agesex1984}$ *woreda population for both sex in 1984 classified to the current* Tigray, Woreda to Afar$_{agesex1984}$ is *woreda population for both sex in 1984 classified to the current Afar.*

1.3 Oromia region

Many districts were further divided between 1994 (185 woreda) and 2007 census (280 woreda) and one woreda was included from the Somali region (CHINAKSAN district). The 2007 and 1994 population by age and sex is available on the report for Oromia region.

Oromia Region$_{agesex\ 1984}$ Population= (Wellega $_{agesex1984}$ – Woredas to BENISHANGUL-GUMUZ $_{agesex1984}$) + (Shewa $_{agesex1984}$ - Woreda to SNNP$_{agesex1984}$) – (Woreda to Amhara$_{agesex1984}$)) + ((Hararge $_{agesex1984}$ - Woreda to Afar$_{agesex1984}$ - Woreda to Harari$_{agesex1984}$ - Woreda to Somali$_{agesex1984}$ - Woreda to Dire Dawa $_{agesex1984}$)) + (ILLUBABOUR$_{agesex1984}$ – Woredas to Gambella$_{agesex1984}$ – woredas to *SNNP* $_{agesex1984}$) + (Bale$_{agesex1984}$ –Woredas to Somalia$_{gesex1984}$) + (Keffa $_{agesex1984}$ - *woredas to SNNP*$_{agesex1984}$)+ (Arssi $_{agesex1984}$) + (Sidamo $_{agesex1984-}$ *Woredas to SNNP*$_{agesex1984}$)

where, Oromia Region$_{agesex\ 1984}$ is *Oromia region population for both sexes in 1984,* Wellega $_{agesex1984}$ is the then *Wellega's region population for both sexes in 1984,* Woredas to BENISHANGUL-GUMUZ $_{agesex1984}$ *is is woreda population for both sex classified to Benishanguk-Gumuz region of the 1984,* Shewa $_{agesex1984}$ is the then Shewa's *region population for both sexes in 1984,* Woreda to SNNP$_{agesex1984}$ *is woreda population for both sex in 1984 classified to the current SNNP region ,* Woreda to Amhara$_{agesex1984}$ *is woreda population for both sexes in 1984 classified to the current Amhara region,* Hararge $_{agesex1984}$ *is* the then Hararge *region population for both sexes in 1984,* Woreda to Afar$_{agesex1984}$ *is woreda population for both sexes in 1984 classified to the current Afar region,* Woreda to Harari$_{agesex1984}$ *is woreda population for both sexes in 1984 classified to the current Harari region,* Woreda to Somali$_{agesex1984}$ *is woreda population for both sexes in 1984 classified to the current Somali region,* Woreda to Dire Dawa $_{agesex1984}$ *is woreda population for both sexes in 1984 classified to the current Dire Dawa city ,* ILLUBABOUR$_{agesex1984}$ *is* the then Illubabour*'s region population for both sexes in 1984,* Woredas to Gambella$_{agesex1984}$ *is woreda population for both sexes in 1984 classified to the current Gambella region,,* woredas to *SNNP* $_{agesex1984}$ *is woreda population for both sexes in 1984 classified to the current SNNP region,,* Bale$_{agesex1984}$ is the then *Bale's region population for both sexes in 1984,* Woredas to Somalia$_{gesex1984}$ *is woreda population for both sex in 1984 classified to the current Somali region,* Keffa $_{agesex1984}$ is the then *Keffa's region population for both sexes in 1984,* *woredas to SNNP*$_{agesex1984}$ *is woreda population for both sexes in 1984 classified to the current SNNP region,,* Arssi $_{agesex1984}$ *is* the then Arssi*'s region population for both sexes in 1984,* Sidamo $_{agesex1984}$ *is* the then Sidamo*'s region population for both sexes in 1984* ***and*** *Woredas to SNNP*$_{agesex1984}$ *is woreda population for both sexes in 1984 classified to the current SNNP region*

1.4 Southern Nations and Nationalities and People region

Southern Nations and Nationalities and People (SNNP) did not exist in the 1984 census. It was formed joining woredas from the former Shewa, Illubabour, Keffa, Gamo Gofa and Sidamo regions. The 2007 and 1994 population by age and sex is available on the report for SNNP region.

We used the following formula to conceptualize and estimate the 1984 population for the current SNNP Region.

SNNP $_{agesex1984}$ = ((Shewa $_{agesex1984}$ - Woreda to Oromia$_{agesex1984}$) – (Woreda to Amhara$_{agesex1984}$)) + ((ILLUBABOUR$_{agesex1984}$ – Woredas to Gambella$_{agesex1984}$ – (Woredas to *Oromia* $_{agesex1984}$)) + (Keffa $_{agesex1984}$ - *Woredas to Oromia* $_{agesex1984}$) + (Gamo Gofa$_{agesex1984}$) + (Sidamo $_{agesex1984}$ - *Woredas to Oromia* $_{agesex1984}$)

where, SNNP $_{agesex1984}$ *is SNNP region population for both sexes in 1984,* Shewa $_{agesex1984}$ *is Shewa region population for both sexes in 1984,* Woreda to Oromia$_{agesex1984}$ *is woreda population for both sex classified to Oromia region of the 1984,* Woreda to Amhara$_{agesex1984}$ *is woreda population for both sex classified to Amhara region,* ILLUBABOUR$_{agesex1984}$ *is Illubabour region population for both sexes in 1984,* Woredas to Gambella$_{agesex1984}$ *woreda population for both sex classified to Gambella region of the 1984,* Keffa $_{agesex1984}$ *is Keffa region population for both sexes in 1984,* Gamo Gofa$_{agesex1984n}$*is Gamo Gofa region population for both sexes in 1984,* Sidamo $_{agesex1984}$ *Sidamo region population for both sexes in 1984.*

1.5 Gambella region

Gambella region did not exist in 1984 census. Woredas from the then ILLUBABOUR were separated to form the current Gambella. The woredas' included were; GAMBELLA woreda, ABOBO woreda, GOG JOR woreda, JIKAWO woreda, AKOBO woreda, GODARE woreda, ITANG woreda. These woredas were subtracted from the current Oromia region with their respective 1984 population. The 2007 and 1994 population by age and sex is available on the report for Gambella region.

1.6 Somali region

Somali region did not exist in 1984 census. Woredas from the then HARARGE region and BALE region were considered to form the current Somali region. The woredas' included from the former HARARGE region were; AYISHA woreda, DENBEL woreda, SHINILE woreda, ERER woreda, DEGAHABUR AWRAJA, GODIE AWRAJA, KEBRI DEHAR AWRAJA, KELAFO AWRAJA, WELWEL AND WARDER AWRAJA, AFDEM woreda, BABILE woreda, GURSUM woreda, JIJIGA woreda, AWUBERE woreda, KEBRI BEYAH woreda, HARSHIN-woreda, FIK woreda, HAMERO woreda, MEYU MULUKE woreda; from BALE region were; LEGEHIDA woreda and GURADAMOLE woreda. Some of the locations included were Awaja (province, the structure which had many woredas under it) from the former HARARGE region as there were no woreda level reports. These woreda populations were subtracted from the current Oromia and Amhara region with their respective 1984 population. The 2007 and 1994 population by age and sex is available on the report for Somali region.

1.7 Benishangul-Gumuz region

This region did not exist in the 1984 census. It was formed joining some woreda from the former Gojjam Region (DANGUR woreda, GUBA woreda, WENBERA woreda, MANDURA woreda, DIBATE woreda, GUANGUA woreda) and Wellega region (KURMUK woreda, ASOSA woreda, GIZEN woreda and BIEGI woreda). Population estimation to Benishangul Gumuz region to 1984 was relatively easy. The 2007 and 1994 population by age and sex is available on the report for Benishangul-Gumuz region.

Total population of the current Benishangul-Gumuz in 1984 was

$$Total\ Population = DANGUR_{agesex1984} + GUBA_{agesex1984} + WENBERA_{agesex1984} + MANDURA_{agesex1984} + DIBATE_{agesex1984} + GUANGUA_{agesex1984} + KURMUK_{agesex1984} + ASOSA_{agesex1984} + GIZEN_{agesex1984} + BIEGI_{agesex1984}$$

where, $DANGUR_{agesex1984}$ *is Dangbur woreda population for both sexes in 1984,* $GUBA_{agesex1984}$ *is Guba woreda population for both sexes in 1984,* $WENBERA_{agesex1984}$ *is Wenbera woreda population for both sexes in 1984,* $MANDURA_{agesex1984}$ *is Mandura woreda population for both sexes in 1984,* $DIBATE_{agesex1984}$ *is Dibate woreda population for both sexes in 1984,* $GUANGUA_{agesex1984}$ *is Guangua woreda population for both sexes in 1984,* $KURMUK_{agesex1984}$ *is Kurmuk woreda population for both sexes in 1984,* $ASOSA_{agesex1984}$ *is Asosa woreda population for both sexes in 1984,* $GIZEN_{agesex1984}$ *is Gizen woreda population for both sexes in 1984,* $BIEGI_{agesex1984}$ *is Biegi woreda population for both sexes in 1984.*

These woreda populations were subtracted from the current Amhara and Oromia regions with their 1984 population

1.8 Harari region

Harari region did not exist in 1984 census. Harar town and Harar rural kebeles separated from Hararge region to form the current Harari region. Relatively it has been easy to estimate the 1984 population. The 2007 and 1994 population by age and sex is available on the report for Harari subnational regional state. These woreda populations were subtracted from the current Oromia region of its 1984 population

1.9 Addis Ababa chartered city

Addis Ababa was city administration in the 2007, 1994 and 1984 census rounds and we considered reported population.

1.10 Dire Dawa chartered city

Dire Dawa chartered city did not exist in 1984 census. Woredas from the then HARARGE region, were considered to form the current Dire Dawa. The Woredas' included from the former HARARGE region were; Dire Dawa town and Gorgora woreda. The 2007 and 1994 population by age and sex is available on the report for Dire Dawa region. These woreda population were subtracted from the current Oromia region of its respective 1984 population

In terms of data sources, GBD 2019 has used 1057 distinct data sources by location for Ethiopia, regions and chartered cities.

1.11 Tigray region

According to census 1984 report of CSA (hard copies at CSA libraries which need to be digitalize); Tigray region was administratively divided into 8 Awrajas (provinces), 56 districts and 58 urban centers. However due to security and other reasons the census covered only 18 urban centers. The 1984 census has not covered the entire rural population of the region which is estimated to be 2,174,894 in 1984. In this case, we have not used woreda approach to map the population. Population data was not presented by sex and age for provinces. We used a different approach for Tigray

subnational state to take the whole population of the then Tigray Region for 1984 and add population of woreda or districts which were added in the 1994 census round. These were woreda which were under the then Gondar Region; TELEMET woreda (Population=60668), WELKAYIT woreda (Population=66415), TSEGEDE woreda (Population= 69000), SETIT woreda (Population=36083); woreda which were under the then Wello Region; ALAMATA woreda (Population=50731), OFLA woreda (Population=110994). These woreda were included to Tigray region in the 1994 and 2007 census. To map the population, we map the population of woreda using the former Gondar and Wollo region population age and sex structure (See the steps below). Then the population of these districts subtracted from the current Amhara region (currently involving the then Gondar and Wello Regions). The 2007 and 1994 population by age and sex is available on the report for Tigray region.

Table 3: Number of data sources by location in Ethiopia, GBD 2019

**Location**	**Numbers of distinct data sources by location**
Ethiopia	313
Addis Ababa	107
Afar	57
Amhara	75
Benishangul-Gumuz	54
Dire Dawa	51
Gambella	72
Harari	52
Oromia	80
Somali	57
SNNP	79
Tigray	60
Total	*1057*

Section 2: Global Burden of Disease (GBD 2019) Method Summary

The details of GBD demography methodology is published elsewhere (4), but in brief GBD estimates fertility, mortality and population using diversity of data sources. Spatiotemporal Gaussian process regression (ST-GPR) was used to generate age-specific fertility rates for 5-year age groups between ages 15 and 49 years, to synthesise data sources after correction for known biases. Adult mortality was measured as the probability of death between ages 15 and 60 years. HIV-free life tables were then estimated using estimates of under-5 and adult mortality rates using a relational model life table system created for GBD. Independent estimates of HIV-specific mortality generated by an epidemiological analysis of HIV prevalence surveys and antenatal clinic sero-surveillance and other sources were incorporated into the estimates in countries with large epidemics. Annual and single-year age estimates of net migration and population for each country and territory were generated using a Bayesian hierarchical cohort component model that analysed estimated age-specific fertility and mortality rates. GBD 2019 estimated each epidemiological quantity of interest for 23 age groups, males, females and both sexes combined for 204 countries and territories from 1990 to 2019 (5). All metrics were estimated separately for Ethiopia's 9 regions and 2 chartered cities, and are presented with their 95% uncertainty intervals (UIs). All estimates produced for GBD report 95% uncertainty intervals (UIs) that account for sampling and non-sampling error associated with data and various assumptions of the modelling process and are derived from the 2·5th and 97·5th percentiles of 1000 draws (6). The GBD disease and injury analytical framework generated estimates for every year from 1990 to 2019. GBD diseases and injuries were organised into a levelled cause hierarchy from the three broadest causes of death and disability at Level 1 to the most specific causes at Level 4. Within the three Level 1 causes (communicable, maternal, neonatal, and nutritional diseases; non-communicable diseases; and injuries), there were 174 Level 3 causes (7).

We estimated all-cause mortality rates for each age-sex-location-year using multistage models of adult and under-5 mortalities and the GBD model life table system (8). Cause-specific mortality rates were computed using the GBD cause-of-death database and, in most cases, the cause of death ensemble model (CODEm) as previously described (9). We estimated years of life lost (YLLs) for each cause by location, age, sex, and year by multiplying each cause specific death by the normative standard life expectancy and each age (10). The YLLs are years lost due to premature mortality and calculated by subtracting the age at death from the longest possible life expectancy for a person at that age. We estimated YLDs by multiplying the prevalence of each cause and its consequences by its disability weight, developed using population-based surveys, and corrected for comorbidity. Years lived with disability is measured by taking the prevalence of the condition multiplied by the disability weight for that condition (7). Disability weights are measured on a scale from 0 to 1, where 0 equals a state of full health and 1 equals death. Disability weights for the 440 health states (including combined health states) have been used to estimate YLDs for the GBD 2019 study and available online here: http://ghdx.healthdata.org/record/ihme-data/gbd-2019-disability-weights.

To estimate YLDs, a Bayesian meta-regression tool, DisMod-MR 2.1 published elsewhere in details (5), was used to evaluate all available data whilst ensuring consistency between incidence, prevalence, remission, and cause of death rates for each condition. The calculations for life expectancy at birth (LE) and maximum life expectancy at each age have been previously reported (8). Healthy life expectancy (HALE) summarizes overall population health accounting for length of life and level of health loss by age using YLD estimates and the GBD life tables, as previously described (4). Disability-adjusted life-years (DALYs), a combined measure of health lost to fatal and non-fatal causes, are calculated as the sum of YLLs and YLDs for each age-sex-location-year. One DALY equals one lost year of healthy life. We created maps of Ethiopia for the changes in all-cause, age-standardized YLLs and YLDs over time using Ethiopia census boundary data and geographic information software (11,12). GBD 2019 estimated Socio-demographic Index (SDI), it is a composite indicator of development status strongly correlated with health outcomes. It is the geometric mean of 0 to 1 indices of total fertility rate under the age of 25, mean education for those ages 15 and older, and lag distributed income (LDI) per capita. As a composite, a location with an SDI of 0 would have a theoretical minimum level of development relevant to health, while a location with an SDI of 1 would have a theoretical maximum level (8).

In the risk factor estimation, relative risk of mortality and morbidity, exposure to each risk factor, and ultimately attributable deaths or DALYs were estimated for each risk-outcome pair. The GBD 2019 estimation of attributable burden followed the general framework established for comparative risk assessment (CRA). GBD estimated the attributable burden for each risk by multiplying the YLLs and YLDs for each outcome of interest by the population attributable fraction (PAF) for the risk–outcome pair. The PAF represents the proportion of DALYs that would have been avoided in a given year if the exposure to a risk factor in the past had been at the theoretical minimum risk exposure level (13). The GBD risk factors were organised into a levelled hierarchy: Level 1 risk factors are behavioural; environmental and occupational; and metabolic; Level 2 risk factors include 20 clusters of risks; Level 3 includes 52 clusters of risks; and Level 4 includes 69 specific risk factors (14).

Section 3: Input Data Sources

The EPHI, in collaboration with IHME, gathered 1057 distinct data sources by location for Ethiopia and all regions and cities that included census, demographic surveillance, household surveys, disease registry, health service utilisation, disease notification, and other data for this analysis (appendix section 1). A comprehensive description of data input sources (for each causes of death, covariates, fertility, mortality, non-fetal health outcomes, population and risk factors) and data quality of sources (data collection methods, completeness by age/sex and year, sample size and representativeness, and standard errors for the potential biases) and modelling for GBD 2019 has been reported here: http://ghdx.healthdata.org/gbd-2019/data-input-sources.

Section 4: GBD Strengths and Limitations

The relevant limitations for burden of disease estimation in Ethiopia focus on data source availability and accessibility. When data were not available for a particular location, GBD modelled estimates use data from other locations and predictive covariates (5). Causes of death data sources used in GBD 2019 were mainly from verbal autopsy and sibling history, whose limitations have been discussed elsewhere (5,9). When new data or changes in modelling lead to

changes in estimates of disease burden, a strength of the GBD approach is that all previous estimates are recalculated with the newest model (4). Regardless of such rigorous and standardised methodology in GBD, the data incorporated into GBD 2019 were very scarce for Ethiopia and its regions and cities. The collaboration between EPHI and IHME helped to mitigate these limitations. The initiative has helped EPHI to transform the way health data is processed and used for health decisions at different levels. EPHI has built local data analytic capacity and platforms (https://vizhub.ephi.gov.et/) and created an in-country data repository system to archive, access, and share data (https://rtds.ephi.gov.et/public/). A technical team from EPHI and IHME mapped and did inventory on priority data sources for different diseases and conditions, and accessed priority data types. The analysis has considered political, government and administrative changes over the years to map available data and populations. EPHI and IHME have organized one-week subnational review workshop and reviewed GBD 2019 subnational preliminary results with local researchers and collaborator experts in Ethiopia. Finally, a technical team from IHME and EPHI has reviewed final subnational results and agreed on the findings.

In GBD 2019, because of institutional collaboration, we used verbal autopsy data from seven Health and Demographic Surveillance Systems which represent Amhara, Oromia, SNNP, and Tigray regions. The data has been relatively comprehensive by age, sex, cause of death and location compared to scientific literatures. This resulted in including some data sources in the detail GBD causes. Because of the lack of completeness, misclassification of causes of death, unknown causes of death of these sources, there were wider uncertainties, as indicated with the 95% UI for age-standardised rates and therefore some of the variation could be due to different data sources rather than true underlying variation which largely affect policy debates, prioritization of causes, and health decisions.

The team overcame challenges of GBD regional level analysis to generate reliable estimates though institutional collaboration involving more than 700 GBD collaborator experts from Ethiopia. The estimates benefited from rounds of technical reviews and subnational review workshop having multidisciplinary researchers from EPHI, Ministry of Health, Central Statistical Agency, local universities, research institutes and non-governmental organizations in Ethiopia. The collaboration has helped to strengthen local data system in the National Data Management Center (NDMC) for health at EPHI. To improve estimates further, the collaboration has created sustainable system through building local burden of disease, evidence to policy translation and data repository and governance units at NDMC.

Section 5: Statement of GATHER Compliance

This study complies with the Guidelines for Accurate and Transparent Health Estimates Reporting (GATHER) recommendations (5). GATHER defines best practices for documenting studies that synthesize evidence from multiple sources to quantitatively describe past and current population health and its determinants. These practices include documenting and sharing data inputs, analyses and methods, and results. Documenting the input data on which estimates are based, and the methods by which estimates are derived, is essential for the accurate interpretation and use of results. GBD 2019 analyses were completed with Python version 3.6.2, Stata version 13, and R version 3.5.0. Statistical code used for GBD estimation is publicly available online: http://ghdx.healthdata.org/gbd2019/code. See table S18 for the GATHER checklist.

The GATHER recommendations can be found at the GATHER website under GATHER Statement.

Section 6: Author Contributions

This section will be completed following re-submission of the manuscript.

Section 7: Figures and Tables

Figures

Figure S1. TFR for regions and chartered cities in Ethiopia, 2019

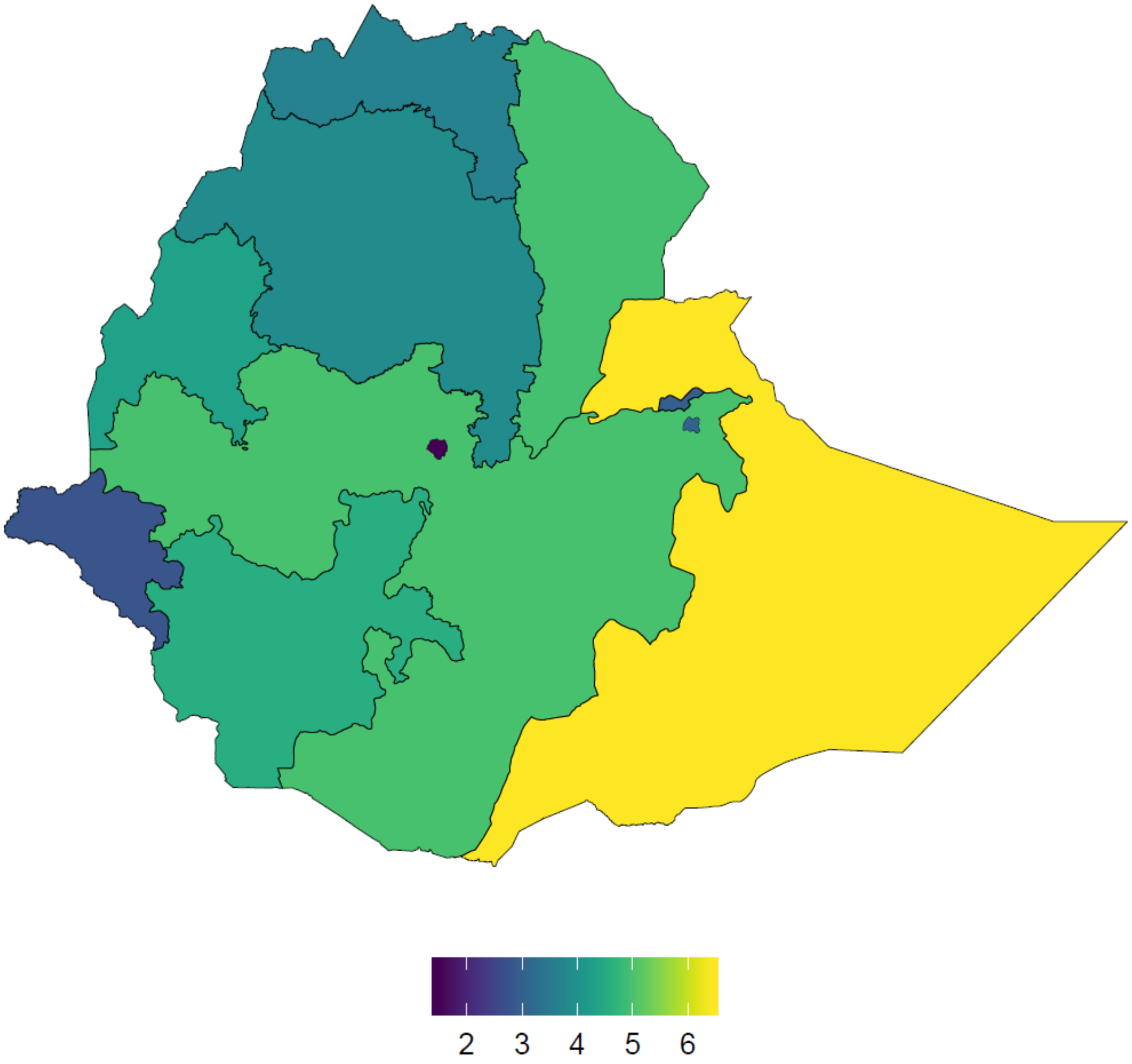

Figure S2. TFR percent change for regions and chartered cities in Ethiopia, 1990−2019

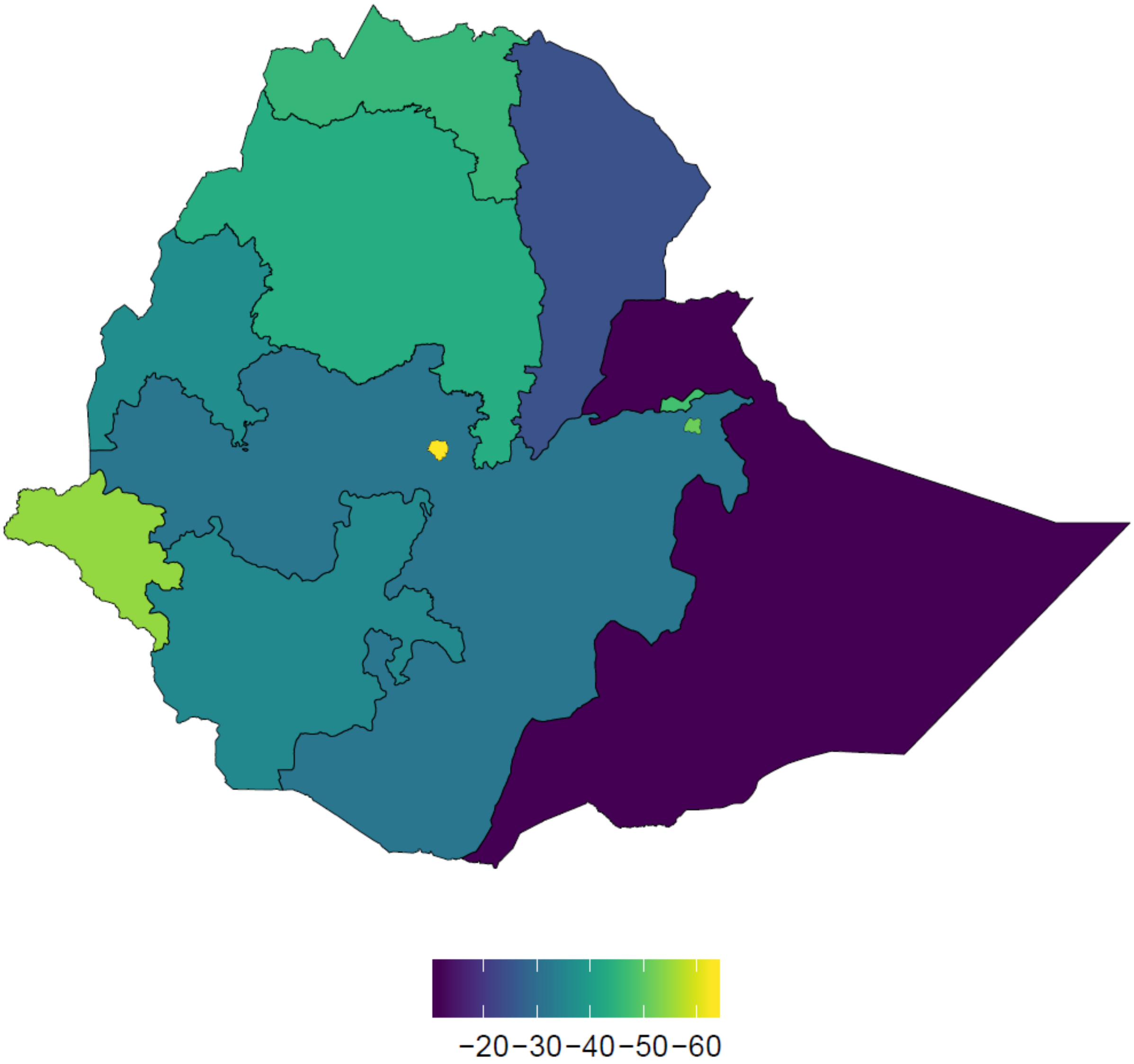

Figure S3. Life expectancy between regions and cities, females and males in Ethiopia, in 2019

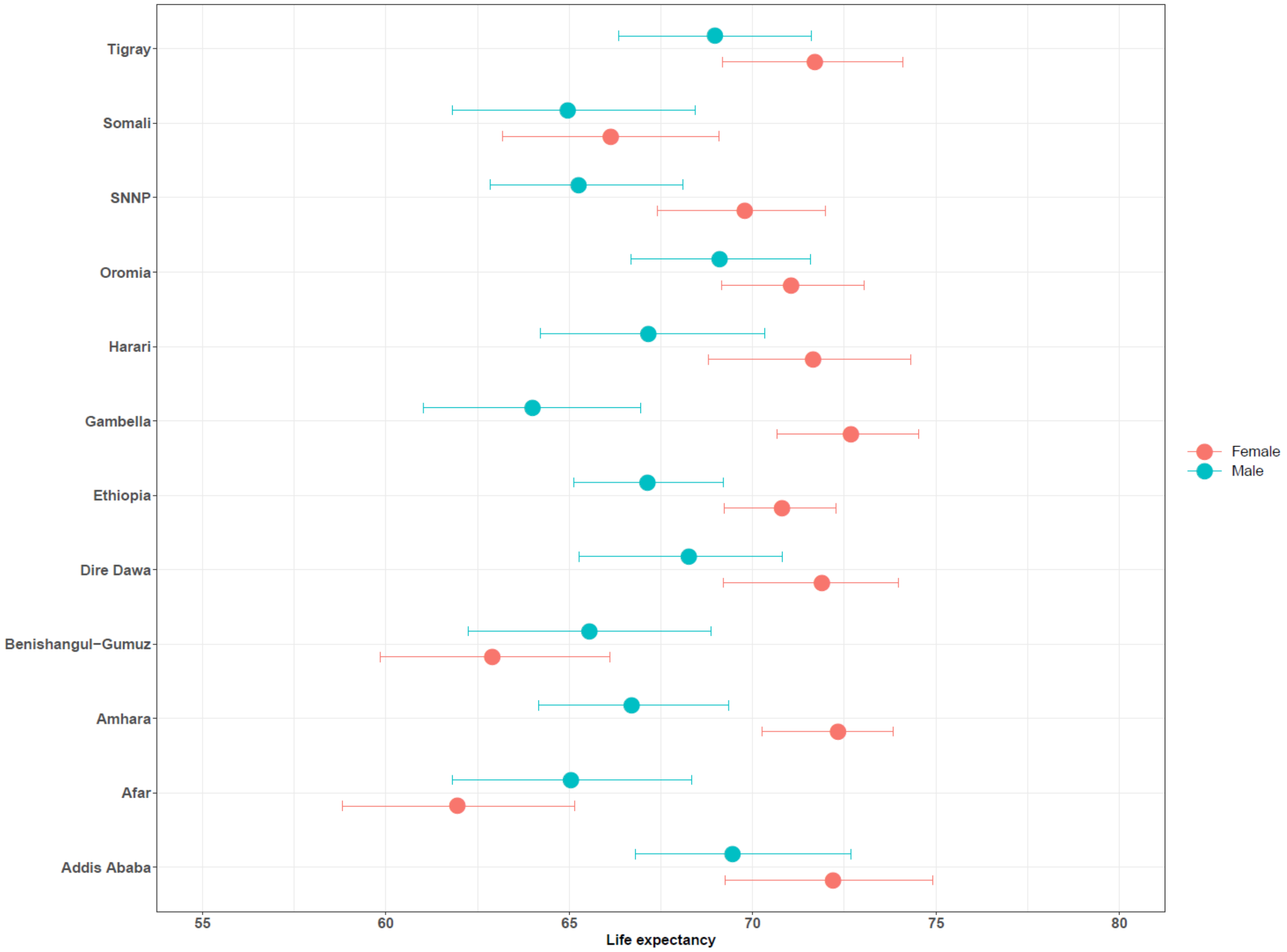

Figure S4. Variation of life expectancy between regions and cities from 1980 to 2019 by sex, in Ethiopia

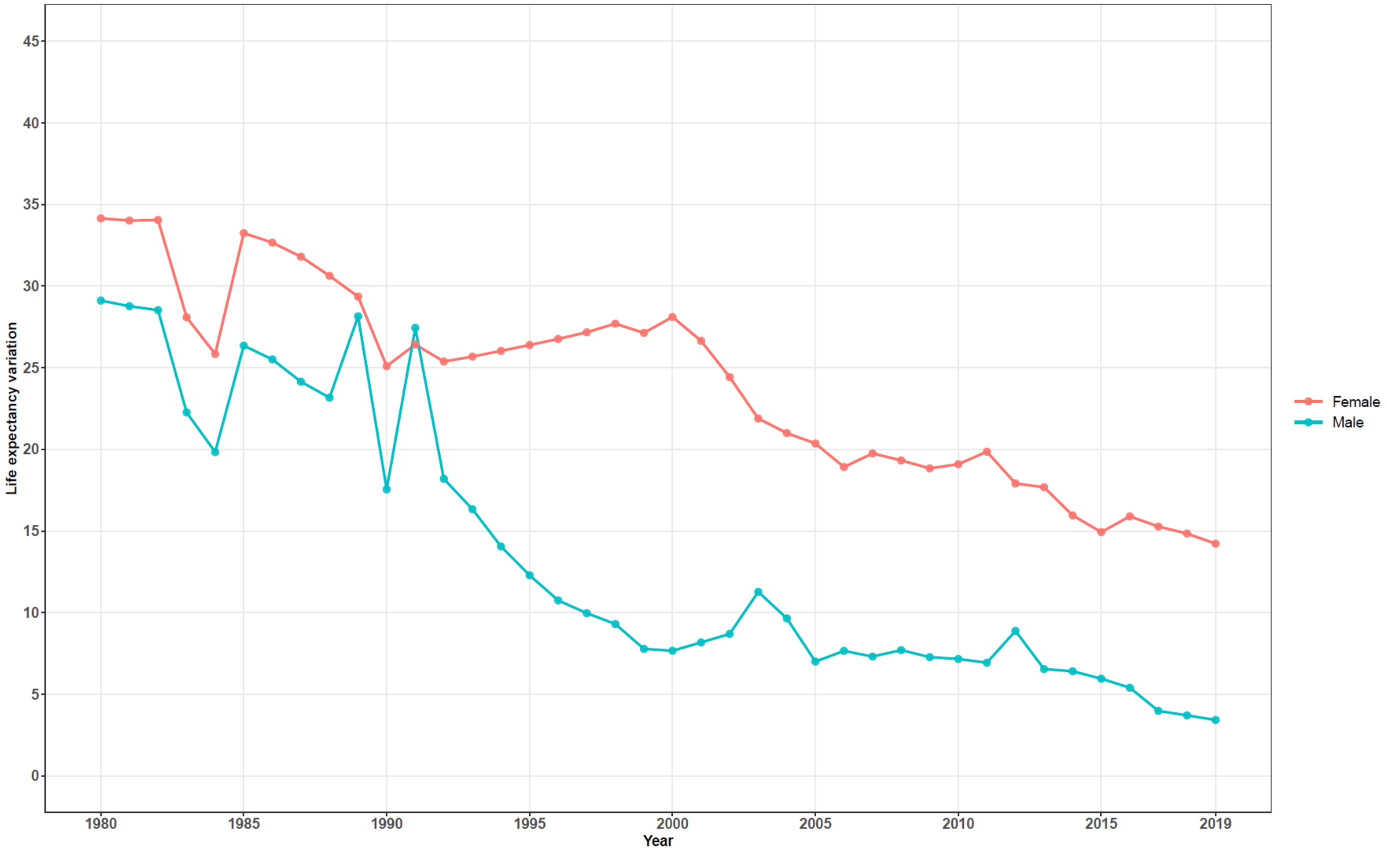

Figure S5. LE percent change by regions and chartered cities in Ethiopia, 1990-2019

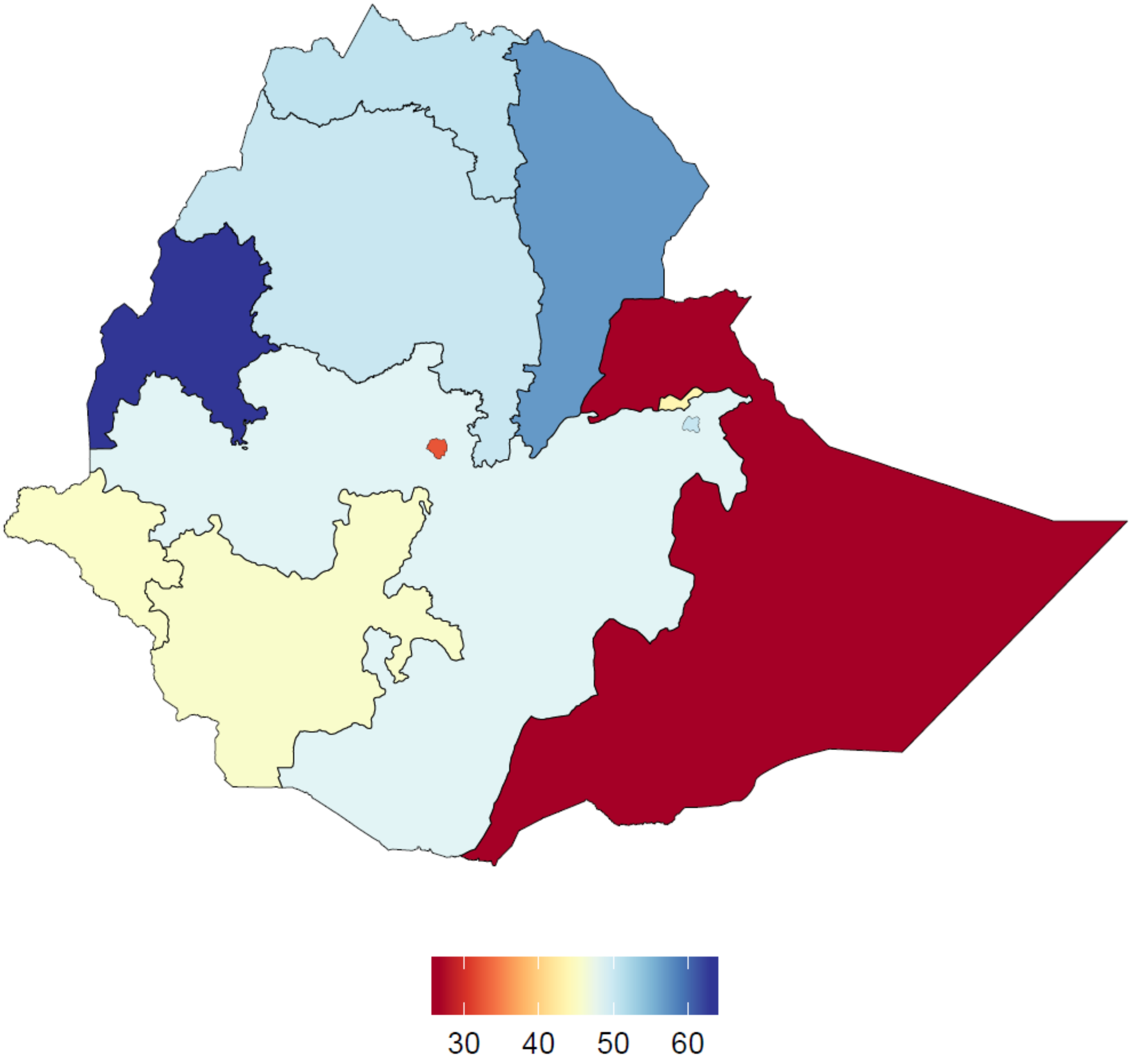

Figure S6. Ranks of causes of death with age-standardised death rate per 100,000 for both sexes, in Ethiopia, 2019

Rank	Causes of death, 1990	Death rate/100,000
1	Tuberculosis	317.34(262.65-381.28)
2	Diarrheal diseases	305.77(146.2-483.73)
3	Lower respiratory infections	223.01(184.74-264.35)
4	Stroke	151.89(118.73-182.4)
5	Ischemic heart disease	110.26(84.68-140.93)
6	War and conflict	100.47(100.47-100.47)
7	Protein-energy malnutrition	83.04(55.79-117.63)
8	Neonatal disorders	78.42(69-88.94)
9	Cirrhosis and other CLDs	77.13(46.75-107.53)
10	Measles	64.19(22.38-130.62)
11	Hypertensive heart disease	58.65(35.01-88.42)
12	Diabetes mellitus	57.91(47.8-73.1)
13	Meningitis	50.24(42.31-59.68)
14	COPD	46.39(36.4-56.4)
15	Chronic kidney disease	39.59(32.49-46.12)
16	Maternal disorders	39.55(30.63-52.4)
17	Road injuries	31.71(25.46-38.69)
18	HIV/AIDS	31.22(17.32-61.22)
19	Alzheimer's disease	23.16(5.28-66.28)
20	Asthma	22.71(15.51-31.92)
21	Self-harm	21.67(16.78-26.42)
24	Interpersonal violence	15.59(12.4-19.89)
33	Falls	12.19(9.12-15.35)
35	Breast cancer	10.71(8.28-13.83)
44	Malaria	7.34(1.07-23.73)

Rank	Causes of death, 2019	Death rate/100,000
1	Stroke	90.18(70.62-110.04)
2	Lower respiratory infections	86.44(75.36-97.65)
3	Ischemic heart disease	84.24(62.56-105.75)
4	Diarrheal diseases	76.45(45.12-112.15)
5	Tuberculosis	60.9(50.44-71.5)
6	Cirrhosis and other CLD	52.18(44.17-62.07)
7	Neonatal disorders	44.31(34.92-56.99)
8	Diabetes mellitus	36(31.01-41.43)
9	HIV/AIDS	33.56(28.68-39.58)
10	Hypertensive heart disease	31.97(17.85-53.09)
11	COPD	28.32(22.68-33.35)
12	Alzheimer's disease	27.82(6.68-75.12)
13	Chronic kidney disease	26.17(22.48-30.08)
14	Road injuries	14.51(12.16-17.36)
15	Meningitis	14.14(12-16.55)
16	Protein-energy malnutrition	12.9(9.77-16.7)
17	Self-harm	10.13(8.21-12.9)
18	Breast cancer	9.71(7.96-11.64)
19	Interpersonal violence	9.68(7.94-11.89)
20	Falls	9.6(8.09-11.29)
21	Malaria	9.12(1.77-24.43)
23	Asthma	8.54(6.21-14.61)
25	Maternal disorders	8.16(5.96-11.04)
68	Measles	1.45(0.44-3.48)
110	war and conflict	0.15(0.14-0.17)

Communicable, maternal, neonatal and nutritional diseases
Non communicable diseases
Injuries

Figure S7. Trends in YLL rates per 100 000 population for the seven causes with the highest national burden and LE improvement in Ethiopia and regions and chartered cities, 1990–2019
Panel A: HIV/AIDS. Panel B: Tuberculosis. Panel C: Diarrhoeal diseases. Panel D: Lower respiratory infections. Panel E: Measles. Panel F: Protein-energy malnutrition. Panel G: Maternal disorders.

Panel A:

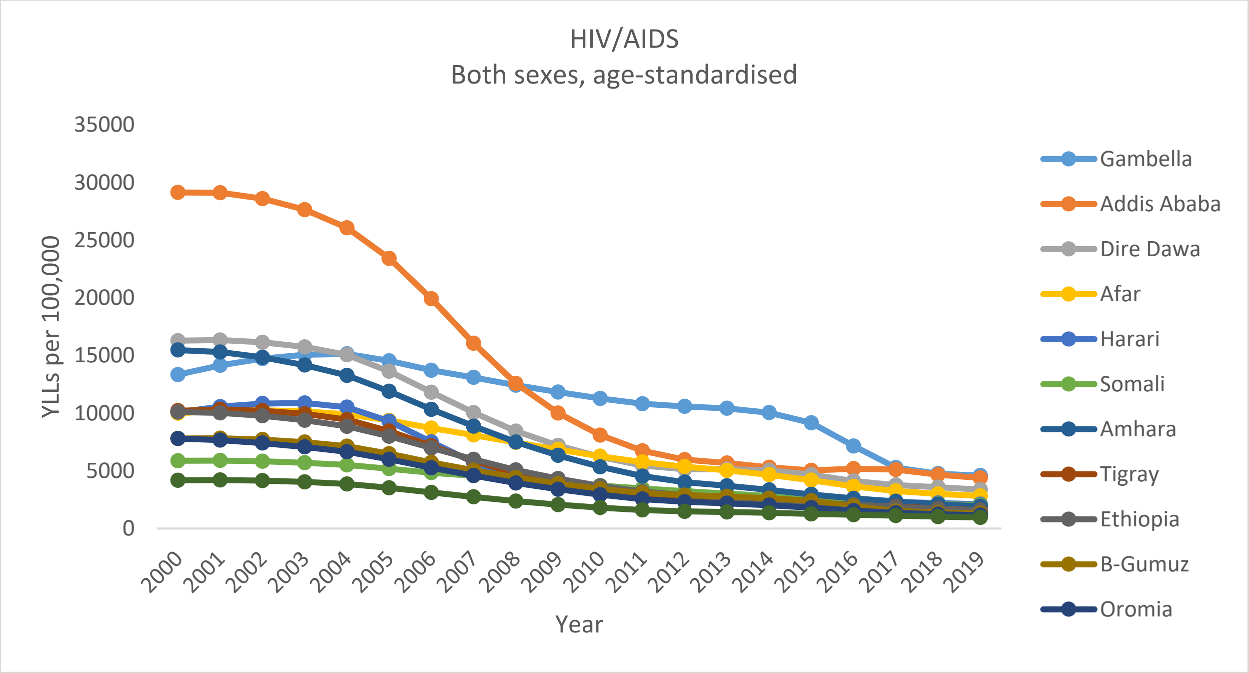

Panel B:

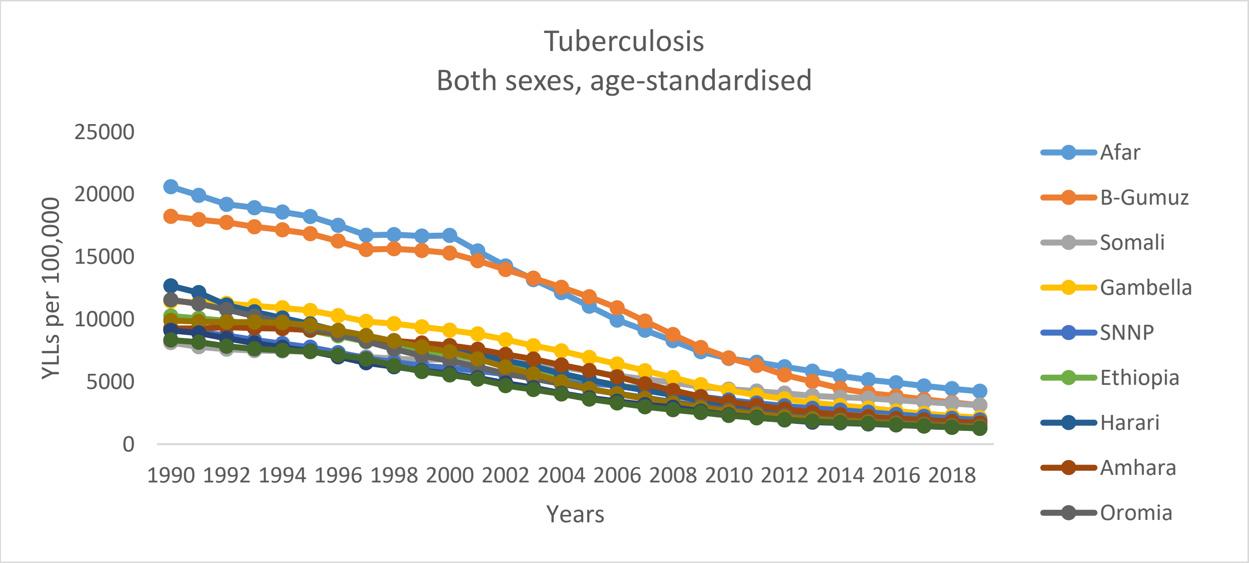

Panel C:

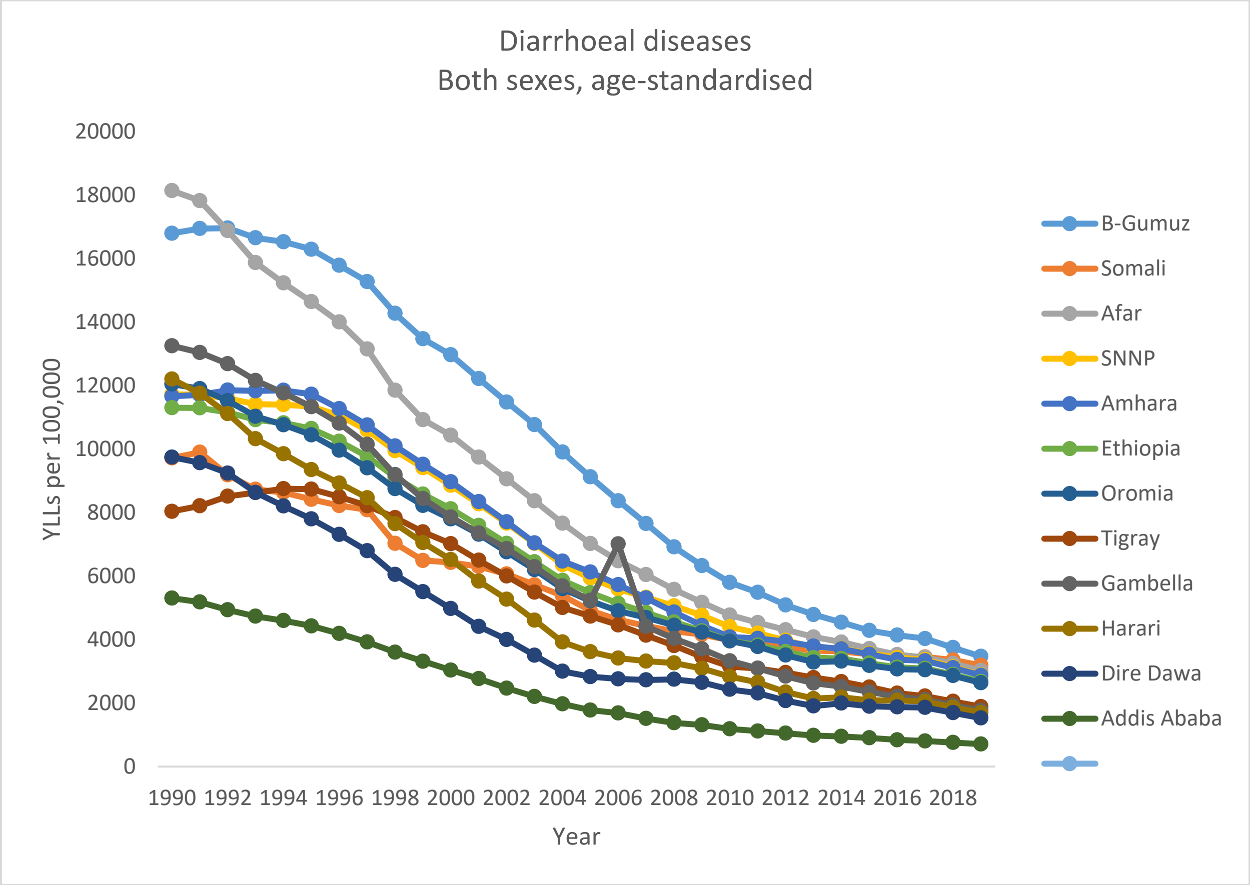

Panel D:

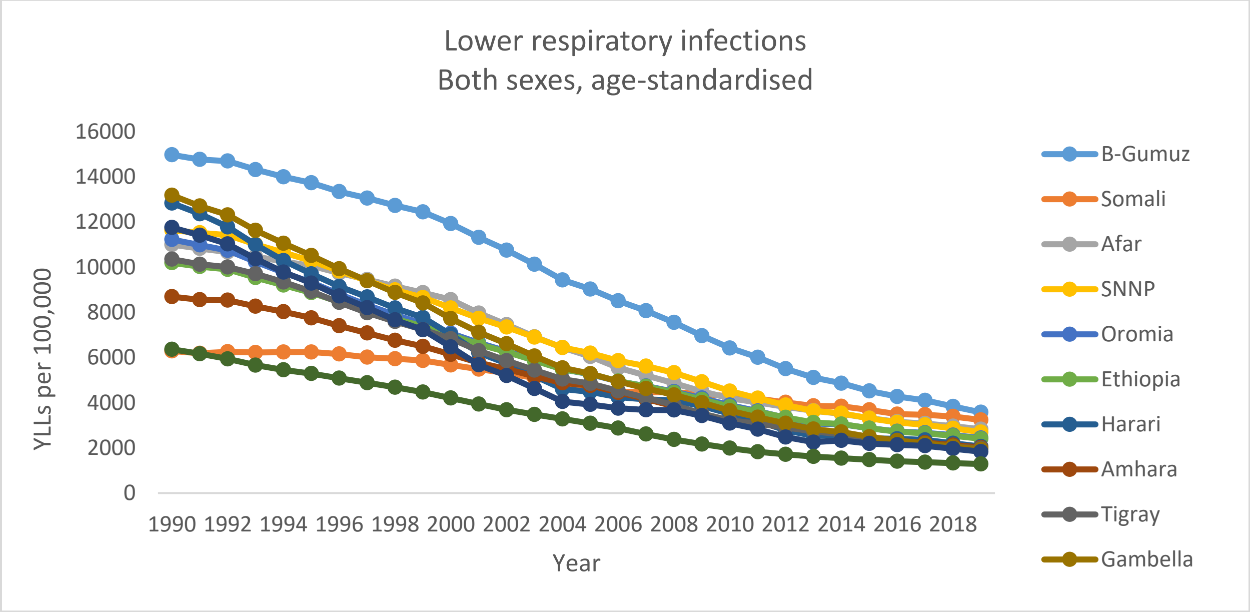

Panel E:

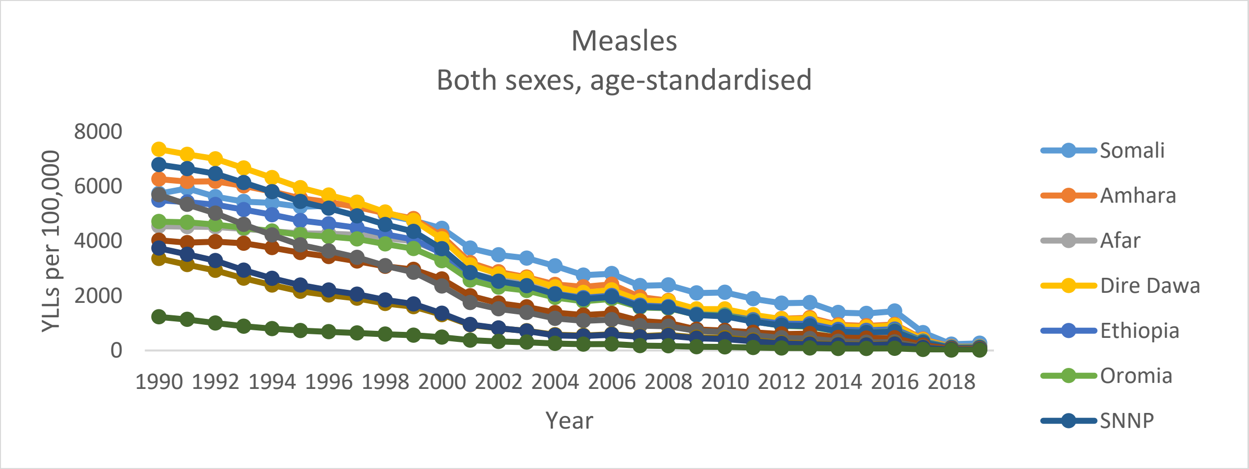
Measles
Both sexes, age-standardised
YLLs per 100,000
8000
6000
4000
2000
0
1990 1992 1994 1996 1998 2000 2002 2004 2006 2008 2010 2012 2014 2016 2018
Year
Somali
Amhara
Afar
Dire Dawa
Ethiopia
Oromia
SNNP

Panel F:

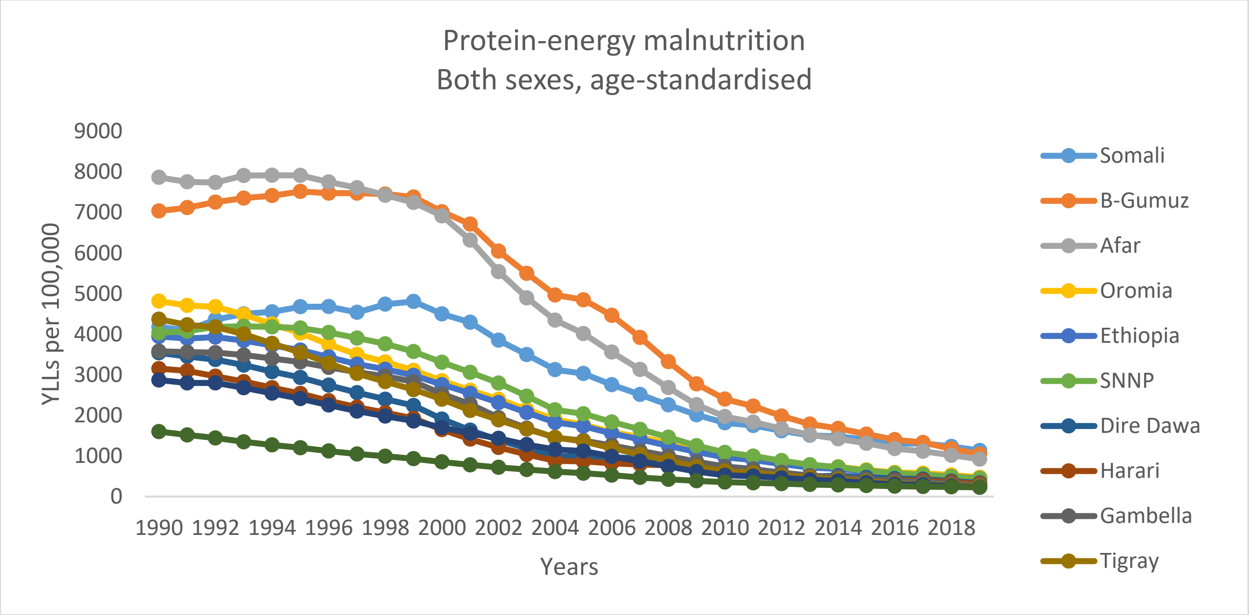
Protein-energy malnutrition
Both sexes, age-standardised
YLLs per 100,000
9000
8000
7000
6000
5000
4000
3000
2000
1000
0
1990 1992 1994 1996 1998 2000 2002 2004 2006 2008 2010 2012 2014 2016 2018
Years
Somali
B-Gumuz
Afar
Oromia
Ethiopia
SNNP
Dire Dawa
Harari
Gambella
Tigray

Panel G:

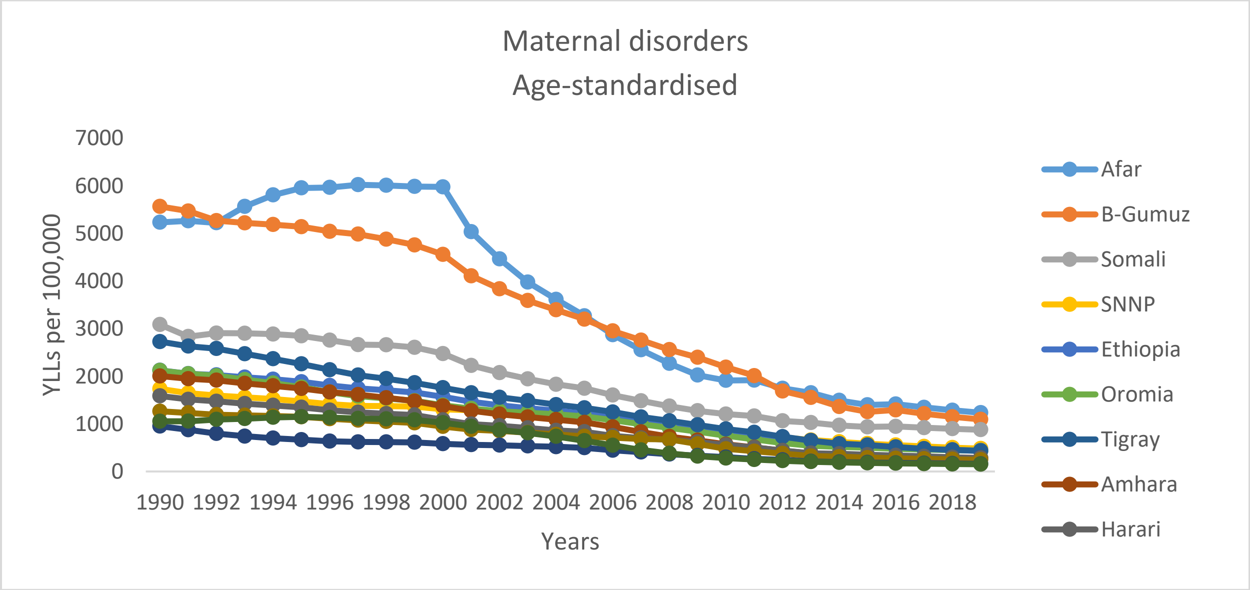

Maternal disorders
Age-standardised
7000
6000
5000
4000
3000
2000
1000
0
YLLs per 100,000
1990 1992 1994 1996 1998 2000 2002 2004 2006 2008 2010 2012 2014 2016 2018
Years
Afar
B-Gumuz
Somali
SNNP
Ethiopia
Oromia
Tigray
Amhara
Harari

Tables

Table S1. SDI variance between 9 regions and 2 chartered cities in Ethiopia, 1990-2019

Year	Ethiopia	Addis Ababa	Afar	Amhara	B/ Gumuz	Dire Dawa	Gambella	Harari	Oromia	Somali	SNNP	Tigray	SDI Variance
1990	0.13	0.38	0.08	0.10	0.10	0.26	0.16	0.27	0.11	0.04	0.11	0.13	0.01
1991	0.13	0.39	0.07	0.10	0.10	0.27	0.16	0.27	0.10	0.02	0.11	0.13	0.01
1992	0.14	0.41	0.08	0.11	0.10	0.29	0.17	0.29	0.11	0.04	0.12	0.14	0.01
1993	0.14	0.42	0.08	0.11	0.10	0.30	0.18	0.30	0.12	0.05	0.12	0.14	0.01
1994	0.14	0.43	0.08	0.11	0.10	0.30	0.18	0.31	0.12	0.05	0.12	0.14	0.01
1995	0.15	0.43	0.08	0.11	0.10	0.31	0.19	0.31	0.12	0.05	0.12	0.14	0.01
1996	0.15	0.44	0.08	0.11	0.10	0.31	0.19	0.31	0.12	0.04	0.12	0.14	0.01
1997	0.15	0.44	0.08	0.11	0.11	0.31	0.19	0.31	0.12	0.02	0.12	0.15	0.02
1998	0.16	0.45	0.09	0.12	0.11	0.32	0.20	0.32	0.13	0.05	0.13	0.15	0.02
1999	0.16	0.46	0.09	0.12	0.12	0.33	0.20	0.33	0.13	0.05	0.13	0.16	0.02
2000	0.16	0.46	0.09	0.12	0.11	0.32	0.20	0.32	0.13	0.04	0.13	0.16	0.02
2001	0.16	0.46	0.09	0.12	0.12	0.32	0.20	0.31	0.13	0.02	0.13	0.16	0.02
2002	0.16	0.46	0.09	0.13	0.12	0.32	0.20	0.32	0.13	0.02	0.14	0.17	0.02
2003	0.17	0.47	0.09	0.13	0.12	0.33	0.21	0.32	0.14	0.02	0.14	0.17	0.02
2004	0.17	0.47	0.10	0.14	0.13	0.33	0.21	0.33	0.14	0.03	0.15	0.18	0.02
2005	0.18	0.49	0.11	0.15	0.14	0.34	0.23	0.34	0.15	0.05	0.16	0.19	0.02
2006	0.19	0.50	0.12	0.16	0.15	0.35	0.24	0.35	0.16	0.06	0.17	0.20	0.02
2007	0.21	0.51	0.13	0.17	0.16	0.36	0.25	0.36	0.17	0.08	0.18	0.22	0.02
2008	0.22	0.52	0.14	0.18	0.18	0.38	0.27	0.37	0.19	0.09	0.20	0.23	0.02
2009	0.23	0.53	0.16	0.20	0.19	0.39	0.29	0.39	0.20	0.11	0.21	0.25	0.02
2010	0.25	0.55	0.17	0.21	0.21	0.40	0.30	0.40	0.22	0.12	0.23	0.26	0.02
2011	0.26	0.56	0.18	0.22	0.22	0.42	0.32	0.41	0.23	0.13	0.24	0.27	0.02
2012	0.27	0.57	0.19	0.23	0.24	0.42	0.33	0.42	0.24	0.14	0.25	0.28	0.02
2013	0.28	0.58	0.20	0.25	0.25	0.44	0.34	0.43	0.25	0.15	0.26	0.30	0.02
2014	0.29	0.59	0.21	0.26	0.26	0.45	0.35	0.44	0.27	0.16	0.28	0.31	0.02
2015	0.30	0.60	0.22	0.27	0.27	0.46	0.37	0.45	0.27	0.16	0.29	0.32	0.02
2016	0.31	0.61	0.23	0.28	0.28	0.47	0.38	0.46	0.29	0.17	0.30	0.33	0.02
2017	0.32	0.62	0.24	0.29	0.30	0.48	0.39	0.48	0.30	0.18	0.31	0.34	0.02
2018	0.33	0.63	0.25	0.30	0.31	0.49	0.40	0.49	0.31	0.19	0.32	0.35	0.02
2019	0.34	0.64	0.26	0.31	0.32	0.50	0.41	0.49	0.32	0.19	0.33	0.36	0.02
% change from, 1990-2019													-54%

Table S2. LE for regions and chartered cities, both sexes, females and males in Ethiopia, 1990 to 2019

Location	Both Sex			Females			Males		
	LE ,1990 (95% UI)	LE,2019 (95% UI)	LE increase, 1990-2019	LE ,1990 (95% UI)	LE,2019(95 % UI)	LE increase, 1990-2019	LE ,1990(95% UI)	LE,2019(95% UI)	LE increase, 1990-2019
Ethiopia	46.91 (45.71-48.11)	68.84 (67.51-70.18)	21.93	49.5 (48.04-51.05)	70.8 (69.24-72.27)	21.3	44.71 (43.25-46.15)	67.13 (65.12-69.2)	22.42
Addis Ababa	53.55 (51.67-55.41)	70.86 (68.91-72.65)	17.31	55.05 (52.43-57.83)	72.19 (69.26-74.92)	17.14	52.03 (49.65-54.19)	69.45 (66.8-72.68)	17.42
Afar	40.61 (38.71-42.68)	63.74 (61.53-66.01)	23.13	39.54 (36.7-42.61)	61.95 (58.83-65.15)	22.41	41.78 (39.6-44.17)	65.04 (61.82-68.35)	23.26
Amhara	46.18 (44.57-47.7)	69.3 (67.54-71)	23.12	48.51 (46.33-50.77)	72.33 (70.25-73.83)	23.82	44.17 (42.45-46.02)	66.7 (64.17-69.34)	22.53
B-Gumuz	39.44 (37.29-41.37)	64.28 (61.99-66.63)	24.84	37.89 (35.07-40.81)	62.91 (59.84-66.12)	25.02	41.23 (38.86-43.68)	65.55 (62.24-68.86)	24.32
Dire Dawa	48.72 (46.63-51.02)	70.04 (68.06-71.85)	21.32	52.1 (49.01-55.41)	71.89 (69.2-73.97)	19.79	45.73 (43.63-48.05)	68.26 (65.28-70.81)	22.53
Gambella	46.6 (44.64-48.66)	67.76 (65.63-69.68)	21.16	52.8 (49.92-55.96)	72.68 (70.66-74.53)	19.88	41.39 (39.27-43.52)	64 (61.02-66.96)	22.61
Harari	46.17 (44.09-48.45)	69.37 (67.34-71.44)	23.20	50.47 (47.15-53.85)	71.65 (68.79-74.3)	21.18	41.85 (39.83-44.2)	67.16 (64.21-70.34)	25.31
Oromia	47.24 (45.71-48.78)	70.03 (68.39-71.58)	22.79	49.77 (47.58-51.9)	71.05 (69.16-73.03)	21.28	45.11 (43.33-46.95)	69.1 (66.68-71.59)	23.99
Somali	51.69 (49.8-53.76)	65.44 (63.13-67.78)	13.75	49.45 (46.95-52.25)	66.14 (63.19-69.08)	16.69	54.64 (52.35-57)	64.96 (61.81-68.42)	10.32
SNNP	46.28 (44.69-47.87)	67.36 (65.66-69.09)	21.08	49.7 (47.45-52.1)	69.79 (67.39-71.98)	20.09	43.67(41.76-45.56)	65.26(62.84-68.09)	21.59
Tigray	46.66(44.79-48.6)	70.3(68.52-72.19)	23.64	48.96(46.58-51.61)	71.7(69.18-74.1)	22.74	44.61(42.47-46.85)	68.97(66.34-71.59)	24.36

Table S3. LE and TFR variance and percent change between regions and chartered cities in Ethiopia, 1990-2019

**Years**	**LE**	**LE**	**LE**	**TFR**
	Male	*Female*	*Both*	*TFR*
1990	19.29	27.52	16.68	0.723
2000	8.43	30.60	13.03	1.128
2010	7.85	20.83	11.25	0.895
2019	3.75	15.48	6.43	1.122
% change from, 1990-2019	81%	44%	61%	-55%

Table S4. Female LE and TFR in regions and chartered cities in Ethiopia, 2019

**Location**	**Female LE, 2019**	**Level**	**TFR, 2019**	**Level**
Addis Ababa	72.19	High	1.50	Low
Dire Dawa	71.89	High	2.95	Low
Gambella	72.68	High	2.79	Low
Amhara	72.33	High	3.87	Low
Harari	71.65	High	3.01	Low
Tigray	71.7	High	3.65	Low
Oromia	71.05	High	4.98	High
Somali	66.14	Low	6.41	High
B-Gumuz	62.91	Low	4.32	High
SNNP	69.79	Low	4.59	High
Afar	61.95	Low	4.95	High

Table S5. Age-standardised death, YLL, YLD and DALY rates per 100 000 populations for all causes combined and leading ten causes in Ethiopia's regions and cities, both sexes, 2019

	**Causes and conditions**	**Ethiopia**	**Addis Ababa**	**Afar**	**Amhara**	**B/Gumuz**	**Dire Dawa**	**Gambella**	**Harari**	**Oromia**	**Somali**	**SNNP**	**Tigray**
Death both sexes	All causes	993.52 (914.97-1070.55)	943.24 (836.87-1055.38)	1353.38 (1195.69-1526.19)	955.41 (855.37-1065.85)	1215.37 (1046.64-1394.05)	930.76 (824.93-1054.72)	1097.57 (985.2-1238.38)	981.41 (865.09-1101.04)	931.40(842.89-1028.21)	1175.51 (1007.84-1366.86)	1091.62 (982.79-1211.4)	982.61 (869.47-1098.19)
	Stroke	90.18(70.62-110.04)	116.16(96.78-137.59)	129.95(101.08-160.22)	89.38(63.85-113.96)	114.07(87.35-144.52)	90.11(66.51-113.88)	113.51(82.92-142.58)	102.69(78.39-126.79)	77.46(57.53-98.33)	106.3(76.92-137.63)	90.64(70.53-112.85)	117.89(91.08-147.91)
	Lower respiratory infections	86.44(75.36-97.65)	59.41(49.81-71.44)	103.71(86.62-122.62)	74.37(59.4-91.02)	101.84(84.02-121.72)	69.94(56.55-84)	82.41(68.39-97.19)	77.06(63.09-92.52)	89.33(75.91-103.18)	97.58(79.26-118.67)	99(83.81-116.65)	95.97(67.34-123.71)
	Ischemic heart disease	84.24(62.56-105.75)	116.47(93.99-139.84)	118.22(88.68-154.84)	82.64(57.25-108.94)	103.98(76.61-136.98)	84.28(57.62-109.99)	106.02(75.34-139.83)	91.96(67.71-116.29)	74.99(52.08-97.73)	93.89(67-126.02)	86.85(65.02-111.24)	84.63(69.26-100.15)
	Diarrheal diseases	76.45(45.12-112.15)	24.42(9.55-44.34)	101.11(55.82-151.57)	83.64(42.9-130.29)	106.56(58.68-168.74)	49.08(26.15-79.26)	66.2(31.72-106.05)	52.24(26.81-86.27)	71.44(43.92-110.98)	99.69(55.99-155.16)	83.72(51.2-124.32)	61.69(32.58-98.04)
	Tuberculosis	60.9(50.44-71.5)	45.2(37.71-55.2)	140.35(113.05-171.19)	59.77(42.36-79.78)	100.81(81.21-123.74)	47.07(36.58-58.63)	76.77(61.29-94.68)	61.04(46.06-75.71)	52.46(42.94-64.03)	105.8(78.4-148.83)	67.41(55.73-81.7)	50.18(39.61-62.96)
	Cirrhosis and other CLD	52.18(44.17-62.07)	47.21(38.14-58.42)	63.37(48.41-82.03)	50.3(37.16-73.63)	55.8(42.99-73.42)	47.32(35.8-63.72)	55.58(44.53-68.52)	50.43(39.35-64.7)	49.92(40.85-60.24)	55.48(41.23-75.1)	62.08(50.89-74.44)	33.76(19.21-50.31)
	Neonatal disorders	44.31(34.92-56.99)	12.89(8.85-18.16)	37.53(28.99-49.26)	48.01(37.44-62.34)	53.97(41.45-71.03)	40.5(30.27-53.58)	31.55(23.57-41.73)	40.2(30.05-53.01)	44.61(35.47-56.81)	48.92(38.58-62.57)	44.61(34.6-58.11)	36.64(19.77-61.88)
	Diabetes mellitus	36(31.01-41.43)	39.85(33-47.63)	44.72(37.44-53.71)	31.52(25.13-39.14)	39.14(32.12-47.98)	34.1(26.7-42.03)	38.48(31.52-46.44)	37.03(29.18-45.21)	35.35(29.16-41.8)	35.99(28.49-44.91)	42.62(35.42-50.85)	45.32(36.11-56.03)
	HIV/AIDS	33.56(28.68-39.58)	95.61(71.23-125.38)	60.67(40.56-86.47)	40.12(29.07-53.18)	26.19(14.9-43.64)	67.09(40.51-103.01)	91.96(56.85-139.75)	38.84(24.5-65.11)	23.86(16.38-33.96)	42.96(31.4-59.89)	20.22(13.61-29.17)	30.77(23.75-40.55)
	Hypertensive heart disease	31.97(17.85-53.09)	34.42(16.91-54.07)	37.37(20.89-59.95)	31.51(16.76-54.86)	36.42(19.54-59.42)	31.79(16.06-56.11)	34.41(18.72-62.25)	36.17(18.43-61.89)	29.85(16.12-51.79)	31.74(16.68-56.83)	34.04(20.02-53.15)	28.76(6.81-76.63)

	**Causes and conditions**	**Ethiopia**	**Addis Ababa**	**Afar**	**Amhara**	**B/Gumuz**	**Dire Dawa**	**Gambella**	**Harari**	**Oromia**	**Somali**	**SNNP**	**Tigray**
YLLs both sexes	All causes	30188.15(27335.78-33522.76)	24587.96(21376.63-28479.55)	40646.83(35269.66-46519.61)	29627.03(26153.31-33252.03)	40246.93(34763.93-46458.09)	27923.42(24318.73-31972.61)	31423.03(27436.21-36073.92)	29023.21(25052.82-33498.29)	27993.55(24822.94-31877.11)	37678.08(32787.59-43171.12)	32890.51(29184.45-37155.55)	26435.52(22998.71-30181.61)
	Neonatal disorders	3936(3102.02-5062.36)	1144.89(786.19-1613.71)	3334.17(2575.7-4376.74)	4263.88(3325.45-5535.56)	4794.17(3682.23-6308.66)	3598.47(2689.6-4760.63)	2802.72(2094.7-3707.8)	3571.17(2669.98-4709.61)	3962.85(3151.45-5047.45)	4346.19(3427.41-5558.73)	3963.41(3074.49-5162.24)	2734.15(2110.52-3602.85)
	Diarrheal diseases	2679.41(1823.93-3760.22)	706.94(346.09-1160)	3028.74(1858.68-4359.95)	2872.53(1619.57-4669.36)	3468.05(2075.83-5236.29)	1527.28(863.89-2479.98)	1765.71(941.39-2671.89)	1696.78(949.97-2706.74)	2636.66(1799.45-3752.16)	3189.06(2085.43-4620.42)	2883.22(1933.89-4093.83)	1885.83(1160.09-2787.08)
	Lower respiratory infections	2404.57(2059.41-2833.31)	1285.69(1065.07-1561.83)	2824.7(2323.08-3414.07)	2016.49(1551.49-2541.83)	3571.17(2772.41-4510.79)	1832.81(1407.35-2363.18)	1937.46(1567.7-2344.65)	2060.45(1595.62-2623.15)	2433.84(2042.25-2879.55)	3236.48(2537.45-4006.03)	2698.2(2243.52-3250.28)	1977.15(1593.94-2395.05)
	Tuberculosis	1729.19(1421.57-2049.86)	1257.74(987.65-1637.78)	4224.43(3303.11-5286.23)	1694(1206.7-2252.3)	3122.54(2515.9-3818.3)	1265.73(961.97-1615.11)	2108.48(1620.78-2668.38)	1708.9(1268.87-2211.91)	1424.64(1145.31-1764.27)	3114.68(2316.65-4344.2)	1932.74(1568.38-2350.92)	1353.68(1052.73-1707.25)
	Stroke	1639.45(1277.27-1998.83)	2076.73(1734.67-2511.62)	2643.28(2063.5-3255.2)	1585.87(1113.95-2048.27)	2265.59(1735.78-2873.04)	1572.81(1132.77-2021.77)	2089.66(1505.93-2660.73)	1844.51(1379.32-2326.34)	1382.54(1033.51-1753.97)	2029.76(1472.25-2610.22)	1694.69(1329.04-2112.14)	2065.02(1571.61-2618.6)
	HIV/AIDS	1581.17(1311.51-1935.43)	4381.86(3213.4-5800.05)	2828.57(1852.03-4134.58)	1922(1387.5-2530.78)	1323.3(734.37-2257.41)	3369.6(2037.19-5146.92)	4584.14(2776.25-7087.13)	2102.84(1264.16-3429.05)	1154.69(791.12-1689.22)	2024.87(1418.98-2847.11)	954.79(610.25-1395.8)	1592.44(895.8-2417.13)
	Ischemic heart disease	1524.69(1133.11-1925.66)	2119.12(1686.63-2595.35)	2391.79(1823.27-3112.12)	1469(1013.35-1978.65)	2036.72(1495.64-2644.13)	1460.62(979.94-1944.49)	1968.05(1389.57-2644.08)	1636.32(1184.83-2099.02)	1317.64(915.29-1719.34)	1786.75(1267.81-2396.47)	1615.9(1213.31-2075.9)	1691.52(1175.78-2229.61)
	Cirrhosis and other chronic liver diseases	1331.56(1095.91-1625.94)	1227.62(953.12-1582.71)	1693.13(1249.58-2210.45)	1269.74(912.09-1954.72)	1492.25(1132.75-1986.18)	1171.79(852.79-1648.64)	1400.83(1101.59-1750.5)	1282.17(959.15-1731.43)	1250.89(1002-1549.27)	1451.64(1072.82-2016.43)	1612.03(1281.65-1974.9)	1119.7(857.99-1431.6)
	Congenital birth defects	735.83(447.45-1200.08)	294.29(148.31-529.45)	583.05(363.12-963.96)	866.04(441.58-1475.29)	1023.6(524.59-1778.98)	614.3(370.17-995.81)	444.15(286.9-696.67)	607.49(374.86-972.76)	689.72(427.97-1142.24)	862.47(474.74-1444.55)	745.48(429.23-1259.86)	552.78(362.72-840.05)

	**Causes and conditions**	**Ethiopia**	**Addis Ababa**	**Afar**	**Amhara**	**B/Gumuz**	**Dire Dawa**	**Gambella**	**Harari**	**Oromia**	**Somali**	**SNNP**	**Tigray**
	Diabetes mellitus	706.24(606.76-813.68)	781.49(643.35-962.41)	948.96(782.94-1140.39)	609.84(478.05-767.44)	822.09(655.85-1010.38)	641.84(496.93-804.18)	758.63(610.59-934.72)	722.81(567.26-908.61)	676.1(557.15-807.1)	730.31(576.84-909.92)	858.25(705.57-1039.85)	727.59(589.3-884.16)
YLDs both sexes	All causes	10802.18(8088.52-13958.5)	10425.76(7851.91-13353.63)	11290.94(8406.9-14586.17)	10674.64(7935.83-13944.33)	10773.61(8074.54-13898.43)	11053.29(8287.48-14208.54)	11340.23(8536.87-14745.69)	10943.17(8202.74-14065.21)	10788.48(8029.18-13971.66)	11507.02(8592.43-14929.86)	10810.59(8100.11-14049.02)	10640.49(8034.05-13798.2)
	Depressive disorders	808.7(562.26-1115.11)	815.88(565.46-1131.74)	784.23(545.69-1097.05)	809(558.62-1110.67)	796.47(554.36-1096.28)	803.15(559.64-1104.89)	802.73(557.59-1097.05)	803.37(557.81-1115.31)	815.62(566.05-1125.68)	775.5(538.06-1064.77)	807.2(560.9-1108.03)	805.12(560.02-1111.36)
	Low back pain	700.06(491.31-935.19)	636.61(446.46-858.78)	676.16(475.66-911.8)	693.74(489.41-929.96)	699.34(489.67-938.81)	650.5(456.87-872.68)	697.75(492.01-936.59)	649.95(457.22-872.7)	714.15(502.15-955.39)	680.59(478.97-918.01)	708.7(496.05-948.98)	696.2(491.44-939.63)
	Age-related and other hearing loss	552.7(377.77-779.89)	497.84(339.19-705.42)	570.96(390.61-803.1)	556.77(381.14-783.62)	555.14(380.23-783.62)	522.77(355.95-735.71)	534.19(363.88-753.38)	521.43(356.4-732.51)	555.32(382.38-785.18)	581.23(399.65-815.78)	548.43(375.68-766.77)	546.33(378.45-764.55)
	Gynecological diseases	470.78(322.13-659.38)	481.29(328.21-675.5)	455.22(312.7-644.82)	469.82(319.18-658.88)	449.66(306.39-628.28)	470.41(321.92-661.86)	495.18(337.64-693.84)	460.07(315.67-644.58)	466.96(319.69-653.75)	465.32(319.97-653.11)	474.78(326.61-668.56)	481.39(328-672.86)
	Blindness and vision loss	420.96(303.1-568.56)	377.4(269.11-513.85)	345.37(246.9-475.25)	398.11(287.31-538.57)	340.08(243.35-466.43)	460.07(326.94-618.74)	433.44(309.91-586.76)	480.91(345.92-648.06)	398.76(285.04-540.94)	361.65(257.49-494.55)	561.13(399.88-752.72)	373.54(265.27-508.91)
	Dietary iron deficiency	396.03(261.75-579.23)	270.78(170.88-410)	677.33(445.84-1002.21)	279.26(178.41-425.24)	295.35(188.69-443.84)	655.76(428.15-947.89)	384.96(237.99-575.77)	498.52(309.93-740.57)	419.97(271.37-623.79)	880.51(584.83-1267.92)	317.82(203.95-467.85)	363.84(229.32-542.94)
	Headache disorders	353.16(95.74-736.24)	356.48(95.1-742.66)	361.15(100.71-741.56)	354.09(94.52-733.63)	366.38(103.59-758.09)	369.69(106-758.05)	371.41(104.49-770.89)	368.03(105.07-769.93)	353.14(94.41-734.78)	359.97(101.68-735.93)	341.02(92.98-711.45)	371.54(104.53-763.61)
	Anxiety disorders	331.14(230.89-451.16)	337.91(236.5-467.1)	323.11(222.71-450.05)	331.25(227.31-458.4)	327.69(228.42-452.45)	332.8(232.93-452.71)	333.21(231.46-459.7)	331.18(228.85-454.05)	331.21(231.77-452.36)	321.53(223.56-443.42)	331.6(231.11-453.17)	333.77(234.71-463.46)

	Causes and conditions	Ethiopia	Addis Ababa	Afar	Amhara	B/Gumuz	Dire Dawa	Gambella	Harari	Oromia	Somali	SNNP	Tigray
	Neonatal disorders	307.52(235.31-386.04)	415.97(324.17-521.25)	343.94(261.06-430.83)	296.22(222.38-381.17)	244.58(178.2-320.99)	320.54(242.03-415.85)	332.68(253.74-416.02)	311.07(232.47-396.07)	316.12(236.97-400.83)	294.52(225.09-375.65)	278.85(207.04-362.21)	340.95(264.91-428.56)
	Diabetes mellitus	273.67(189.12-376.26)	304.6(208.84-414.09)	329.58(226.31-454.22)	252.09(172.3-347.55)	305.7(209.15-416.91)	272.21(184.97-375.16)	286.69(197.43-390.81)	294.59(199.71-403.99)	267.03(183.63-366.12)	292.86(200.32-401.45)	301.49(206.14-415.61)	275.06(188.07-377.79)
DALYs both sexes	All causes	40990.33(36897.07-45655.97)	35013.73(30677.01-39789.77)	51937.77(45903.75-58657.52)	40301.67(35840.43-45093.49)	51020.53(44712.4-58120.7)	38976.71(34231.93-44244.43)	42763.26(37387.28-48345.43)	39966.38(34978.88-45495.8)	38782.03(34594.62-43761.05)	49185.09(43088.93-55654.39)	43701.09(38844.18-49315.4)	37076.01(32498.49-42007.13)
	Neonatal disorders	4243.53(3407.87-5383.87)	1560.86(1184.97-2048.87)	3678.11(2912.53-4714.01)	4560.1(3632.64-5842.72)	5038.75(3911.71-6573.12)	3919.01(3014.94-5092.33)	3135.4(2420.92-4068.21)	3882.25(2963.08-5013.78)	4278.97(3442.66-5377.99)	4640.71(3725.92-5857.79)	4242.27(3354.08-5420.3)	3075.1(2470.85-3937.54)
	Diarrheal diseases	2898.12(2035.97-3973.63)	883.62(519.22-1368.2)	3238.45(2060.54-4572.55)	3096.17(1847.57-4883.5)	3719.35(2338.4-5472.5)	1709.42(1023.41-2655.86)	1985.51(1155.12-2895.64)	1896.61(1151.37-2918.7)	2852.77(1992.59-3944.49)	3419.24(2318.61-4840.49)	3110.23(2148.2-4327.32)	2090.39(1346.77-3000.98)
	Lower respiratory infections	2415.69(2067.8-2845.72)	1294.6(1075.94-1572.77)	2837.33(2338.11-3426.68)	2026.77(1558.85-2552.84)	3583.45(2784.79-4520.95)	1842.26(1415.77-2373.02)	1947.45(1579.22-2359.05)	2069.93(1605.95-2631.56)	2445.48(2052.39-2890.3)	3248.92(2550.29-4023.32)	2709.11(2251.06-3261.28)	1988.83(1603.35-2405.1)
	Tuberculosis	1853.05(1539.82-2163.82)	1365.9(1091.7-1739.66)	4394.22(3500.95-5424.73)	1827.64(1334.64-2397.19)	3282.33(2672.85-3988.04)	1366.14(1062.32-1718.69)	2230.8(1742.13-2787.01)	1827.61(1389.91-2335.15)	1535.95(1263.04-1869.92)	3281.42(2478.07-4529.64)	2059.99(1690.93-2495.88)	1459.48(1160.08-1813.77)
	Stroke	1777.86(1411.33-2139.63)	2288.71(1939.56-2701.26)	2803.48(2234.98-3407.61)	1718.41(1236.2-2180.15)	2424.41(1880.92-3038.22)	1738.91(1294.21-2185.32)	2250.63(1655.91-2814.25)	2023(1549.06-2524.64)	1516.09(1161.15-1880.54)	2176.19(1619.33-2760.15)	1821.65(1445.18-2239.56)	2214.29(1709.96-2762.43)
	HIV/AIDS	1699.06(1423.54-2050.66)	4741.01(3524.37-6230.74)	3009.81(1996.96-4377.88)	2064.31(1522-2726.2)	1427.33(807.87-2364.19)	3639.48(2268.8-5497)	4982.49(3126.23-7529.25)	2325.68(1429.12-3748.3)	1242.27(852.62-1783.85)	2118.08(1495.38-2961.89)	1023.02(668.79-1488.18)	1723(990.03-2584.84)

	Causes and conditions	Ethiopia	Addis Ababa	Afar	Amhara	B/Gumuz	Dire Dawa	Gambella	Harari	Oromia	Somali	SNNP	Tigray
	Ischemic heart disease	1579.98(1184.72-1974)	2180.6(1745.92-2649.5)	2456.51(1886.94-3173.22)	1522.95(1064.49-2027.62)	2097(1554.05-2731.21)	1517.08(1036.11-1987.75)	2025.26(1449.91-2693.88)	1692.41(1239.79-2153.44)	1373.85(973.66-1775.11)	1851.79(1326.42-2450.78)	1666.45(1265.22-2134.93)	1746.38(1234.34-2282.81)
	Cirrhosis and other CLD	1341.44(1106.26-1637.42)	1238.39(963.18-1594.02)	1703.31(1258.23-2219.46)	1279.48(922.03-1965.74)	1502.28(1142.48-1994.06)	1181(860.54-1659.72)	1410.27(1109.31-1758.63)	1291.36(969.83-1740.35)	1260.5(1015.22-1559.15)	1461.72(1084.96-2028.88)	1622.55(1292.62-1985.18)	1129.05(866.49-1440.11)
	Diabetes mellitus	979.91(849.79-1115.68)	1086.09(916.96-1276.16)	1278.55(1076.67-1489.03)	861.93(702.22-1040.72)	1127.8(932.74-1340.89)	914.06(750.84-1091.97)	1045.32(870.92-1233.25)	1017.41(828.7-1218.99)	943.12(804.51-1093.68)	1023.16(845.73-1228.82)	1159.74(974.45-1365.04)	1002.65(825.12-1181.7)

Table S6. Age-standardised death, YLL, YLD, and DALY rates for 100 000 population, both sexes for the leading 11th-20th causes in Ethiopia's regions and chartered cities, 2019

	**Conditions and causes**	**Ethiopia**	**Addis Ababa**	**Afar**	**Amhara**	**B/Gumuz**	**Dire Dawa**	**Gambella**	**Harari**	**Oromia**	**Somali**	**SNNP**	**Tigray**
Death both sexes	Chronic obstructive pulmonary disease	28.32(22.68-33.35)	22.85(16.88-28.26)	32.05(21.92-45.83)	28.46(22.17-38.11)	27.64(17.93-40.11)	20.12(14.43-26.6)	26.21(19.13-34)	23.16(16.54-29.01)	26.99(20.6-32.21)	29.44(18.85-50.63)	31.21(24.15-37.86)	37.98(30.89-45.87)
	Alzheimer's disease and other dementias	27.82(6.68-75.12)	27.13(6.6-74.56)	23.6(5.43-65.31)	26.58(6.62-72.44)	24.48(5.78-67.95)	26.36(6.36-70.85)	25.2(6.01-68.84)	26.77(6.46-73.23)	28.93(6.93-77.37)	24.13(5.66-65.44)	28.8(6.99-77.05)	26.8(21.83-32.75)
	Chronic kidney disease	26.17(22.48-30.08)	26.99(22.3-32.27)	31.6(26.11-38.81)	22.6(17.82-27.8)	28(22.56-34.65)	25.79(21.13-30.78)	27.24(22.08-32.77)	28.07(23.25-33.34)	26.4(21.43-31.3)	26.14(20.38-33.81)	30.8(25.61-36.63)	30.54(23.71-39.76)
	Road injuries	14.51(12.16-17.36)	12.86(9.89-16.03)	19.37(15.31-24.19)	12.94(10.13-16.31)	17.11(13.84-21.17)	14.06(9.05-22)	15.42(12.09-19.85)	14.57(10.15-23.99)	13.87(11.31-17.1)	16.37(12.71-20.75)	17.79(14.31-22.52)	10.35(7.63-13.6)
	Meningitis	14.14(12-16.55)	10.15(8.25-12.51)	23.78(19.04-29.34)	12.88(9.83-16.55)	20.01(15.89-24.49)	10.35(8.16-12.92)	13.09(10.51-16.1)	12.9(10.12-16.16)	13.11(10.81-15.78)	20.68(16.1-26.3)	16.05(13.33-19.23)	8.95(5.82-14.27)
	Protein-energy malnutrition	12.9(9.77-16.7)	11.21(9.2-13.43)	35.56(27.54-45.51)	5.93(2.54-11.14)	27.52(21.07-34.18)	12.57(8.93-18.56)	12.9(8.84-17.04)	12.23(8.47-18.06)	14.2(10.71-17.95)	33.34(23.07-46.02)	13.89(10.2-18.57)	8.09(5.12-12.16)
	Self-harm	10.13(8.21-12.9)	9.12(6.95-12.18)	13.45(10.53-17.6)	8.83(6.37-12.88)	11.14(8.69-14.44)	7.82(5.79-10.93)	10.39(7.56-14.69)	8.75(6.49-12.25)	9.48(7.42-12.49)	10.77(8.07-14.8)	13.89(11.11-17.56)	11.14(8.18-14.88)
	Breast cancer	9.71(7.96-11.64)	15.45(11.29-20.74)	10.59(7.83-14.02)	7.67(5.28-11.31)	11.29(8.44-14.91)	10.76(8.02-14.88)	8.81(6.5-12.45)	11.89(8.81-15.64)	9.86(7.89-11.85)	6.41(4.24-9.72)	11.36(9.05-13.65)	11.38(8.58-13.97)
	Interpersonal violence	9.68(7.94-11.89)	7.45(5.53-9.96)	14.17(10.04-20.08)	9.61(6.93-12.96)	11.74(8.89-15.43)	8.45(6.12-11.46)	11.23(8.23-15.69)	9.15(6.57-12.5)	8.15(6.43-10.38)	12.33(8.6-17.16)	11.87(9.09-15.09)	8.73(5.92-13.9)

	Falls	9.60(8.09-11.29)	8.24(6.8-10.08)	9.36(7.14-11.83)	9.4(7.26-12.24)	8.71(6.64-11.45)	8.58(6.79-10.78)	9.21(6.89-11.8)	9.17(7.39-11.16)	9.23(7.53-11.12)	8.48(6.52-11)	11.36(9.12-13.72)	6.82(0.88-22.47)
YLL both sexes	Malaria	683(134.17-1805.31)	80.82(4.95-322.86)	373.87(31.02-1173.15)	642.99(81.19-1704.74)	1326.48(162.19-3879.9)	1569.61(176.86-3767.72)	1145.93(255.32-2841.13)	1389.51(208.1-3554.6)	654.69(117.28-1814.73)	634.24(78.43-1750.82)	861.55(194.07-2475.56)	499.02(67.98-1567.23)
	Meningitis	610.64(507.11-731.76)	356.77(278.5-453.27)	965.31(758.53-1204.6)	536.11(404.85-691.23)	975.27(750.02-1240.64)	423.99(311.33-565.46)	481.7(370.82-603.58)	527.38(389-694.81)	563.94(448.04-715.52)	959.33(743.79-1226.97)	700.02(558.84-860.09)	421.36(323.54-526.07)
	Hypertensive heart disease	537.33(299.59-899.12)	578.68(280.46-923.66)	730.6(406.49-1138.62)	516.1(273.57-892.85)	683.18(369.07-1100.28)	513.21(260.79-902.74)	566.79(312.11-1012.36)	590.42(300.06-993.86)	494.93(265.37-867.22)	573.97(304.92-1032.18)	582.15(345.94-918.28)	599.56(321.39-1034.76)
	Chronic kidney disease	522.69(454.91-600.02)	531.12(436.12-648.14)	686.48(561.31-835.23)	446.59(351.84-556.86)	620.57(500.31-770.69)	490.61(397.47-602.27)	531.62(422.7-654.89)	550.98(448.82-669.85)	511.23(426.59-603.31)	545.93(425.15-696.46)	634.24(531.74-754.82)	515.25(417.04-637)
	Road injuries	510.67(422.65-615.89)	431.32(325.37-556.51)	684.58(516.43-873.82)	459.7(359.92-585.84)	667.9(537.38-822.97)	474.43(303.11-745.92)	519.61(393.92-685.82)	507.52(342.8-840.62)	471.67(380.87-589.71)	600.39(463.71-775.64)	633.39(499.45-830.87)	434.66(333.41-567.46)
	Chronic obstructive pulmonary disease	484.13(385.65-573.47)	392.68(290.4-489.06)	554.22(377-806.65)	486.77(375.22-671.88)	480.06(309.41-728.93)	291.6(93.2-668.05)	454.32(327.31-593.83)	397.06(274.17-504.33)	451.63(338.94-543.17)	501.8(322.05-880.26)	550.34(423.27-671.85)	518.63(396.85-679.95)
	Protein-energy malnutrition	446.03(336.29-605.03)	228.99(186.79-280.27)	923.33(690.56-1193.61)	233.46(94.69-473.93)	1044.34(736.16-1426.44)	339.45(211.67-544.92)	289.91(194.23-416.24)	323.28(201.45-527.07)	477.67(335.81-649.84)	1133.4(724.21-1669.51)	436.11(304.55-641.83)	258.09(182.33-359.42)
	Maternal disorders	439.96(318.87-599.92)	151.91(89.11-257.77)	1233.67(784.3-1875.55)	284.02(174.82-426.28)	1094.4(729.35-1510)	246.24(158.28-411.69)	160.01(98.58-236.7)	279.98(169.34-481.56)	435.84(286.62-644.6)	876.6(554.04-1265.93)	480.66(309.6-703.08)	430.55(269.75-645.46)
	Interpersonal violence	408.09(332.52-504.84)	315.89(226.89-434.94)	599.25(409.86-876.16)	402.14(290.01-550.62)	512.11(387.88-676.98)	353.52(247.69-490.2)	468.18(329.91-668.58)	386.75(271.26-541.91)	340.47(263.93-445.33)	524.71(369.67-725.35)	500.62(380.86-643.56)	402.9(296.58-535.61)
	Whooping cough	399.45(41.75-1131.46)	69.49(2.14-279.08)	605.69(26.91-2154.91)	441.06(18.96-1702.75)	453.37(12.5-2061.47)	207.36(8.53-925.82)	282.13(11.17-1086.73)	384.82(10.98-1622.83)	377.68(25.31-1198.75)	643.63(27.19-2382.19)	385.42(15.38-1556.24)	174.25(6.72-710.16)

YLDs both sexes	Alcohol use disorders	258.35(173.38-365.67)	263.07(177.42-374.29)	278.53(184.98-390.16)	256.58(169.65-360.88)	265.89(177.63-373.74)	264.27(177.46-375.05)	257.1(173.01-368.25)	275.12(185.67-388.14)	255.57(170.31-360.68)	273.52(183.9-384.42)	259.27(174.9-370.16)	252.95(169.28-357.57)
	Oral disorders	246.16(129.76-416.59)	248.4(129.14-431.29)	243.95(129.65-414.65)	246.49(131.25-418.36)	245.45(131.1-415.58)	247.01(129.06-422.72)	246.23(129.35-415.76)	247.24(129.24-425.4)	246.09(129.79-416.38)	244.45(131.01-411.42)	245.65(130.32-414.37)	246.45(129.85-416.4)
	Endocrine, metabolic, blood, and immune disorders	228.77(152.94-320.67)	255.22(169.35-359.71)	236.44(159.15-329.32)	221.47(148.14-311.69)	217.19(145.93-307.37)	263.9(176.42-371.57)	233.31(155.5-327.42)	251.24(168.69-353.06)	228.08(152.15-323.81)	248.62(168.22-344.39)	222.57(148.65-313.72)	228.82(154.19-319.6)
	Diarrheal diseases	218.7(150.52-299.77)	176.68(122.81-245.54)	209.71(145.84-292.82)	223.64(156.27-309.68)	251.3(172.87-345.88)	182.14(126.39-251.31)	219.81(151.88-302.6)	199.83(139.07-274.6)	216.11(148.87-299.91)	230.19(157.37-314.9)	227.01(156.14-313.95)	204.56(141.9-282.09)
	Conflict and war	212.31(115.82-430.6)	213.27(122.93-394.7)	168.13(89.47-342.39)	263.32(155.72-448.99)	181.51(97.23-361.93)	190.12(103.33-358.93)	422.18(256.94-661.78)	378.76(227.87-610.05)	175.98(95.44-337.52)	226.3(130.22-381.26)	161.42(54.73-585.96)	341.03(208.08-546.08)
	Road injuries	201.72(147.96-265.31)	193.4(140.27-257.54)	211.68(155.19-277.83)	188.64(138.28-248.04)	214.62(157.15-282.01)	207.87(151.85-273.7)	180.14(131.89-235.8)	209.83(153.68-275.71)	201.76(147.54-265.83)	229.15(167.96-300.98)	216.33(157.43-284.18)	190.96(139.94-251.95)
	Osteoarthritis	194.89(99.24-382.4)	210.16(106.58-416.52)	189.44(96.53-370.53)	194.58(98.98-382.12)	192.66(97.08-377.22)	202.34(102.98-395.07)	197.04(99.85-385.88)	202.17(103.04-395.2)	194.23(98.83-379.98)	188.37(95.25-371.36)	193.57(98.38-381.83)	196.57(98.84-384.49)
	Other musculoskeletal disorders	192.11(103.34-287.74)	264.94(152.63-390.04)	183.21(101.39-273.26)	178.91(93.01-272.56)	196.05(113.42-293.29)	227.49(130.48-336.11)	177.19(85.93-281.91)	192.39(94.95-303.75)	198.88(112.13-297.37)	151.25(79.39-236.41)	195.61(98.79-291.5)	174.06(87.62-275.68)
	Schistosomiasis	165.23(81.25-310.87)	0(0-0)	51(27.64-86.75)	248.86(119.54-474.65)	22.01(10.44-42.17)	99.65(50.51-184.12)	22.02(11.02-42.06)	0(0-0)	151.08(74.53-278.07)	29.22(17.15-47.18)	192.05(93.89-366.78)	164.12(80.2-304.06)
	Chronic obstructive pulmonary disease	144.95(119.38-168.06)	178.64(147.19-206.83)	157.24(130.47-181.61)	137.75(113.32-160.94)	158.74(130.56-184.49)	172.51(143.63-200.13)	165.25(136.23-192.33)	190.43(157.31-219.35)	137.22(112.63-161.52)	147.17(120.25-171.6)	155.83(126.70-182.61)	149.66(123.63-173.44)

DALYs both sexes	Depressive disorders	808.70(562.26-1115.11)	815.88(565.46-1131.74)	784.23(545.69-1097.05)	809.00(558.62-1110.67)	796.47(554.36-1096.28)	803.15(559.64-1104.89)	802.73(557.59-1097.05)	803.37(557.81-1115.31)	815.62(566.05-1125.67)	775.5(538.06-1064.77)	807.20 (560.9-1108.03)	805.12(560.02-1111.36)
	Malaria	738.85(186.18-1870.55)	96.43(20.36-337.73)	443.42(95.82-1235.14)	679.26(110.86-1741.08)	1420.35(246.31-3969.51)	1637.04(251.95-3843.53)	1273.04(362.73-2983.18)	1467.02(296-3605.31)	716.99(172.57-1862.25)	727.08(168.74-1831.42)	919.45(247.26-2582.9)	542.42(109.00-1620.8)
	Road injuries	712.39(604.39-836.71)	624.72(495.27-759.17)	896.26(724.56-1103.52)	648.33(526.86-793.24)	882.53(738.36-1042.26)	682.3(495.57-953.58)	699.75(559.02-879.69)	717.35(539.13-1052.66)	673.42(565.26-812.11)	829.54(677.26-1006.22)	849.72(696.35-1044.57)	625.61(500.91-769.01)
	Low back pain	700.06(491.31-935.19)	636.61(446.46-858.78)	676.16(475.66-911.8)	693.74(489.41-929.96)	699.34(489.67-938.81)	650.5(456.87-872.68)	697.75(492.01-936.58)	649.95(457.22-872.7)	714.15(502.15-955.39)	680.59(478.97-918.01)	708.7(496.05-948.98)	696.2(491.44-939.63)
	Meningitis	640.2(534.77-760.73)	372.36(294.04-471.01)	1002.98(799.35-1243.99)	563.2(432.4-719.2)	1004.4(777.04-1269.52)	444.76(330.82-585.71)	507.81(400.15-630.14)	550.66(413.14-718.66)	594.21(478.28-742.92)	999.09(779.7-1267.92)	732.12(592.47-890.9)	445.12(346.12-549.25)
	Chronic obstructive pulmonary disease	629.09(524.55-724.2)	571.32(462.65-672.01)	711.46(533.63-970.8)	624.52(507.73-803.78)	638.81(464.86-885.93)	503.66(402.01-616.94)	619.57(486.23-762.74)	587.49(463.2-701.6)	588.86(477.89-682.22)	648.97(468.27-1029.33)	706.17(574.89-837.08)	668.29(540.5-834.52)
	Chronic kidney disease	592.99(523.05-674.12)	607.25(508.77-727.05)	754.46(435.85-1165.64)	512.67(417.44-625.64)	694.84(569.71-847.82)	562.57(466.24-680.14)	598.98(489.72-726.77)	623.11(516.29-748.69)	582.04(494.14-676.87)	613.81(493.16-768.75)	706.71(604.57-829.49)	585.83(484.93-706.86)
	Hypertensive heart disease	559.74(322.52-920.44)	602.37(304.22-951.28)	754.46(435.85-1165.64)	538.25(295.71-913.49)	706.46(390.76-1130.4)	535.48(281.93-927.18)	589.37(334.23-1030.84)	612.22(320.23-1017.6)	517.73(288.16-896.15)	598.59(331.88-1062.61)	603.09(364.65-944.18)	621.83(348.98-1054.59)
	Age-related and other hearing loss	552.7(377.77-779.89)	497.84(339.19-705.42)	570.96(390.61-803.1)	556.77(381.14-783.62)	555.14(380.23-783.61)	522.77(355.95-735.71)	534.19(363.88-753.38)	521.43(356.4-732.51)	555.32(382.38-785.18)	581.23(399.65-815.78)	548.43(375.68-766.77)	546.33(378.45-764.55)
	Maternal disorders	496.94(378.56-657.72)	166.92(103.82-272.88)	1297.95(840.11-1942.44)	338.52(232.42-485.61)	1159.42(798.72-1581.93)	295.72(205.98-462.42)	200.08(137.04-273.75)	327.54(212.87-523.65)	496.46(345.68-705.16)	971.55(648.99-1344.7)	537.97(367.29-760.3)	470.46(307.68-685.8)

Table S7. SDI and LE for both sexes by age-standardised YLL rates for 5 leading causes in Ethiopia's regions and chartered cities, both sexes, 2019

Location	SDI	Level	LE, both sexes	Level	Neonatal disorders	Diarrheal diseases	Lower respiratory infections	Tuberculosis	Stroke	Level of YLL
Addis Ababa	0.64	High	71.6	High	1144.89(786.19-1613.71)	706.94(346.09-1160)	1285.69(1065.07-1561.83)	1257.74(987.65-1637.78)	2076.73 (1734.67-2511.62)	Low
Dire Dawa	0.50	High	72.4	High	3598.47(2689.6-4760.63)	1527.28(863.89-2479.98)	1832.81(1407.35-2363.18)	1265.73(961.97-1615.11)	1572.81 (1132.77-2021.77)	Low
Harari	0.49	High	70.9	High	3571.17(2669.98-4709.61)	1696.78(949.97-2706.74)	2060.45(1595.62-2623.15)	1708.9(1268.87-2211.91)	1844.51 (1379.32-2326.34)	Low
Gambella	0.41	High	69.5	Low	2802.72(2094.7-3707.8)	1765.71(941.39-2671.89)	1937.46(1567.7-2344.65)	2108.48(1620.78-2668.38)	2089.66(1505.93-2660.73)	Moderate
Tigray	0.36	Middle	71.1	High	2734.15(2110.52-3602.85)	1885.83(1160.09-2787.08)	1977.15(1593.94-2395.05)	1353.68(1052.73-1707.25)	2065.02(1571.61-2618.6)	Moderate
SNNP	0.33	Middle	67.5	Middle	3963.41(3074.49-5162.24)	2883.22(1933.89-4093.83)	2698.2(2243.52-3250.28)	1932.74(1568.38-2350.92)	1694.69(1329.04-2112.14)	Moderate
B-Gumuz	0.32	Middle	65	Low	4794.17(3682.23-6308.66)	3468.05(2075.83-5236.29)	3571.17(2772.41-4510.79)	3122.54(2515.9-3818.3)	2265.59(1735.78-2873.04)	Moderate
Oromia	0.32	Middle	70.8	High	3962.85(3151.45-5047.45)	2636.66(1799.45-3752.16)	2433.84(2042.25-2879.55)	1424.64(1145.31-1764.27)	1382.54(1033.51-1753.97)	Moderate
Amhara	0.31	Middle	70.2	High	4263.88(3325.45-5535.56)	2872.53(1619.57-4669.36)	2016.49(1551.49-2541.83)	1694(1206.7-2252.3)	1585.87(1113.95-2048.27)	Moderate
Afar	0.26	Low	62.7	Low	3334.17(2575.7-4376.74)	3028.74(1858.68-4359.95)	2824.7(2323.08-3414.07)	4224.43(3303.11-5286.23)	2643.28(2063.5-3255.2)	High
Somali	0.19	Low	65.1	Low	4346.19(3427.41-5558.73)	3189.06(2085.43-4620.42)	3236.48(2537.45-4006.03)	3114.68(2316.65-4344.2)	2029.76(1472.25-2610.22)	High

Table S8. Age-standardised death rates per 100 000 population for all causes combined and leading 20 causes in Ethiopia's regions and chartered cities, women and men, 2019

	**Causes of death**	**Ethiopia**	**Addis Ababa**	**Afar**	**Amhara**	**B/Gumuz**	**Dire Dawa**	**Gambella**	**Harari**	**Oromia**	**Somali**	**SNNP**	**Tigray**
Female deaths	All causes	881.70 (802.03-952.61)	846.31 (711.5-1023.2)	1567.50 (1326.77-1849.8)	783.278 (708.57-891.13)	1345.61 (1106.78-1601.98)	822.79 (720.96-961.82)	779.4 (692.41-875.05)	851.94 (709.24-999.46)	875.11 (769.61-965.82)	1127.46(963.58-1339.99)	935.89 (818.59-1065.21)	896.02 (763.35-1051.7)
	Stroke	87.74 (67.45-107.29)	107.73 (83.57-136.9)	175.02(130.7-227.98)	78.8(51.92-103.05)	148.31(108.5-193.38)	84.7(59.15-113.29)	77.27(47.25-106.31)	95.93(70.49-123.4)	80.88(58.78-102.48)	114.34(83.86-151.05)	86.38(65.46-108.22)	119.47(85.52-153.77)
	Ischemic heart disease	79.84 (56.94-99.74)	100.46 (76-130.69)	155.16(113.87-209.83)	68.76(45.2-93.56)	133.97(97.87-175.16)	79.4(50.82-108.69)	71.66(42.49-97.74)	86.42(60.58-112.71)	79.12(53.48-103.93)	100.89(72.17-136.02)	81.17(58.31-104.6)	92.34(61.12-124)
	Lower respiratory infections	71.82 (60.22-82.99)	47.1 (35.53-62.56)	112.45(87.37-142.38)	57.96(44.15-73.73)	109.25(82.62-138.82)	56.25(42.54-71.38)	47.99(34.65-60.18)	63.04(49.07-79.93)	77.35(62.08-92.42)	88.33(69.03-111.44)	79.26(63.54-95.26)	73.3(56-93.5)
	Diarrheal diseases	63.48 (30.23-128.99)	16.4 (4.78-53.55)	112.73(45.68-218.65)	63.2(29.61-132.41)	99.13(40.42-214.31)	40.34(17.39-86.3)	46.97(20.53-94.41)	42.6(22.61-59.84)	64.94(29.28-137.66)	86.06(37.25-171.15)	72.33(33.43-145.29)	47.24(19.3-109.85)
	Tuberculosis	44.93 (35.5-59.38)	30.72(22.11-42.63)	149.54(109.24-202.94)	39.73(25.04-64.16)	108.2(77.97-142.03)	32.66(20.12-45.32)	34.07(22.91-45.81)	42.13(17.86-90.96)	41.86(31.69-60.24)	94.1(63.16-169.61)	47.44(35.92-66.11)	37.07(27.09-55.42)
	HIV/AIDS	41.66 (34.41-49.73)	127.63(92.71-167.76)	73.8(50.07-104.95)	45.83(30.41-62.28)	30.74(16.62-54.07)	77.18(45.62-122.27)	120.78(68.63-185.47)	40.33(17.86-74.54)	30.97(21.62-43.65)	52.24(37.41-71.58)	25.05(17.06-35.52)	41.93(23.4-61.82)
	Hypertensive heart disease	38.3 (21.34-60.49)	37.56(16.72-63.7)	68.26(34.28-113.82)	34.8(17.86-62.71)	57.5(30.06-85.12)	36.43(16.41-68.41)	33.71(16.45-77.48)	39.09(25.77-58.35)	37.96(20.27-61.9)	45.91(24.26-72.64)	40.4(21.6-58.63)	43.02(23.09-67.4)
	Cirrhosis and other CLD	36.08 (28.24-44.05)	31.99(23.7-43.66)	59(42.49-78.73)	31.15(19.77-46.22)	49.54(33.21-67.55)	33.22(22.46-49.31)	28.27(17.58-43.28)	34.79(24.96-47.6)	37.23(28.43-46.42)	38.96(27.9-52.42)	40.58(30.9-51.04)	36.28(26.48-47.79)
	Neonatal disorders	35.44 (28.32-44.58)	9.52(6.41-13.33)	30.15(23.61-39.05)	38.23(30.04-48.62)	43.2(33.67-55.62)	32.12(24.23-41.71)	24.86(18.82-32.64)	31.84(23.55-41.52)	35.89(28.68-45.2)	39.1(31.28-49.02)	35.54(27.99-45.59)	24.54(19.21-31.48)
	Alzheimer's disease and other dementias	30.08(7.38-79.52)	29.17(7.15-77.02)	27.14(6.2-76.24)	28.24(6.99-75.65)	28.73(6.74-80.04)	27.75(6.74-75.12)	25.59(6.16-69.16)	28.75(21.12-36.99)	31.66(7.67-82.15)	27.44(6.58-74.37)	30.8(7.14-81.38)	31.16(7.47-83.36)
	Diabetes mellitus	28.84(24.01-33.26)	29.16(22.61-38.23)	49.92(38.64-63.37)	22.93(17.13-29.26)	43.64(32.55-57.1)	26.05(18.91-33.65)	20.25(13.83-25.7)	28.05(6.54-77.74)	30.47(24-37.3)	32.77(24.63-43.32)	32.33(25.66-39.52)	31.74(24.3-40.78)
	Chronic kidney disease	21.51(18.03-25.19)	23.2(17.26-30.57)	33.61(25.76-44.61)	16.13(12.23-21.79)	29.82(21.8-40.39)	21.26(15.93-27.31)	15.6(12.32-20.89)	23.21(17.57-29.34)	23.69(18.34-28.61)	21.68(16.07-30.04)	24.81(19.81-30.31)	22.19(16.75-29.5)
	Chronic obstructive pulmonary disease	19.1(13.29-24.52)	15.57(5.22-21.77)	23.35(12.6-46.68)	18.51(12.52-28.6)	21.51(10.98-42.95)	11.9(7.01-18.45)	10.02(6.32-17.71)	14.91(8.82-20.45)	19.07(13.18-24.74)	18.13(9.63-33.54)	19.96(13.35-26.95)	21.74(13.12-29.96)
	Breast cancer	17.36(14.3-20.5)	26.65(18.78-37.71)	25.08(18.34-34.29)	12.91(8.57-19.48)	23.03(16.71-31.31)	18.37(13.46-26.05)	14.18(9.86-20.85)	19.88(14.21-27.05)	18.03(14.14-21.93)	13.7(8.74-21.98)	20.21(15.82-24.82)	17.77(12.77-23.85)

	Causes of death	Ethiopia	Addis Ababa	Afar	Amhara	B/Gumuz	Dire Dawa	Gambella	Harari	Oromia	Somali	SNNP	Tigray
	Cervical Cancer	16.82(11.73-27.12)	15.09(10.28-21.48)	34.01(21.55-48.43)	13.18(8.33-24.4)	27.78(17.79-38.67)	13.37(8.99-21.67)	11.22(7.67-20.15)	14.91(8.82-20.45)	17.41(11.21-30.29)	21.1(13.39-34.52)	18.96(12.22-29.51)	17.51(11.38-27.92)
	Maternal disorders	16.16(11.8-21.87)	5.18(3.03-8.8)	46.85(29.72-70.75)	10.47(6.52-15.73)	41.22(27.33-58.26)	9.19(5.86-15.15)	5.66(3.48-8.39)	10.78(6.53-18.29)	16.17(10.62-23.96)	33.47(21.19-48.46)	17.26(11.19-25.15)	15.57(9.84-23.47)
	Protein-energy malnutrition	12.95(8.71-18.15)	9.78(7.31-12.97)	39.48(26.06-57.35)	7.03(1.75-15.78)	30.56(22.38-40.3)	11.87(7.53-17.15)	9.44(4.12-14.72)	12.08(7.15-18.48)	14.53(9.56-20.5)	40(20.85-64.67)	13.27(8.45-19.75)	11.03(7.11-16.67)
	Meningitis	12.79(10.45-15.71)	8.95(6.55-12.23)	29.97(22.17-39.65)	10.86(7.77-15.73)	23.43(16.61-30.74)	9.01(6.64-12.25)	8.07(6.02-11.48)	11.2(8.02-15.54)	12.4(9.43-15.66)	21.58(15.43-30.51)	13.92(10.95-17.37)	10.59(7.52-14.08)
	Asthma	8.81(4.22-14.89)	5.43(2.46-9)	17.99(10.24-31.2)	8.75(3.27-20.33)	14.67(8.83-22.76)	6.33(3.89-10.99)	6.63(3.26-15.88)	6.99(3.8-11.22)	8.14(3.68-12.61)	12.79(6.98-23.96)	9.5(4.51-14.75)	9.22(4.36-15.48)
	Malaria	8.58(1.69-22.61)	1.08(0.07-4.62)	5.78(0.46-18.79)	7.69(0.98-21.3)	17.57(2.12-52.78)	20.8(2.25-52.04)	15.04(3.32-39.23)	20.04(2.65-54.09)	8.4(1.47-23.56)	8.42(1.02-23.5)	10.81(2.34-31.16)	6.41(0.85-20.58)
Male deaths	All causes	1101.46 (963.36-1246.05)	1059.10 (828.17-1256.09)	1249.80 (1015.7-1517.27)	1135.70 (943.66-1355.44)	1119.87 (899.63-1381.09)	1048.83(879.18-1283.72)	1436.37 (1204.81-1721.87)	1134.65 (917.31-1377.4)	984.89(823.06-1159.58)	1202.89 (958.93-1461.27)	1245.90 (1031.54-1461.81)	1073.23 (889.9-1259.76)
	Lower respiratory infections	100.69(84.1-121.43)	73.63(55.07-98.34)	98.77(76.97-127.31)	92.51(70.55-122.17)	95.84(74.66-124.17)	85.42(67.38-109.56)	120.22(95.68-149.57)	95.76(72.59-123.47)	100.36(79.9-123.59)	101.76(77.26-130.74)	118.82(94.31-146.36)	96.96(74.83-122.33)
	Stroke	92.67(66.85-119.93)	126.31(91.11-159.68)	111.33(77.79-148.51)	100.51(67.23-138.47)	91.14(58.28-128.96)	96.31(65.79-132.78)	151.61(108.67-198.99)	110.64(76.51-150.67)	74.55(49.93-102.39)	103.17(68.26-144.18)	95.11(66.94-128.47)	115.85(79.9-154.57)
	Diarrheal diseases	89.34(41.45-128.01)	33.49(7.26-60.01)	97.4(44.17-157.09)	106.29(38.78-181.12)	110.43(47.86-192.69)	59.29(23.45-92.79)	88.53(30.51-144.95)	65.96(24.09-114.39)	77.84(38.48-122.86)	106.66(51.24-188.67)	95.42(45.47-137.08)	77.51(27.87-126.99)
	Ischemic heart disease	88.72(62.41-116.59)	135.31(95.32-172.79)	105.88(71.3-147.12)	96.89(63.78-135.1)	85.72(53.26-125.99)	89.99(58.09-129.33)	142.85(100.1-194.45)	97.65(65.89-133.44)	71.85(47.04-100.45)	92.57(59.73-133.74)	92.64(64.98-127.18)	99.4(64.61-135.93)
	Tuberculosis	76.46(61.34-93.14)	62.14(45.26-89.51)	136.28(100.82-180.56)	80.8(56.71-110.07)	94.59(67.04-128.4)	62.21(44.22-83.16)	125.02(94.38-160.1)	81.5(56.85-110.45)	62.46(48.47-80.16)	114.37(80.19-166.41)	87.4(67.77-110.63)	64.22(47.27-83.21)
	Cirrhosis and other CLD	67.77(54.36-84.71)	64.39(47.11-85.13)	67.69(47.91-96.86)	69.81(49.77-109.52)	61.27(41.97-95.02)	62.76(45.59-89.76)	85.23(63.09-110.6)	68.26(47.51-94.67)	62.08(47.46-79.96)	65.66(45.54-97.44)	83.29(63.9-105.55)	54.92(41.48-71.44)
	Neonatal disorders	52.71(40.9-68.5)	16.1(11.09-22.75)	44.45(33.87-59.47)	57.29(44.18-76.61)	64.17(48.66-86.03)	48.45(35.4-64.97)	37.86(27.9-50.97)	48.12(35.08-64.51)	52.87(41.23-68.44)	58.13(45.01-75.57)	53.22(40.71-70.05)	36.68(28.12-48.97)
	Diabetes mellitus	42.91(34.55-52.79)	52.16(38.03-66.76)	41.46(31.06-54.04)	40.78(30.07-54.27)	35.05(25.65-47.99)	42.99(32.07-56.64)	58.06(45.27-73.95)	47.66(34.6-62.99)	39.82(30.73-50.81)	37.28(26.72-50.43)	52.85(40.81-69.06)	44.74(34.01-57.75)
	Chronic obstructive pulmonary disease	37.23(27.45-47.01)	31.25(21.58-40.55)	34.26(21.06-52.36)	39.54(26.94-56.88)	30.45(18.09-48.03)	29.39(19.87-40.86)	43.72(29.8-59.1)	34.55(23.27-47.07)	34.14(24.57-44.04)	33.95(19.8-66.13)	42.49(30.37-55.67)	40.26(28.31-56.43)
	Chronic kidney disease	30.52(24.68-37.3)	31.53(22.68-40.73)	30.22(22.83-39.45)	29.39(21.35-38.88)	26.46(18.69-35.83)	30.69(23.02-40.35)	38.92(29.18-49.46)	34.09(25.47-44.98)	28.79(21.38-37.04)	27.97(20.07-37.89)	36.57(27.88-46.89)	31.6(22.98-40.67)
	Hypertensive heart disease	25.76(11.84-54.39)	30.92(13.4-52.74)	26.83(12.93-52.48)	27.35(11.19-57.97)	24.05(10.64-48.36)	26.43(12.28-52.06)	34.09(17.54-64.32)	29.5(13.73-53.34)	22.54(8.66-51.2)	25.98(11.52-54.62)	27.47(13.37-55.78)	29.27(12.05-64.52)

	Causes of death	Ethiopia	Addis Ababa	Afar	Amhara	B/Gumuz	Dire Dawa	Gambella	Harari	Oromia	Somali	SNNP	Tigray
	Alzheimer's disease and other dementias	25.49(6.12-72.01)	24.87(5.72-71.32)	22.9(5.34-64.22)	24.45(5.63-67.59)	22.7(5.32-64.39)	24.57(5.67-69.63)	24.44(5.63-69.92)	24.42(5.66-66.97)	26.36(6.32-72.44)	22.91(5.33-62.43)	26.62(6.19-72.11)	25.83(6-71.88)
	HIV/AIDS	25.4(20.93-30.96)	62.14(45.26-89.51)	48.16(31.55-70.79)	34.2(25.57-44.78)	21.95(12.86-36.41)	56.99(32.15-86.93)	58.41(42.26-89.85)	38.77(22.88-76.17)	16.9(10.64-25.06)	34.3(24.58-47.63)	15.27(9.85-22.29)	24.97(14.46-37.83)
	Road injuries	20.83(16.67-26.02)	20.03(13.98-26.5)	22.34(16.05-30.68)	20(14.72-26.67)	19.62(14.61-26.71)	21.82(13.19-36.55)	26.74(19.75-35.92)	22.25(14.52-42.38)	18.78(14.39-24.68)	20.54(15-28.14)	26.48(20.11-35.51)	19.18(14.05-26.25)
	Meningitis	15.43(12.72-18.73)	11.55(8.4-15.21)	20.19(14.85-27.24)	14.94(10.95-20.14)	17.23(13.11-22.46)	11.62(8.54-15.71)	18.45(13.61-23.94)	14.53(10.43-19.51)	13.78(10.71-17.58)	20.39(14.63-27.46)	18.15(14.02-22.86)	12.2(8.57-16.33)
	Self-harm	14.5(10.89-19.63)	15.07(10.63-21.6)	14.94(10.59-21.77)	14.25(9.73-22.46)	12.61(8.76-18.43)	12.18(8.45-18.42)	18.44(12.7-27.25)	13.79(9.47-21.16)	13.25(9.55-18.64)	13.43(9.28-19.52)	18.23(13.2-25.04)	12.69(8.29-20.7)
	Interpersonal violence	14.45(11.39-18.39)	11.91(8.14-16.93)	19.36(12.16-29.28)	14.89(10.36-21.03)	16.23(10.94-23.11)	12.85(8.55-19.23)	19(12.76-28.25)	13.85(8.76-20.32)	11.87(8.71-16.11)	17.41(11.21-25.53)	17.35(12.55-23.27)	15.29(10.9-21.37)
	Prostate cancer	13.83(7.51-22.04)	13.99(9.21-24.26)	10.68(6.04-16.39)	13.69(6.88-22.55)	11.1(5.56-18.46)	14.39(8.96-22.64)	15.04(10.62-22.31)	14.76(9.76-22.57)	13.87(6.62-22.46)	10.77(5.04-17.78)	14.68(8.41-23.19)	15.47(8.11-24.84)
	Protein-energy malnutrition	12.87(9.87-17.19)	12.86(9.19-16.93)	33.48(24.29-46.28)	4.73(2.93-10.23)	25.22(18.15-32.83)	13.43(8.69-22.91)	16.73(12.18-23.43)	12.46(8.14-20.46)	13.93(9.97-19.17)	29.72(20.7-43.45)	14.53(10.22-21.12)	11.24(7.82-16.85)
	Paralytic ileus and intestinal obstruction	11.51(8.4-16.56)	9.5(5.5-13.75)	10.86(6.99-17.19)	12.17(7.92-23.52)	10.95(7.09-19.16)	10.87(6.72-15.72)	12.57(8.46-17.35)	11.3(6.76-16.38)	10.15(7.17-14.42)	11.2(7.09-19.13)	14.35(9.29-18.98)	11.09(7.43-16.58)

Table S9. Age-standardised YLL rates per 100 000 population for all causes combined and leading fifteen causes in Ethiopia's regions and chartered cities, women and men, 2019
YLLs = years of life lost

	Causes of YLL	Ethiopia	Addis Ababa	Afar	Amhara	B/Gumuz	Dire Dawa	Gambella	Harari	Oromia	Somali	SNNP	Tigray
Female YLLs	All causes	26577.35(23737.66-29963.79)	22406.34(18156.65-27566.68)	45082.71(36941.74-54439.29)	24309.9(21344.28-28052.02)	42714.8(35285.33-51216.37)	24472.68(20774.4-29063.37)	23062.71(19659.37-26958.46)	24647.64(20438.86-29811.54)	25729.51(22546.55-29787.68)	35828.89(29916.14-42775.25)	27971(24105.43-32513.63)	23650.79(19714.42-28204.49)
	Neonatal disorders	3148.55(2515.94-3959.73)	845.69(569.77-1184.48)	2678.72(2097.97-3468.9)	3395.67(2668.31-4318.45)	3837.75(2990.51-4941.23)	2854.04(2152.98-3705.69)	2208.36(1672.4-2899.85)	2828.51(2092.64-3688.39)	3188.83(2548.29-4015.62)	3473.65(2778.67-4355.3)	3157.79(2486.5-4050.02)	2180.68(1706.41-2797.08)
	Diarrheal diseases	2321.88(1370.91-3911.37)	488.11(210.28-1202.45)	3147.85(1569.68-5463.39)	2395(1153.12-4816.22)	3322.39(1541.19-6372.74)	1319.21(593.17-2736.4)	1275.36(614.08-2347.17)	1435.04(651.44-2843.69)	2379.2(1348.64-4037.61)	2871.89(1452.32-5136.38)	2551.31(1411.14-4385.83)	1520.6(776.09-2856.77)
	Lower respiratory infections	2134.4(1740.34-2579.97)	1046.19(790.46-1410.55)	3054.65(2338.89-3934.38)	1700.11(1184.09-2282.95)	3737.87(2779.7-4868.22)	1547.03(1096.03-2134.76)	1187.88(842.38-1528.97)	1753.37(1294.38-2370.1)	2229.48(1787.05-2805.78)	3074.32(2310.11-3959.32)	2331.2(1821.45-2922.89)	1745.12(1338.15-2238.23)
	HIV/AIDS	1958.84(1578.86-2410.44)	5824.14(4215.45-7698.71)	3453.64(2290.68-4969.28)	2194.97(1493.34-2966.22)	1539.13(818.23-2696.06)	3862.23(2306.24-6102.92)	5929.51(3325.69-9305.26)	2124.49(1356.55-3218.93)	1482.6(1015.03-2120.63)	2476.2(1736.93-3439.63)	1181.33(772.06-1711.1)	1973.52(1100-2960.14)
	Stroke	1549.95(1188.54-1908.07)	1865.75(1407.33-2463.55)	3527.46(2600.12-4658.75)	1328.22(867.97-1799.31)	2943.71(2122.22-3911.56)	1418.44(981.27-1938.88)	1264.9(750.55-1753.67)	1645.74(1170.26-2210.21)	1420.12(1045.42-1835.18)	2155.22(1555.69-2878.22)	1554.69(1173.35-1993.9)	2037.08(1475.52-2694.31)
	Ischemic heart disease	1340.16(959.64-1698.12)	1686.6(1241.23-2262.52)	2989.17(2190.61-4095.93)	1101.42(707.03-1529.79)	2539.66(1828.7-3373.8)	1273.5(806.95-1767.24)	1127.4(639.42-1538.09)	1417.63(988.87-1898.24)	1309.83(875.52-1731.84)	1810.37(1277.27-2480)	1380.92(1003.59-1801.52)	1526.85(1017.9-2119.85)
	Tuberculosis	1202.54(930.48-1590.75)	791.41(534.48-1177.11)	4417.79(3125.46-6021.84)	1010.9(607.79-1616.5)	3303.63(2355.17-4443.97)	792.09(476.05-1149.52)	785.79(527.74-1056.19)	1069.01(532.39-1610.62)	1068.02(774.6-1525.65)	2671.86(1748.81-4700.12)	1279.33(936.28-1786.35)	915.56(639-1363.33)
	Maternal disorders	870.11(630.75-1186.06)	284.7(167.43-482.47)	2473.91(1568.46-3755.96)	560.35(345.66-840.3)	2223.66(1479.96-3070.27)	493.51(317.46-823.28)	302.64(186.91-446.7)	580.13(351.82-996.02)	870.93(573.24-1286.61)	1798.67(1135.6-2579.23)	938.37(603.7-1374.17)	828.59(518.14-1241.9)
	Cirrhosis and other chronic liver diseases	844.92(643.41-1081.32)	767.23(530.19-1111)	1477.11(1005.71-2111.13)	705.08(438.81-1094.11)	1268.13(823.35-1788.96)	742.46(487.63-1153.92)	616.51(374.23-967.24)	797.97(538.4-1177.01)	854.55(632.2-1108.8)	965.63(678.23-1334.07)	975.09(722.61-1286.58)	834.15(581.82-1151.11)
	Malaria	661.8(132.28-1729.36)	75.01(4.88-291.22)	388.58(32.36-1241.73)	619.21(75.96-1724.78)	1329.62(162.8-3889.59)	1500.31(174.59-3555.44)	1073.79(246.87-2660)	1354.94(187.95-3465.16)	628.85(109.15-1728.03)	634.96(76.66-1764.77)	836.64(186.45-2363.05)	480.01(66.43-1500.34)
	Hypertensive heart disease	615.33(344.14-975.83)	599.93(258.29-1046.83)	1236.02(622.82-1912.4)	538.24(281.05-969.91)	1027.97(523.23-1560.39)	558.14(249.52-1056.61)	501.45(250.79-1172.39)	630.54(274.1-1163.21)	603.45(319.28-947.26)	786.53(420.09-1264.21)	661.84(355.42-957.68)	676.24(363.84-1061.57)
	Congenital birth defects	606.05(365.52-984.56)	270.1(125.64-502.34)	497.05(289.37-830.67)	695.16(334.44-1304.29)	875.04(398.84-1634.63)	511.78(284.73-887.71)	356.98(211.42-594.68)	505.02(290.54-854.73)	570.35(336.97-957.47)	711.22(390.55-1284.29)	622.16(347.11-1101.79)	455.95(276.31-731.45)

	Causes of YLL	Ethiopia	Addis Ababa	Afar	Amhara	B/Gumuz	Dire Dawa	Gambella	Harari	Oromia	Somali	SNNP	Tigray
	Diabetes mellitus	559.48(460.23-657.75)	564.4(409.68-765.33)	1099.2(816.39-1427.18)	425.86(307.67-558.48)	947.03(692.62-1251.85)	479.39(341.43-641.5)	360.77(249.04-460.82)	541.34(390.53-737.61)	581.79(450.19-738.19)	680.47(495.44-920.26)	643.57(504.23-808.72)	598.37(445.45-781.74)
	Meningitis	536.36(428.35-669.49)	310.97(221.63-442.47)	1130.56(820.29-1495.09)	446.59(307.74-654.68)	1059.11(748.76-1417.27)	356.18(243.63-509.64)	285.66(202.13-414.96)	444.02(295.24-645.76)	506.49(378.55-677.64)	946.33(669.45-1321.31)	592.89(441.26-774.39)	378.53(273.67-518.53)
	Cervical cancer	489.24(337.23-795.31)	449.12(293.76-664.13)	1065.1(657.58-1551.95)	369.08(228.7-704.76)	871.23(535.28-1240.63)	370.92(246.31-610.4)	306.97(203.46-565.12)	421.69(268.74-646.41)	497.91(314.48-880.77)	638.55(392.84-1067.6)	560.52(361.66-898.57)	504.26(317.08-837.09)
	Protein-energy malnutrition	466.88(306.54-683.44)	196.2(144.48-262.1)	1030.89(652.86-1511.77)	285.2(64.04-693.92)	1141.64(745.78-1649.2)	331.33(184.68-542.16)	214.04(94.9-358.96)	328.79(170.54-565.35)	490.84(289.54-763.99)	1359.62(671.37-2322.56)	429.08(246.38-691.79)	251.69(152.82-386.79)
	Breast cancer	453.81(360.68-557.49)	788.38(522.16-1160.51)	715.81(503.68-1006.69)	325.87(213.22-517.15)	663.97(463.72-947.21)	461.65(328.44-684.08)	349.62(242.06-523.01)	516.12(354.4-748.03)	452.22(343.8-572.35)	380.76(239.35-623.46)	532.9(393.26-676.65)	463.27(316.12-655.03)
	Whooping cough	430.96(44.89-1217.46)	74.84(2.47-298.53)	659.95(30.18-2290.3)	465.57(19.61-1764.87)	498.17(14.66-2265.84)	219.03(8.87-989.3)	292.78(11.76-1097.14)	408.03(12.23-1589.82)	410.78(27.89-1267.09)	699.7(30.89-2582.5)	418.83(16.06-1646.67)	189.83(7.24-772.12)
	Chronic kidney disease	425.36(354.19-503.84)	277.1(100.25-398.32)	747.87(546.21-1025.87)	307.95(232.31-424.16)	682.5(489.02-934.19)	391.72(288.42-533.73)	273.01(214.33-377.31)	440.6(323.47-608.57)	454.6(352.43-552.98)	467.07(336.11-661.46)	506.68(396.63-624.49)	416.32(306.92-572.72)
	Chronic obstructive pulmonary disease	338.09(231.23-446.98)	55.64(38.66-79.93)	450.84(232.44-956.79)	320.67(210.93-500.49)	416.11(197.32-877.8)	200.19(118.93-317.31)	162.91(102.25-290.17)	256.54(154.7-367.31)	335.7(228.75-447.28)	337.76(174.18-657.04)	364.81(241.08-501.35)	379.08(232.17-549.52)
Male YLLs	All causes	33656.53(29329.57-38434.03)	27213.18(21615.54-33156.63)	38024.59(30891.2-46081.88)	34944.14(29673.75-40742.87)	38301.56(31537.43-45911.41)	31504.05(26574.2-37743.06)	40161.08(33217.41-48311.31)	33638.8(27653.79-40521.79)	30159.08(25750.84-35341.45)	39100.96(32276.84-46571.16)	37724.94(31927.22-44383.04)	29250.6(24341.36-34727.67)
	Neonatal disorders	4682.38(3633.09-6085.01)	1430.26(985.73-2021.49)	3948.63(3009.22-5283.55)	5088.34(3923.98-6804.45)	5700.11(4321.17-7641.59)	4304.31(3145.2-5772.5)	3363.55(2478.88-4529.27)	4275.07(3116.29-5732.05)	4697.04(3663.04-6079.78)	5163.87(3998.38-6714.02)	4727.98(3616.51-6223.29)	3259.17(2498.22-4351.09)
	Diarrheal diseases	3025.56(1811.69-4105.96)	950.49(308.5-1640.3)	3037.6(1665.27-4678.37)	3364.44(1571.97-5428.03)	3580.36(1885.73-5841.46)	1751.52(868.27-2704.4)	2293.72(1016.02-3551.67)	1991.36(960.55-3129.7)	2884.32(1769.56-4279.79)	3405.3(2025.35-5314.91)	3209.66(1935.99-4563.03)	2264.94(1098.16-3390.06)
	Lower respiratory infections	2664.11(2219.4-3258.11)	1564.82(1198.17-2064.01)	2679.49(2059.02-3452.98)	2346.51(1757.18-3136.8)	3431.02(2521.89-4594.11)	2139.78(1634.49-2791.32)	2731.26(2120.34-3413.15)	2417.27(1818.81-3169.53)	2622.97(2073.95-3281.37)	3332.03(2520.57-4287.02)	3059.89(2441.07-3791.68)	2220.79(1719.29-2794.87)
	Tuberculosis	2251.18(1806.41-2749.76)	1779.88(1304.02-2437.84)	4156.58(2951.01-5670.73)	2392.68(1679.45-3319.8)	2966.72(2131.16-4031.18)	1753.34(1233.41-2412.94)	3591.54(2603.4-4792.09)	2352.81(1627.98-3212.92)	1771.28(1368.8-2282.32)	3510.85(2484.87-5036.19)	2593.55(1998.93-3326.96)	1818.94(1331.07-2400.92)
	Cirrhosis and other chronic liver diseases	1804.71(1430.63-2302.66)	1739.01(1243.83-2364.8)	1892.92(1301.77-2823.22)	1836.64(1289.9-2998.4)	1697.07(1153.74-2690.87)	1626.37(1142.18-2415.62)	2264.16(1630.5-3005.34)	1790.5(1206.21-2554.03)	1633.51(1206.25-2115.94)	1794.7(1228.08-2745.58)	2241.64(1686.15-2866.89)	1419.93(1031.37-1929.97)
	Stroke	1728.49(1241.17-2251.22)	2329.5(1646.94-3020.93)	2181.21(1503.97-2948.8)	1847.02(1229.59-2602.38)	1745.62(1115.99-2526.67)	1741.3(1165.67-2451.38)	2961.1(2059.18-3962.38)	2061.13(1385.18-2857.7)	1353.04(894.72-1863.23)	1979.69(1310.41-2813.47)	1836.74(1292.24-2479.74)	2092.17(1414.39-2834.27)

	Causes of YLL	Ethiopia	Addis Ababa	Afar	Amhara	B/Gumuz	Dire Dawa	Gambella	Harari	Oromia	Somali	SNNP	Tigray
	Ischemic heart disease	1706.61(1192.62-2270.82)	2620.28(1821.43-3428.99)	2142.03(1446.55-3057.05)	1837.38(1183.31-2613.28)	1681.97(1034.33-2517.89)	1665.25(1048.82-2446.4)	2872.96(1953.81-3993.65)	1862.4(1238.14-2606.79)	1337.48(867.41-1898.39)	1820.8(1160.19-2655.37)	1851.36(1286.26-2565.1)	1860.4(1177.85-2618.59)
	HIV/AIDS	1197.32(977.12-1503.43)	2860.18(2090.46-4049.43)	2212.1(1441.51-3285.91)	1637.83(1216.13-2146.01)	1118.4(649.87-1856.06)	2876.88(1703.04-4341.24)	3010.49(2158.53-4559.08)	2088.36(1195.27-3874.96)	830.63(537.47-1258.84)	1592.43(1099.36-2247.18)	719.67(449.97-1065.04)	1179.29(652.42-1832.12)
	Congenital birth defects	859.32(486.01-1440.44)	318.41(156.82-626.32)	663.56(380.05-1116.94)	1029.22(497.97-1792.39)	1164.71(536.84-2046.96)	711.94(407.46-1166.35)	527.06(332.78-829.87)	704.95(398.78-1171.16)	803.36(466.08-1376.69)	1003.66(482.15-1711.62)	863.26(449.03-1490.38)	645.1(397.43-992.97)
	Diabetes mellitus	846.38(679.54-1043.36)	1030.07(738.34-1366.95)	854.19(623.77-1128.5)	798.14(583.25-1074.09)	711.9(505.2-1000.09)	816.96(599.2-1109.51)	1183.11(883.3-1533.67)	931.89(677.21-1254.05)	763.6(587.32-981.7)	758.47(539.56-1038.67)	1068.39(808.35-1399.24)	862.85(645.72-1120.5)
	Road injuries	749(600.42-939.13)	695.67(491.98-949.94)	833.6(573.91-1182.21)	717.81(540.81-955.11)	793.27(595.75-1057.79)	748.64(449.97-1239.03)	926.36(668.52-1272.24)	779.25(496.65-1476.92)	680.08(117.89-1924.22)	784.97(575.7-1078.84)	958.74(727.81-1295.06)	660.74(485.09-925.27)
	Malaria	703.9(134.7-1873.08)	86.93(4.62-372.32)	364.63(30.35-1148.23)	666.7(80.36-1774.44)	1324.42(152.65-3826.95)	1634.74(177.07-3979.14)	1215.64(262.28-3063.17)	1420.69(210.17-3595.05)	680.08(117.89-1924.22)	632.53(76.45-1741.94)	886.8(194.34-2654.64)	518.77(65.58-1650.59)
	Meningitis	683.37(556.69-842.07)	410.88(298.05-560.18)	852.26(622.25-1163.27)	626.28(466.37-826.02)	901.69(663.65-1206.68)	491.16(350.83-691.99)	691.89(502.55-919.16)	608.34(427.59-830.4)	620.1(472.96-804.25)	984.46(730.1-1324.73)	806.79(623.93-1033.79)	465.64(339.56-627.05)
	Interpersonal violence	625.61(495.71-794.8)	531(355.18-773.73)	870.01(533.21-1347.84)	635.64(442.84-898.88)	734.56(501.23-1036.96)	552.43(358.97-828.32)	813.35(526.23-1233.34)	595.66(370.01-883.93)	514.63(377.14-706.59)	768.96(502.3-1133.53)	747.7(543.47-1015.77)	651.95(458.51-921.14)
	Chronic obstructive pulmonary disease	622.52(452.07-802.81)	527.5(365.06-696.7)	586.62(359.04-923.57)	660.62(429.85-988.3)	512.01(297.02-829.87)	475.64(317-681.16)	754.35(511.05-1034.17)	573.89(377.13-796.41)	556.21(393.6-739.41)	574.79(328.01-1175.1)	731.22(507.64-980.09)	666.46(451.77-956.9)
	Chronic kidney disease	614.99(503.4-754.81)	636.63(465.16-828.23)	638.54(476.47-835.13)	587.56(422.19-784.42)	565.15(400.4-773.66)	596.8(443.17-797.34)	794.3(585.45-1039.93)	679.13(499.6-915.59)	563.26(428.67-724.42)	584.38(409.61-808.42)	758.71(578.93-976.18)	617.34(451.62-799.22)
	Self-harm	467.22(347.56-620.06)	500.79(345.46-734.78)	510.65(344.94-742.64)	449.92(304.24-704.81)	431.45(300.62-616.18)	381.25(262.75-579.65)	597.85(393.79-900.46)	443.59(297.67-666.93)	416.89(293.5-575.56)	445.74(308.88-627.14)	599.95(431.96-796.88)	402.77(264.43-649.46)
	Hypertensive heart disease	463.51(210.4-971.39)	553.77(240.32-974.1)	501.27(241.63-988.92)	487.5(193.6-1048.26)	439.79(193.66-898.45)	463.16(211.79-903.69)	635.09(323.36-1206.21)	527.81(242.86-952.33)	398.22(150.21-931.14)	476.78(210.46-1040.72)	503.8(244.09-1042.08)	515.21(208.54-1174.33)
	Protein-energy malnutrition	426.48(318.27-595.75)	266.9(191.65-353.5)	851.03(604.72-1184.1)	182.71(98.05-371.9)	960.06(634.55-1426.32)	349.96(204.64-646.63)	372.35(267.44-547.31)	319.48(192.7-564.42)	465.7(318.65-687.01)	962.59(638.38-1464.1)	443.78(296.98-699.74)	264.78(182.69-402.11)
	Whooping cough	369.4(39.15-1054.07)	64.23(1.77-260.38)	556.51(22.57-2066.22)	417.53(16.2-1591.76)	410.61(10.73-1875.28)	196.21(7.89-909.65)	272.25(10.58-1063.16)	362.67(9.84-1596.67)	346.01(22.15-1136.36)	592.82(24.75-2223.26)	353.38(13.89-1457.77)	159.35(5.74-659.4)

Table S10. Age-standardised YLDs rates per 100 000 population for all causes combined and leading fifteen causes in Ethiopia’s regions and chartered cities, women, men, 2019
YLDs = years lived with disability

	Causes of YLDs	Ethiopia	Addis Ababa	Afar	Amhara	B/Gumuz	Dire Dawa	Gambella	Harari	Oromia	Somali	SNNP	Tigray
Female YLDs	All causes	11382.48(8537.97-14723.99)	11055.27(8288.81-14217.11)	12125.07(9023.58-15673.76)	11175.31(8326.77-14595.53)	11527.37(8635.24-14939.59)	11753.92(8818.05-15166.15)	11861.81(8936.95-15380.08)	11596.95(8665.25-14917.64)	11374.66(8486.34-14721.09)	12660.26(9472.6-16439.19)	11371.26(8499.87-14701.67)	11060.79(8342.68-14331.51)
	Gynecological diseases	937.02(642.5-1312.53)	918.8(624.96-1294.82)	955.65(660.15-1348.46)	928.48(630.7-1301.29)	930.44(634-1301.9)	936.74(641.82-1318.45)	931.95(634.96-1307.48)	943.22(647.53-1324.24)	938.51(642.62-1314.15)	988.34(680.57-1372.16)	935.48(643.78-1315.47)	929.52(632.56-1296.85)
	Depressive disorders	876.14(607.07-1200.73)	916.24(636.27-1255.78)	852.18(592.17-1181.42)	874.95(604.9-1200.22)	861.44(601.17-1188.5)	865.97(603.95-1191.27)	864.72(601.48-1189.01)	867.43(602.3-1195.76)	880.88(604.99-1211.79)	841.97(585.54-1159.84)	871.94(608.29-1199.31)	869.54(610.51-1200.61)
	Low back pain	670.17(474.44-901.3)	610.38(431.4-815.61)	645.72(454.55-869.53)	664.55(469.47-884.29)	668.49(468.35-901.15)	621.55(438.71-829.77)	670.45(475.74-906.56)	620.65(438.08-831.55)	683.47(483.4-921.45)	649.04(458.05-877.26)	678.98(481.97-901.61)	667.66(469.41-904.11)
	Age-related and other hearing loss	533.56(367.08-750.82)	480.5(327.01-682.39)	548.22(376.41-773.21)	539.28(369.02-752.63)	535.05(367.46-752.65)	503.75(344.34-704.76)	517.58(354.58-730.11)	504.17(345.13-703.92)	536.02(366.36-750.36)	558.39(385.62-775.93)	529.69(361.56-737.69)	527.87(361.94-739.36)
	Blindness and vision loss	460.45(331.27-616)	403.88(287.54-548.44)	393.28(282.66-538.97)	438.74(314.83-592.04)	377.07(271.74-514.48)	505.52(360.74-677.85)	483.33(347.43-651.86)	524.75(377.28-699.71)	430.31(115.55-892.03)	390.69(280.76-528.2)	616.96(440.03-829.3)	404.81(286.89-550.62)
	Headache disorders	428.86(117.86-896.33)	426.05(116.88-897)	444.19(127.36-898.29)	430.17(117.19-898.85)	445.93(126.44-920.54)	445.79(127.66-922.1)	444.82(127.73-924.4)	445.49(125.8-923.36)	430.73(308.99-579.99)	442.53(126.45-911.67)	413.04(116.25-864.73)	446.98(128.18-935.73)
	Dietary iron deficiency	422.68(281.03-611)	350.86(215.03-535.74)	715.33(470.55-1066.79)	282.54(178.89-424.94)	324.08(205.82-490.27)	678.41(429.33-995.17)	412.14(248.05-621.67)	480.73(298.53-717.71)	422.87(273.58-626.97)	1168.84(790.81-1671.83)	368.03(236.25-554.69)	342.33(210.91-515.64)
	Anxiety disorders	403.21(281.23-552.51)	404.4(283.99-559.84)	403.68(279.87-561.64)	402.96(277.71-555.64)	401.66(277.35-558.7)	403.63(280.09-551.56)	402.92(275.61-555.76)	402.96(276.03-550.8)	403.47(279.41-553.15)	401.69(282.15-552.95)	402.62(282.61-550.75)	404.68(283.62-568.33)
	Neonatal disorders	296.71(226.62-374.97)	402.75(313.39-508.02)	314.7(236.49-397.52)	301.29(227.66-389.19)	250.4(183.01-324.58)	326.26(248.93-417.8)	327.27(249.11-411.58)	310.51(233.29-394.34)	299.99(225.03-382.64)	317.35(244.01-400.1)	250.44(181.41-331.08)	329.58(255.4-418.33)
	Endocrine, metabolic, blood, and immune disorders	286.75(192.59-401.36)	318.76(211.34-451.67)	303.95(204.52-423.37)	276.52(184.25-392.69)	274.36(184.71-391.15)	326.91(217.21-458.86)	288.3(190.89-405.24)	311.82(209.69-441.6)	284.41(190.49-405.25)	332.83(224.43-463.63)	280.05(187.08-401.02)	282.06(188.28-396.03)
	Diabetes mellitus	261.61(180.32-360.2)	288.75(195.41-394.88)	324.29(221.35-443.78)	228.22(155.96-313.83)	323.22(220.09-436.84)	260.86(177.36-359.58)	236.78(161.84-324.27)	285.98(191.4-394.51)	252.17(136.35-422.88)	292.06(201.72-403.65)	280.11(191.42-384.21)	272.51(186.63-375.76)
	Oral disorders	252.28(135.77-422.93)	253.22(134.69-434.24)	250.74(136.58-421.79)	252.75(136.72-423.71)	251.46(135.77-422.33)	252.29(132.91-427.44)	251.85(134.97-422.91)	252.34(134.1-425.91)	252.17(136.35-422.88)	250.56(135.88-415.86)	252.14(137.56-425.66)	252.16(135.44-422.43)

	Causes of YLDs	Ethiopia	Addis Ababa	Afar	Amhara	B/Gumuz	Dire Dawa	Gambella	Harari	Oromia	Somali	SNNP	Tigray
	Other musculoskeletal disorders	251.86(155.73-361.59)	323.12(201.14-465.18)	240.29(148.11-348.95)	243.84(144.01-356.41)	253.26(160.07-366.83)	276.43(171.66-402.24)	239.43(140.54-357.84)	249.27(139.68-377.28)	252.99(158.71-368.33)	219.68(133.82-322.98)	251.51(149.94-364.58)	239.56(138.56-358.86)
	Osteoarthritis	210.2(107.05-410.19)	224.16(113.43-444.55)	208.58(105.74-408.88)	209.52(105.72-413.28)	209.02(105.44-409.35)	216.68(109.85-425.61)	211.35(107.22-417.6)	215.78(108.62-424.27)	209.38(106.9-414.66)	207.09(104.32-412.58)	208.73(106.33-409.52)	211.29(107.16-415.59)
	Diarrheal diseases	206.87(143.22-286.77)	167.07(114-230.52)	202.73(139.58-282.51)	202.77(140.57-282.78)	246.98(169.23-342.5)	179.09(123.43-247.02)	216.99(148.71-299.52)	200.79(138.17-277.23)	210.63(145.06-292.03)	217.36(149.38-299.66)	215.46(148.52-299.17)	187.56(129.36-262.76)
	Schistosomiasis	167.61(86.66-307.59)	0(0-0)	65.03(36.69-104.29)	244.52(122.95-460.12)	23.66(11.92-43)	106.18(57.15-188.45)	21.26(11.21-38.35)	0(0-0)	155.11(80.52-286.17)	49.6(29.62-77.54)	192.4(99.68-352.41)	158.67(80.48-290.02)
	Stroke	162.88(116.71-208.92)	248.01(176.92-321.06)	211.68(152.9-270.83)	151.16(107.65-193.51)	206.47(148.68-264.66)	195.56(138.41-251.49)	178.37(129.1-227.5)	209.87(152.17-270.08)	158.68(113.92-202.66)	186.98(135.48-240.41)	147.93(106.74-189.84)	179.18(128.3-230.88)
	Chronic obstructive pulmonary disease	151.09(123.1-177.92)	188.54(153.53-223.58)	181.48(148.29-215.08)	134.31(108.37-159.73)	183.4(149.71-217.96)	218.14(177.62-256.85)	181.88(149.98-213.58)	240.51(196.93-283.87)	148.3(119.4-176.39)	159.52(128.41-189.35)	159.95(127.65-190.39)	156.42(126.69-185.52)
	HIV/AIDS	150.26(99.38-216.86)	457.01(289.67-677.42)	226.78(129.17-378.78)	179.14(114.75-273.77)	133.11(77.18-220.11)	340.71(201.84-548.76)	509.28(293.82-832.48)	280.87(165.97-433.2)	111.86(69.31-179.19)	116.81(60.64-219.27)	86.01(50.6-137.57)	163.64(95.66-248.21)
	Neck pain	144.92(96.17-208.83)	145.27(95.88-209.07)	144.56(95.17-209.1)	144.96(95.38-209.13)	144.84(95.47-207.48)	144.82(94.47-209.26)	144.56(95.56-208.44)	144.75(94.72-208.69)	144.88(95.17-207.27)	144.28(95.01-207.88)	144.91(95.67-209.24)	145.09(95.74-210.09)
Male YLDs	All causes	10216.18(7567.67-13239.59)	9733.58(7310.32-12486.45)	10564.76(7902.97-13664.01)	10159.72(7543.61-13229.83)	10080.18(7549.55-13018.51)	10341.9(7684.04-13313.56)	10751.49(8052.45-13883.68)	10302.94(7651.18-13289.89)	10207.93(7554.02-13253.29)	10526.65(7856.67-13631.36)	10235.5(7624.2-13303.87)	10180.33(7611.62-13237.34)
	Depressive disorders	741.81(512.52-1026.44)	700.7(480.68-975.73)	729.47(505.54-1017.69)	742.96(510.27-1030.21)	734.51(507.26-1014.71)	738.25(511.34-1019.66)	734.69(505.63-1011.85)	739.69(516-1030.27)	751.31(518.31-1038.79)	723.37(498.24-1000.78)	741.38(512.52-1021.5)	737.41(511.31-1016.83)
	Low back pain	730.24(513.7-979.26)	665.64(465.54-897.5)	706.79(496.78-950.63)	723.18(513.03-978.85)	729.94(510.42-985.79)	678.75(473.89-914.66)	726.82(516.37-972.22)	676.47(476.45-913.36)	744.64(519.57-1002.29)	710.84(496.12-962.81)	739.37(516.13-1000.25)	726.38(515.39-982.37)
	Age-related and other hearing loss	571.04(394.85-807.16)	517.47(352.99-731.13)	584.61(400.31-821.36)	574.44(396.98-807.08)	571.12(390.34-807.11)	542.74(368.32-765.14)	551.25(373.87-772.34)	540.45(369.31-762.93)	573.39(395.85-809.99)	595.35(412.07-839.43)	566.7(389.42-796.4)	565.53(391.24-792.69)
	Alcohol use disorders	454.28(304.77-644.73)	486.49(326.32-687.91)	460.66(305.57-646.77)	453.56(299.91-642.47)	454.53(302.95-646.31)	462.37(310.01-651.64)	471.21(317.44-678.52)	472.05(316.66-663.48)	446.17(296.36-631.75)	450.36(299.67-639.47)	460.92(308.39-656.57)	455.11(305.64-648.19)
	Blindness and vision loss	383.02(272.55-519.95)	347.22(246.22-474.95)	322.34(228.23-445.97)	355.48(251.77-483.78)	313.58(223.53-430.77)	410.54(292-556.93)	379.93(271.01-517.27)	428.49(306.6-582.75)	369.14(264.12-501.8)	347.36(248.19-478.52)	505.96(361.64-683.24)	340.33(239.33-467.52)
	Dietary iron deficiency	370.51(243.97-548.63)	184.58(113.75-280.55)	651.89(415.68-992.99)	275.52(171.3-429.89)	271.12(164.3-412.23)	635.62(412.52-925.11)	352.53(206.6-549.4)	513.32(325.35-768.71)	418.16(261.33-627.49)	651.12(410.67-982.35)	269.64(166.49-414.45)	384.24(233.2-584.22)

	Causes of YLDs	Ethiopia	Addis Ababa	Afar	Amhara	B/Gumuz	Dire Dawa	Gambella	Harari	Oromia	Somali	SNNP	Tigray
	Neonatal disorders	318.26(242.74-401.72)	430.65(332.45-537.78)	369.73(282.95-465.34)	291.19(215.26-379.78)	238.95(173.04-321.49)	314.68(230.77-417.11)	337.21(256.12-425.91)	311.39(229.45-406.47)	332.01(246.72-426.18)	276.19(206.47-357.75)	307.44(230.19-401.02)	352.41(267.68-442.89)
	Diabetes mellitus	284.78(194.89-390.61)	323.18(220.05-439.75)	320.29(219.37-440.03)	276.45(187.92-379.25)	287.19(194.82-392.53)	284.81(191.18-394.94)	338.26(231.61-460.52)	306.48(206.48-418.28)	267.4(182.21-367.3)	289.49(197.2-402.68)	322.02(220.05-437.34)	277.65(187.5-380.72)
	Conflict and terrorism	279(154.19-557.77)	271.33(160.72-489.96)	214.6(114.68-427.4)	340.17(203.97-576.85)	246.01(131.92-482.48)	245.27(135.49-457.06)	543.22(340.76-857.62)	463.53(286.01-729.05)	236.68(129.29-451.27)	287.43(166.44-480.22)	214(73.76-804.95)	451.64(280.24-728.71)
	Headache disorders	277.63(74.61-577.65)	277.4(72.53-580.86)	291.8(80.59-608.79)	277.07(73.01-575.58)	291.88(82.74-606.93)	292.21(81.59-604.75)	290.85(79.82-602.06)	292.28(83.92-609.08)	277.11(73.73-579.11)	292.02(81.97-610.96)	267.41(71.08-562.47)	291.79(80.39-609.75)
	Road injuries	264.25(192.2-349.48)	263.03(189.93-349.3)	260.31(189.4-342.81)	257.79(186.34-340.94)	263.3(192.43-347.29)	281.43(205.23-371.12)	258.12(187.77-340.27)	273.03(199.71-361.7)	257.38(186.46-338.68)	288.74(210.83-381.63)	281.26(203.17-369.49)	253.73(185.36-337.02)
	Anxiety disorders	259.64(181.19-358.14)	260.58(181.6-359.85)	260.1(176.77-361.51)	258.81(177.21-361.34)	259.39(180.4-361.11)	260.13(180.85-359.33)	258.77(179.33-361.13)	258.98(181.02-360.98)	260.4(182.33-362.42)	259.09(180.43-362.33)	259.27(178.27-359.15)	259.21(180.01-365.23)
	Oral disorders	240.24(125.1-412.26)	243.1(125.36-426.46)	239.19(125.12-408.15)	240.25(127.14-411.69)	240.14(127.71-409.67)	241.54(125.84-419.84)	240.22(123.37-410.29)	242.02(126.91-423.99)	240.28(125.19-411.68)	240.17(124.28-410.97)	239.23(123.05-409.17)	240.57(124.71-408.85)
	Diarrheal diseases	230.45(158.63-317.86)	187.01(129.12-260.69)	215.15(149.67-296.93)	244.69(169.1-341.01)	255.01(176.55-353.67)	185.55(127.59-253.88)	224.05(153.32-309.95)	198.81(139.29-273.04)	221.63(153.86-306.26)	240.43(162.76-330.26)	238.45(163.18-328.45)	222.57(155.64-308.61)
	Osteoarthritis	180.31(91.58-354.62)	194.19(98.37-385.27)	179.3(90.36-351.08)	179.44(91.82-351.1)	179.81(91.15-352.96)	186.75(94.69-364)	181.6(91.03-357.82)	186.61(94.23-365.53)	180.08(90.95-353.79)	178.06(90.14-350.09)	178.81(90.5-355.07)	181.25(91.05-353.8)
	Endocrine, metabolic, blood, and immune disorders	171.98(115.02-240.15)	186.08(122.85-262.29)	183.8(124.59-258.24)	166.09(111.22-234.1)	165.77(111.59-234.31)	199.23(133.65-277.86)	171.44(115.46-238.11)	191.04(127.37-269.31)	173.54(116.31-245.43)	182.99(123.69-257.43)	165.33(110.41-233.3)	172.97(116.92-243.93)
	Schistosomiasis	162.51(76.51-314.62)	0 (0-0)	37.53(16.95-71.47)	253.12(116.39-498.89)	20.3(8.69-41.57)	92.84(43.27-178.47)	22.68(10.18-45.5)	0(0-0)	146.58(68.57-276.04)	10.59(4.85-20.22)	191.13(89.61-382.07)	169.28(79.35-326.74)
	Schizophrenia	142.67(103.56-182.86)	146.7(106.39-190.52)	140.37(99.49-181.62)	142.78(103.39-183.6)	141.49(101.88-181.07)	145.61(105.54-186.18)	140.44(100.23-178.96)	144.25(103.08-183.88)	143.53(103.84-184.38)	144.19(103.21-184.21)	139.8(101.05-180.04)	142.72(102.54-184.03)
	Fungal skin diseases	139.06(56.92-293.08)	136.57(55.29-292.64)	136.5(55.61-285.27)	138.56(56.03-290.98)	135.4(54.82-283.39)	137.13(55.27-284.81)	136.76(56.18-285.58)	140.6(56.5-294.31)	146.87(59.86-313.42)	131.66(52.99-277.51)	132.69(53.51-282.29)	129.54(52.04-275.35)
	Chronic obstructive pulmonary disease	138.74(114.53-162.39)	167.73(137.81-197.73)	137.58(111.99-162.58)	141.76(116.31-166.31)	135.41(110.05-159.3)	124.37(101.25-148.13)	147.13(121.45-173.17)	138.4(112.54-162.92)	126.14(103.37-151.04)	136.48(110.97-161.34)	151.56(122.81-180.25)	142.77(116.7-170.3)

Table S11. Age-standardised DALY rates per 100 000 populations for leading causes in Ethiopia's 9 regions and 2 cities in 1990, 2019 and percentage changes for both sexes

**Diseases and conditions**		**Ethiopia**	**Addis Ababa**	**Afar**	**Amhara**	**B/Gumuz**	**Dire Dawa**	**Gambella**	**Harari**	**Oromia**	**Somali**	**SNNP**	**Tigray**
Maternal disorders	1990	2215	1099	5325	2093	5665	1349	1056	1678	2215	3194	1826	2812
	2019	497	167	1298	339	1159	296	200	328	496	972	538	470
	% change	-78%	-85%	-76%	-84%	-80%	-78%	-81%	-80%	-78%	-70%	-71%	-83%
Neonatal disorders	1990	7009	4321	5807	7183	7924	7575	7379	7343	6948	5583	7785	6647
	2019	4244	1561	3678	4560	5039	3919	3135	3882	4279	4641	4242	3075
	% change	-39%	-64%	-37%	-37%	-36%	-48%	-58%	-47%	-38%	-17%	-46%	-54%
Diarrheal diseases	1990	11579	5528	18433	11927	17118	9990	13568	12480	12330	9998	12009	8277
	2019	2898	884	3238	3096	3719	1709	1986	1897	2853	3419	3110	2090
	% change	-75%	-84%	-82%	-74%	-78%	-83%	-85%	-85%	-77%	-66%	-74%	-75%
Tuberculosis	1990	10505	8581	20904	9513	18556	9343	11692	12954	11822	8369	9456	10117
	2019	1853	1366	4394	1828	3282	1366	2231	1828	1536	3281	2060	1459
	% change	-82%	-84%	-79%	-81%	-82%	-85%	-81%	-86%	-87%	-61%	-78%	-86%
Lower respiratory infection	1990	10208	6377	11014	8708	14986	11767	13194	12844	11237	6302	11651	10365
	2019	2416	1295	2837	2027	3583	1842	1947	2070	2445	3249	2709	1989
	%change	-76%	-80%	-74%	-77%	-76%	-84%	-85%	-84%	-78%	-48%	-77%	-81%
Protein-energy malnutrition	1990	4003	1642	7955	2935	7097	3593	3649	3193	4868	4273	4085	4415
	2019	492	260	998	281	1089	377	343	355	518	1214	481	293
	% change	-88%	-84%	-87%	-90%	-85%	-90%	-91%	-89%	-89%	-72%	-88%	-93%
HIV/AIDS	2005	8330	24442	9739	12370	6744	14234	15192	9697	6258	5404	3672	8756
	2019	372	4741	3010	2064	1427	3639	4982	2326	1242	2118	1023	1723
	% change	-80%	-81%	-69%	-83%	-79%	-74%	-67%	-76%	-80%	-61%	-72%	-80%

Table S12. Age-standardised DALY rates per 100 000 populations for all causes combined and leading fifteen causes in Ethiopia's regions and chartered cities, women, men, 2019
DALY = disability-adjusted life year

	**Causes of DALYs**	**Ethiopia**	**Addis Ababa**	**Afar**	**Amhara**	**B/Gumuz**	**Dire Dawa**	**Gambella**	**Harari**	**Oromia**	**Somali**	**SNNP**	**Tigray**
Female DALYs	All causes	37960	33462	57208	35485	54242	36227	34925	36245	37104	48489	39342	34712
	Neonatal disorders	3445	1248	2993	3697	4088	3180	2536	3139	3489	3791	3408	2510
	Diarrheal diseases	2529	655	3351	2598	3569	1498	1492	1636	2590	3089	2767	1708
	Lower respiratory infections	2144	1054	3066	1709	3750	1555	1196	1763	2240	3085	2341	1756
	HIV/AIDS	2109	6281	3680	2374	1672	4203	6439	2405	1594	2593	1267	2137
	Stroke	1713	2114	3739	1479	3150	1614	1443	1856	1579	2342	1703	2216
	Ischemic heart disease	1370	1721	3019	1131	2569	1305	1160	1450	1340	1840	1408	1557
	Tuberculosis	1321	894	4591	1153	3482	886	891	1177	1169	2843	1398	997
	Maternal disorders	982	312	2606	668	2355	592	378	677	992	1996	1050	905
	Gynecological diseases	943	922	967	933	940	940	935	947	945	999	942	935
	Depressive disorders	876	916	852	875	861	866	865	867	881	842	872	870
	Cirrhosis and other CLD	854	777	1486	713	1278	750	624	805	863	975	984	843
	Diabetes mellitus	821	853	1423	654	1270	740	598	827	848	973	924	871
	Malaria	732	92	481	662	1446	1585	1231	1453	709	769	910	530
	Congenital birth defects	699	343	608	788	979	594	445	591	661	819	716	544
	Low back pain	670	610	646	665	668	622	670	621	683	649	679	668
Male DALYs	All causes	43873	36947	48589	45104	48382	41846	50913	43942	40367	49628	47960	39431
	Neonatal disorders	5001	1861	4318	5380	5939	4619	3701	4586	5029	5440	5035	3612
	Diarrheal diseases	3256	1138	3253	3609	3835	1937	2518	2190	3106	3646	3448	2488
	Lower respiratory infections	2676	1575	2693	2358	3443	2151	2743	2427	2635	3345	3072	2234
	Tuberculosis	2380	1895	4322	2518	3109	1861	3733	2483	1892	3674	2729	1951
	Stroke	1843	2500	2312	1960	1867	1876	3104	2203	1463	2103	1943	2210
	Cirrhosis and other chronic liver diseases	1816	1751	1904	1848	1708	1637	2276	1801	1644	1806	2254	1430
	Ischemic heart disease	1786	2713	2223	1916	1764	1749	2956	1948	1418	1904	1924	1941
	HIV/AIDS	1282	3116	2349	1742	1195	3075	3282	2257	894	1663	770	1274
	Diabetes mellitus	1131	1353	1174	1075	999	1102	1521	1238	1031	1048	1390	1141
	Road injuries	1013	959	1094	976	1057	1030	1184	1052	917	1074	1240	914
	Congenital birth defects	954	390	772	1125	1266	795	619	791	896	1113	960	735
	Chronic obstructive pulmonary disease	761	695	724	802	647	600	901	712	682	711	883	809
	Malaria	745	101	414	696	1397	1684	1311	1478	725	690	929	555
	Depressive disorders	742	701	729	743	735	738	735	740	751	723	741	737
	Low back pain	730	666	707	723	730	679	727	676	745	711	739	726

Table S13. Patterns of age-standardised leading causes of premature mortality in Ethiopia and regions and chartered cities, 2019

	Ethiopia	Addis Ababa	Afar	Amhara	B-Gumuz	Dire Dawa	Gambella	Harari	Oromia	Somali	SNNP	Tigray
Neonatal disorders	1	7	2	1	1	1	2	1	1	1	1	1
Diarrheal diseases	2	9	3	2	3	6	7	6	2	3	2	4
Lower respiratory infections	3	4	5	3	2	3	6	3	3	2	3	3
Tuberculosis	4	5	1	5	4	8	3	5	4	4	4	7
Stroke	5	3	6	6	5	4	4	4	5	5	5	2
HIV/AIDS	6	1	4	4	9	2	1	2	8	6	8	6
Ischemic heart disease	7	2	7	7	6	7	5	7	6	7	6	5
Cirrhosis and other CLD	8	6	8	8	7	9	8	9	7	8	7	8
Congenital birth defects	9	20	18	9	12	11	18	11	9	12	11	11
Diabetes mellitus	10	8	11	11	14	10	10	10	10	13	10	9
Malaria	11	40	22	10	8	5	9	8	11	15	9	14
Meningitis	12	15	10	12	13	15	15	14	12	10	12	17
Hypertensive heart disease	13	10	13	13	15	12	11	12	14	17	15	10
Chronic kidney disease	14	11	14	16	17	13	12	13	13	18	13	13
Road injuries	15	13	15	15	16	14	13	15	16	16	14	15
Chronic obstructive pulmonary disease	16	14	19	14	19	18	17	16	17	20	16	12
Protein-energy malnutrition	17	24	12	24	11	17	20	20	15	9	20	22
Maternal disorders	18	27	9	21	10	23	33	23	18	11	18	16
Interpersonal violence	19	16	17	19	18	16	16	17	20	19	17	18
Whooping cough	20	48	16	17	20	25	21	18	19	14	21	31
Self-harm	21	19	20	23	23	24	19	22	22	21	19	20
Alzheimer's disease and other dementias	22	21	28	22	26	20	22	24	21	27	24	19
Leukemia	23	22	26	20	22	22	23	25	23	22	23	24
Breast cancer	24	12	24	28	24	21	25	21	25	33	25	21
Cervical cancer	25	23	21	27	21	28	32	27	24	25	26	23

Table S14. Burden of leading risk factors attributable to age-standardised YLL, YLD and DALYs rates in Ethiopia, both sexes, 2019
SEV= summary exposure value, YLL= years of life lost, YLD= years lived with disability, DALYs=disability-adjusted life years

Risk Factors	SEV per 100	YLL per 100,000	YLD per 100,000	DALYs per 100,000
Unsafe water, sanitation and hand washing	97	3142	213	3355
Air pollution	69	4072	374	4447
Dietary risks	38	1782	154	1936
Child and maternal malnutrition	18	5835	619	6454
High systolic blood pressure	20	2281	170	2451
High LDI cholesterol	14	464	31	497
High body mass index	11	884	186	1071
Tobacco use	5	564	84	648
High fasting plasma glucose	6	1385	334	1719
Unsafe sex	-	1563	134	1698
Alcohol Use	4	1176	308	1485

Table S15. Risk factors attributable age-standardised DALY rates per 100 000 population for all risks combined and leading fifteen risk factors, in Ethiopia 9 regions and 2 cities, females, males, and both sexes, 2019
DALY=disability-adjusted life-year

		Ethiopia	Addis Ababa	Afar	Amhara	B/Gumuz	Dawa	Gambella	Harari	Oromia	Somali	SNNP	Tigray
Risk factor-attributable DALYs, both sexes	All risk factors	20093	17745	26563	19175	24693	18860	22056	18821	19306	25299	20749	17834
	Particulate matter pollution	4434	2223	5776	4227	5793	3298	4144	3492	4306	5788	4757	4136
	Low birth weight and short gestation	3680	1338	3360	3701	4176	3328	2766	3181	3851	4235	3551	2749
	Unsafe water source	2461	382	2756	2647	3219	1400	1642	1406	2434	2963	2664	1730
	High systolic blood pressure	2451	3513	3578	2322	3181	2433	2932	2778	2294	2692	2306	2780
	Child growth failure	2201	460	2348	1998	3342	1244	933	1302	2320	3463	2174	1178
	Unsafe sanitation	1922	432	2168	2074	2513	1070	1330	1242	1901	2269	2067	1328
	High fasting plasma glucose	1719	2094	2552	1514	2137	1632	2049	1878	1591	1927	2036	1806
	Unsafe sex	1698	4361	3199	1870	1510	2886	4309	1677	1278	2171	1187	1736
	No access to hand washing facility	1522	474	1735	1502	2087	973	1096	1036	1517	1908	1668	1140
	Alcohol use	1485	1485	1485	1485	1485	1485	1485	1485	1485	1485	1485	1485
	High body-mass index	1071	2739	1336	874	1430	1622	1630	2009	930	765	1100	1310
	Kidney dysfunction	824	945	1122	729	1010	790	891	884	784	875	945	859
	Smoking	534	527	1207	226	718	1124	1677	1601	630	1426	615	181
	Occupational injuries	527	173	635	559	513	307	375	301	556	795	469	472

	High LDL cholesterol	495	774	799	457	702	507	642	576	426	551	526	557
**Risk factor-attributable DALYs, Female**	All risk factors	17770	16731	28523	16302	25379	16641	17187	15572	17581	24045	17459	16116
	Particulate matter pollution	3903	1877	6445	3563	6299	2936	2755	3107	3953	5562	4013	3748
	Low birth weight and short gestation	2940	1103	2629	3176	3536	2750	2206	2601	3046	3644	2596	2192
	High systolic blood pressure	2417	3086	4948	2018	4207	2296	1983	2590	2456	3035	2284	2772
	Unsafe sex	2367	6128	4519	2379	2252	3643	5934	1995	1893	3032	1727	2409
	Child growth failure	2204	403	2431	2068	3445	1215	849	1266	2301	3628	2168	1126
	Unsafe water source	2148	280	2850	2219	3087	1226	1233	1213	2210	2676	2369	1411
	Unsafe sanitation	1678	321	2243	1740	2411	938	999	1071	1726	2050	1839	1085
	High fasting plasma glucose	1462	1685	2871	1134	2512	1350	1085	1558	1480	1857	1639	1603
	No access to handwashing facility	1337	369	1825	1262	2077	839	763	889	1383	1760	1468	962
	High body-mass index	1098	2680	1865	794	1948	1652	1155	1991	1010	894	1085	1380
	Kidney dysfunction	732	838	1341	561	1201	688	523	767	758	847	816	770
	Iron deficiency	609	397	1315	388	702	803	479	612	605	1747	551	488
	Intimate Partner Violence	544	871	552	635	372	649	1063	421	516	311	470	443
	Diet High in Sodium	508	612	1087	426	913	477	424	542	494	678	522	601
	Alcohol use	489	725	1072	292	704	489	354	445	602	448	470	508

**Risk factor-attributable DALYs, Male**	All risk factors	22323	19008	25691	22050	24293	21196	27087	22287	20960	26234	23971	19560
	Particulate matter pollution	4944	2613	5475	4896	5411	3679	5608	3910	4641	5988	5481	4529
	Low birth weight and short gestation	4382	1560	4043	4199	4782	3876	3294	3730	4616	4791	4459	3277
	Unsafe water source	2765	495	2769	3088	3321	1587	2083	1624	2650	3160	2954	2061
	High systolic blood pressure	2487	4010	2901	2619	2409	2580	3952	2951	2156	2551	2330	2780
	Alcohol use	2462	3502	2490	2082	2605	2829	4217	3390	2229	2232	3357	2124
	Child growth failure	2199	522	2298	1930	3252	1276	1022	1339	2339	3350	2182	1228
	Unsafe sanitation	2159	556	2178	2417	2591	1213	1686	1434	2070	2419	2292	1580
	High fasting plasma glucose	1964	2569	2325	1902	1820	1936	3048	2252	1694	1963	2423	2017
	No access to handwashing facility	1700	593	1706	1750	2092	1116	1452	1204	1645	2006	1866	1326
	High body-mass index	1046	2795	1000	957	986	1584	2153	2013	854	693	1117	1237
	Unsafe sex	1030	2490	2069	1348	842	2117	2433	1360	679	1446	642	1016
	Smoking	975	1040	1845	423	1080	2122	3059	3148	1154	2231	1090	330
	Kidney dysfunction	911	1076	990	899	856	900	1269	1016	808	889	1071	950
	Occupational injuries	808	276	990	844	825	465	551	448	846	1160	735	730
	Diet low in fruits	534	674	651	550	521	505	848	586	439	573	609	586

Table S16. Leading risk factors attribution to all-cause age-standardised YLLs rate per 100 000 populations for Ethiopia, 9 regions and 2 cities, both sexes, all age, in 2019
YLLs=years of life lost

**Rank**	**Ethiopia**	**% of total YLL**	**Addis Ababa**	**% of total YLL**	**Afar**	**% of total YLL**	**Amhara**	**% of total YLL**
1	Particulate matter pollution	13.45%	Unsafe sex	16.30%	Particulate matter pollution	13.23%	Particulate matter pollution	13.06%
2	Low birth weight and short gestation	11.76%	High systolic blood pressure	13.29%	High systolic blood pressure	8.36%	Low birth weight and short gestation	12.10%
3	High systolic blood pressure	7.57%	High body-mass index	9.33%	Low birth weight and short gestation	7.89%	Unsafe water source	8.29%
4	Unsafe water source	7.54%	Particulate matter pollution	7.99%	Unsafe sex	7.40%	High systolic blood pressure	7.29%
5	Child growth failure	7.13%	High fasting plasma glucose	6.97%	Unsafe water source	6.34%	Child growth failure	6.58%
6	Unsafe sanitation	5.89%	Alcohol use	6.81%	Child growth failure	5.60%	Unsafe sanitation	6.49%
7	Unsafe sex	5.19%	Low birth weight and short gestation	4.60%	High fasting plasma glucose	5.30%	Unsafe sex	5.80%
8	No access to handwashing facility	4.79%	Kidney dysfunction	3.45%	Unsafe sanitation	4.99%	No access to handwashing facility	4.81%
9	High fasting plasma glucose	4.59%	Low temperature	3.00%	No access to handwashing facility	4.09%	High fasting plasma glucose	4.08%
10	Alcohol use	3.90%	High LDL cholesterol	2.96%	Alcohol use	3.75%	Alcohol use	2.99%
**Rank**	**B/Gumuz**	**% of total YLL**	**Dire Dawa**	**% of total YLL**	**Gambella**	**% of total YLL**	**Harari**	**% of total YLL**
1	Particulate matter pollution	13.46%	Low birth weight and short gestation	11.49%	Unsafe sex	12.44%	Particulate matter pollution	10.72%
2	Low birth weight and short gestation	10.19%	Particulate matter pollution	10.51%	Particulate matter pollution	11.96%	Low birth weight and short gestation	10.59%
3	Child growth failure	8.19%	Unsafe sex	9.35%	High systolic blood pressure	8.75%	High systolic blood pressure	8.90%
4	Unsafe water source	7.46%	High systolic blood pressure	8.04%	Low birth weight and short gestation	8.35%	High body-mass index	5.71%
5	High systolic blood pressure	7.45%	High body-mass index	4.70%	Alcohol use	5.91%	Alcohol use	5.25%
6	Unsafe sanitation	5.82%	High fasting plasma glucose	4.64%	High fasting plasma glucose	5.40%	High fasting plasma glucose	5.21%
7	No access to handwashing facility	4.97%	Alcohol use	4.64%	Smoking	4.75%	Unsafe sex	5.01%
8	High fasting plasma glucose	4.39%	Unsafe water source	4.48%	Unsafe water source	4.65%	Smoking	4.80%
9	Unsafe sex	3.45%	Child growth failure	4.32%	High body-mass index	4.33%	Child growth failure	4.37%
10	Alcohol use	3.45%	Smoking	3.44%	Unsafe sanitation	3.77%	Unsafe water source	4.33%
**Rank**	**Oromia**	**% of total YLL**	**Somali**	**% of total YLL**	**SNNP**	**% of total YLL**	**Tigray**	**% of total YLL**

1	Particulate matter pollution	14.07%	Particulate matter pollution	0.1431	Particulate matter pollution	13.11%	Particulate matter pollution	14.31%
2	Low birth weight and short gestation	13.28%	Low birth weight and short gestation	10.91%	Low birth weight and short gestation	0.1049	Low birth weight and short gestation	9.88%
3	Child growth failure	8.13%	Child growth failure	8.98%	Unsafe water source	7.50%	High systolic blood pressure	9.85%
4	Unsafe water source	8.04%	Unsafe water source	7.33%	High systolic blood pressure	6.61%	Unsafe sex	6.02%
5	High systolic blood pressure	7.57%	High systolic blood pressure	6.69%	Child growth failure	6.47%	Unsafe water source	5.90%
6	Unsafe sanitation	6.28%	Unsafe sanitation	5.61%	Unsafe sanitation	5.83%	High fasting plasma glucose	5.57%
7	No access to handwashing facility	5.16%	Unsafe sex	5.48%	High fasting plasma glucose	5.06%	Unsafe sanitation	4.53%
8	High fasting plasma glucose	4.53%	No access to handwashing facility	4.85%	Alcohol use	4.85%	Child growth failure	4.33%
9	Unsafe sex	4.19%	High fasting plasma glucose	4.17%	No access to handwashing facility	4.83%	High body-mass index	4.14%
10	Alcohol use	4.01%	Smoking	3.32%	Unsafe sex	3.35%	No access to handwashing facility	4.05%

Table S17. Top 10 leading causes of national age-standardised death, YLL, YLD and DALY rates per 100,000 for both sexes in Ethiopia in 2019, and the percent change of variation from 1990

**Age standardized death rate**				
	National rate 2019	*Mean % change of variation from 1990-2019*	*Highest region*	*Lowest region*
Stroke	90.18(70.62-110.04)	-41%		
Lower respiratory infections	86.44(75.36-97.65)	-61%		Addis Ababa
Ischemic heart disease	84.24(62.56-105.75)	-24%		
Diarrheal diseases	76.45(45.12-112.15)	-75%		Addis Ababa
Tuberculosis	60.9(50.44-71.5)	-81%	Afar, Somali, B-Gumuz	
Cirrhosis and other chronic liver diseases	52.18(44.17-62.07)	-32%		
Neonatal disorders	44.31(34.92-56.99)	-43%		Addis Ababa
Diabetes mellitus	36(31.01-41.43)	-38%		
HIV/AIDS*	33.56(28.68-39.58)	-83.52%	Afar, Gambella, Addis Ababa	
Hypertensive heart disease	31.97(17.85-53.09)	-45%		

**Age-standardized YLL rate**				
	National rate 2019	*Mean % change of variation from 1990-2019*	*Highest region*	*Lowest region*
Neonatal disorders	3936(3102.02-5062.36)	-43%		Addis Ababa
Diarrheal diseases	2679.41(1823.93-3760.22)	-76%		Addis Ababa
Lower respiratory infections	2404.57(2059.41-2833.31)	-76%		Addis Ababa
Tuberculosis	1729.19(1421.57-2049.86)	-83%	Afar, Somali, B-Gumuz	
Stroke	1639.45(1277.27-1998.83)	-51%	Afar	
HIV/AIDS*	1581.17(1311.51-1935.43)	-84.35%	Dire Dawa, Gambella Addis Ababa	
Ischemic heart disease	1524.69(1133.11-1925.66)	-38%		
Cirrhosis and other chronic liver diseases	1331.56(1095.91-1625.94)	-38%		
Congenital birth defects	735.83(447.45-1200.08)	-58%		
Diabetes mellitus	706.24(606.76-813.68)	-49%		

**Age-standardized YLD rate**				
	National rate 2019	*Mean % change of variation from 1990-2019*	*Highest region*	*Lowest region*
Depressive disorders	808.7(562.26-1115.11)	-9%		
Low back pain	700.06(491.31-935.19)	-1%		
Age-related and other hearing loss	552.7(377.77-779.89)	-5%		
Gynecological diseases	470.78(322.13-659.38)	-2%		
Blindness and vision loss	420.96(303.1-568.56)	-12%		
Dietary iron deficiency	396.03(261.75-579.23)	-24%	Somalia	
Headache disorders	353.16(95.74-736.24)	-1%		

Anxiety disorders	331.14(230.89-451.16)	-4%		
Neonatal disorders	307.52(235.31-386.04)	603%		
Diabetes mellitus	273.67(189.12-376.26)	-6%		

Age-standardized DALY rate				
	National rate 2019	*Mean % change of variation from 1990-2019*	*Highest region*	*Lowest region*
Neonatal disorders	4243.53(3407.87-5383.87)	-39%		Addis Ababa
Diarrheal diseases	2898.12(2035.97-3973.63)	-75%		Addis Ababa
Lower respiratory infections	2415.69(2067.8-2845.72)	-76%		Addis Ababa
Tuberculosis	1853.05(1539.82-2163.82)	-82%	Afar,<br>B/Gumuz<br>Somali	
Stroke	1777.86(1411.33-2139.63)	-50%	Afar	
HIV/AIDS*	1699.06(1423.54-2050.66)	-83.83%	Addis Ababa,<br>Afar,<br>Dire Dawa,<br>Gambella	
Ischemic heart disease	1579.98(1184.72-1974)	-36%		
Cirrhosis and other chronic liver diseases	1341.44(1106.26-1637.42)	-38%		
Diabetes mellitus	979.91(849.79-1115.68)	-41%		
Congenital birth defects	829.43(540.82-1298.86)	-56%		

Table S18. GATHER Checklist for "Progress in health among regions of Ethiopia, 1990-2019: a sub-national country analysis for the Global Burden of Disease Study 2019"

#	Checklist item	Description of compliance	Reference
	Objectives and funding		
1	Define the indicator(s), populations (including age, sex, and geographic entities), and time period(s) for which estimates were made.	Narrative provided in paper providing description of indicators, population, definitions, and time period	Main text
2	List the funding sources for the work.	Funding source listed in paper	Abstract (Funding)
	Data Inputs		
	For all data inputs from multiple sources that are synthesized as part of the study:		
3	Describe how the data were identified and how the data were accessed.	Narrative description of data seeking m ethods provided	Main text (Methods) and meth ods appendix
4	Specify the inclusion and exclusion criteria. Identify all ad-hoc exclusions.	Narrative about inclusion and exclusion criteria by data type provided	Main text (Methods) and meth ods appendix
5	Provide information on all included data sources and their main characteristics. For each data source used, report reference information or contact name/institution, population represented, data collection method, year(s) of data collection, sex and age range, diagnostic criteria or measurement method, and sample size, as relevant.	An interactive, online data source tool th at provides metadata for data sources by component, geography, cause, risk, or impairment has been developed	Online data citation tools
6	Identify and describe any categories of input data that have potentially important biases (e.g., based on characteristics listed in item 5).	Summary of known biases by cause incl uded in methods appendix	Methods appendix
	For data inputs that contribute to the analysis but were not synthesized as part of the study:		
7	Describe and give sources for any other data inputs.	Included in online data source tool, http://ghdx.healthdata.org/gbd-2019	Online data citation tools
	For all data inputs:		
8	Provide all data inputs in a file format from which data can be efficiently extracted (e.g., a spreadsheet rather than a PDF), including all relevant meta-data listed in item 5. For any data inputs that cannot be shared because of ethical or legal reasons, such as third-party ownership, provide a contact name or the name of the institution that retains the right to the data.	Downloads of input data available throug h online tools, including data visualizatio n tools and data query tools, http://ghdx.healthdata.org/gbd-2019; input data not available in tools w ill be made available	Online data visualization tools, data query tools, and the Globa l Health Data Exchange, http://ghdx.healthdata.org/gbd-2019
	Data analysis		
9	Provide a conceptual overview of the data analysis method. A diagram may be helpful.	Flow diagrams of the overall methodolo gical processes, as well as cause-specific modelling processes, have been provided	Main text (Methods) and meth ods appendix
10	Provide a detailed description of all steps of the analysis, including mathematical formulae. This description should cover, as relevant, data cleaning, data pre-processing, data adjustments and weighting of data sources, and mathematical or statistical model(s).	Flow diagrams and corresponding meth odological write-ups for each cause, as well as the demo graphics and causes of death databases and modelling processes, have been pro vided	Main text (Methods) and meth ods appendix
11	Describe how candidate models were evaluated and how the final model(s) were selected.	Provided in the methodological write-ups	Methods appendix
12	Provide the results of an evaluation of model performance, if done, as well as the results of any relevant sensitivity analysis.	Provided in the methodological write-ups	Methods appendix
13	Describe methods for calculating uncertainty of the estimates. State which sources of uncertainty were, and were not, accounted for in the uncertainty analysis.	Provided in the methodological write-ups	Methods appendix
14	State how analytic or statistical source code used to generate estimates can be accessed.	Access statement provided	Code is provided in an online r epository

	Results and Discussion		
15	Provide published estimates in a file format from which data can be efficiently extracted.	Results are available through online dat a visualization tools, the Global Health Data Exchange, and the online data quer y tool http://ghdx.healthdata.org/gbd-2019	Main text, methods appendix, and online data tools (data vis ualization tools, data query too ls, and the Global Health Data Exchange, http://ghdx.healthd ata.org/gbd-2019
16	Report a quantitative measure of the uncertainty of the estimates (e.g. uncertainty intervals).	Uncertainty intervals are provided with all results	Main text, methods appendix, and online data tools (data 301 visualization tools, data query t ools, and the Global Health Dat a Exchange, http://ghdx.health data.org/gbd-2019
17	Interpret results in light of existing evidence. If updating a previous set of estimates, describe the reasons for changes in estimates.	Discussion of methodological changes between GBD rounds provided in the n arrative of the Article and methods appe ndix	Main text (Methods and Discu ssion) and methods appendix
18	Discuss limitations of the estimates. Include a discussion of any modelling assumptions or data limitations that affect interpretation of the estimates.	Discussion of limitations provided in th e narrative of the main paper, as well as in the methodological write-ups in the methods appendix	Main text (Limitations) and m ethods appendix

Section 8: References

1. Central Statistical Agency (Ethiopia). Ethiopia Population and Housing Census 1984.

2. Central Statistical Agency (Ethiopia), United Nations Population Fund (UNFPA), United States Agency for International Development (USAID). Ethiopia Population and Housing Census 1994.

3. Central Statistical Agency (Ethiopia), Government of Ethiopia, United Nations Population Fund (UNFPA), United Nations Development Programme (UNDP). Ethiopia Population and Housing Census 2007. Addis Ababa, Ethiopia: Central Statistical Agency (Ethiopia).

4. Wang H, Abbas KM, Abbasifard M, Abbasi-Kangevari M, Abbastabar H, Abd-Allah F, et al. Global age-sex-specific fertility, mortality, healthy life expectancy (HALE), and population estimates in 204 countries and territories, 1950–2019: a comprehensive demographic analysis for the Global Burden of Disease Study 2019. The Lancet [Internet]. 2020 Oct 17 [cited 2021 Apr 15];396(10258):1160–203. Available from: https://www.thelancet.com/journals/lancet/article/PIIS0140-6736(20)30977-6/abstract

5. Vos T, Lim SS, Abbafati C, Abbas KM, Abbasi M, Abbasifard M, et al. Global burden of 369 diseases and injuries in 204 countries and territories, 1990–2019: a systematic analysis for the Global Burden of Disease Study 2019. The Lancet [Internet]. 2020 Oct 17 [cited 2021 Apr 15];396(10258):1204–22. Available from: https://www.thelancet.com/journals/lancet/article/PIIS0140-6736(20)30925-9/abstract

6. Achoki T, Miller-Petrie MK, Glenn SD, Kalra N, Lesego A, Gathecha GK, et al. Health disparities across the counties of Kenya and implications for policy makers, 1990–2016: a systematic analysis for the Global Burden of Disease Study 2016. The Lancet Global Health [Internet]. 2019 Jan 1 [cited 2021 Nov 28];7(1):e81–95. Available from: https://www.sciencedirect.com/science/article/pii/S2214109X18304728

7. James SL, Abate D, Abate KH, Abay SM, Abbafati C, Abbasi N, et al. Global, regional, and national incidence, prevalence, and years lived with disability for 354 diseases and injuries for 195 countries and territories, 1990–2017: a systematic analysis for the Global Burden of Disease Study 2017. The Lancet [Internet]. 2018 Nov 10 [cited 2021 Apr 15];392(10159):1789–858. Available from: https://www.thelancet.com/journals/lancet/article/PIIS0140-6736(18)32279-7/abstract

8. Dicker D, Nguyen G, Abate D, Abate KH, Abay SM, Abbafati C, et al. Global, regional, and national age-sex-specific mortality and life expectancy, 1950–2017: a systematic analysis for the Global Burden of Disease Study 2017. The Lancet [Internet]. 2018 Nov 10 [cited 2021 Apr 15];392(10159):1684–735. Available from: https://www.thelancet.com/journals/lancet/article/PIIS0140-6736(18)31891-9/abstract

9. Foreman KJ, Lozano R, Lopez AD, Murray CJ. Modeling causes of death: an integrated approach using CODEm. Population Health Metrics [Internet]. 2012 Jan 6 [cited 2021 Apr 16];10(1):1. Available from: https://doi.org/10.1186/1478-7954-10-1

10. Naghavi M, Abajobir AA, Abbafati C, Abbas KM, Abd-Allah F, Abera SF, et al. Global, regional, and national age-sex specific mortality for 264 causes of death, 1980–2016: a systematic analysis for the Global Burden of Disease Study 2016. The Lancet [Internet]. 2017 Sep 16 [cited 2021 Apr 16];390(10100):1151–210. Available from: https://www.thelancet.com/journals/lancet/article/PIIS0140-6736(17)32152-9/abstract

11. Kyu HH, Abate D, Abate KH, Abay SM, Abbafati C, Abbasi N, et al. Global, regional, and national disability-adjusted life-years (DALYs) for 359 diseases and injuries and healthy life expectancy (HALE) for 195 countries and territories, 1990–2017: a systematic analysis for the Global Burden of Disease Study 2017. The Lancet [Internet]. 2018 Nov 10 [cited 2021 Apr 16];392(10159):1859–922. Available from: https://www.thelancet.com/journals/lancet/article/PIIS0140-6736(18)32335-3/abstract

12. Hay SI, Abajobir AA, Abate KH, Abbafati C, Abbas KM, Abd-Allah F, et al. Global, regional, and national disability-adjusted life-years (DALYs) for 333 diseases and injuries and healthy life expectancy (HALE) for 195 countries and territories, 1990–2016: a systematic analysis for the Global Burden of Disease Study

2016. The Lancet [Internet]. 2017 Sep 16 [cited 2021 Apr 16];390(10100):1260–344. Available from: https://www.thelancet.com/journals/lancet/article/PIIS0140-6736(17)32130-X/abstract

13. Stanaway JD, Afshin A, Gakidou E, Lim SS, Abate D, Abate KH, et al. Global, regional, and national comparative risk assessment of 84 behavioural, environmental and occupational, and metabolic risks or clusters of risks for 195 countries and territories, 1990–2017: a systematic analysis for the Global Burden of Disease Study 2017. The Lancet [Internet]. 2018 Nov 10 [cited 2021 Apr 15];392(10159):1923–94. Available from: https://www.thelancet.com/journals/lancet/article/PIIS0140-6736(18)32225-6/abstract

14. Murray CJL, Aravkin AY, Zheng P, Abbafati C, Abbas KM, Abbasi-Kangevari M, et al. Global burden of 87 risk factors in 204 countries and territories, 1990–2019: a systematic analysis for the Global Burden of Disease Study 2019. The Lancet [Internet]. 2020 Oct 17 [cited 2021 Apr 15];396(10258):1223–49. Available from: https://www.thelancet.com/journals/lancet/article/PIIS0140-6736(20)30752-2/abstract
